# Bottom-Up Fabrication of BN-Doped Graphene Electrodes from Thiol-Terminated Borazine Molecules Working in Solar Cells

**DOI:** 10.1021/acsami.4c23116

**Published:** 2025-04-02

**Authors:** Carolina M. Ibarra-Barreno, Sanchari Chowdhury, Martina Crosta, Tashfeen Zehra, Francesco Fasano, Paromita Kundu, Jenthe Verstraelen, Sara Bals, Mohammed Subrati, Davide Bonifazi, Rubén D. Costa, Petra Rudolf

**Affiliations:** † Zernike Institute for Advanced Materials, University of Groningen, Nijenborgh 3, Groningen 9747 AG, Netherlands; ‡ Technical University of Munich, Campus Straubing for Biotechnology and Sustainability, Chair of Biogenic Functional Materials, Schulgasse 22, Straubing 94315, Germany; § Institute of Organic Chemistry, Faculty of Chemistry, University of Vienna, Vienna 1090, Austria; ∥ School of Chemistry, Cardiff University, Park Place, Main Building, Cardiff CF10 3AT, United Kingdom; ⊥ Electron Microscopy for Materials Research (EMAT), Faculty of Science/Department of Physics Campus Groenenborger U407, University of Antwerp, Groenenborgerlaan 171, Antwerp 2020, Belgium; # Institute of Nanoscience and Nanotechnology, National Center for Scientific Research ‘Demokritos’, Agia Paraskevi, Attica 15310, Greece

**Keywords:** BN-doped graphene, borazine, self-assembly, photopolymerization, indoor and outdoor energy harvesting, dye-sensitized solar cells

## Abstract

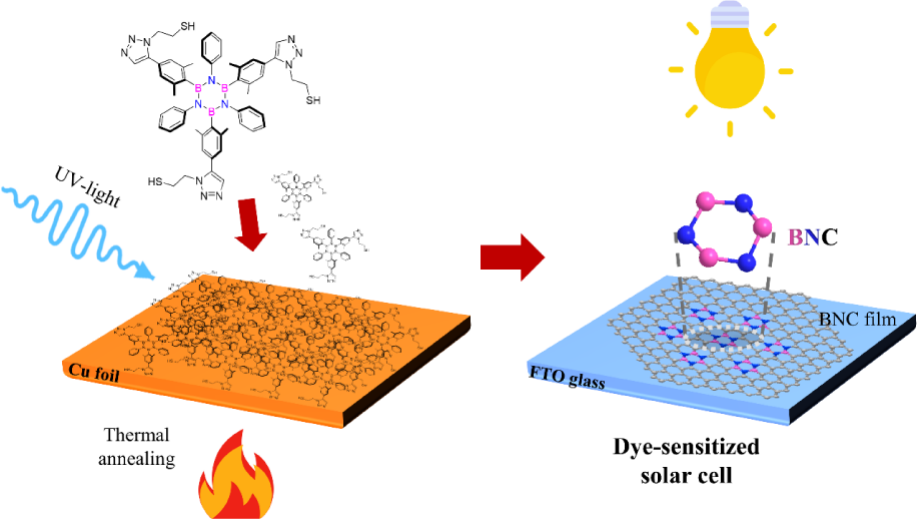

Graphene exhibits exceptional properties, including high tensile strength, mechanical stiffness, and electron mobility. Chemical functionalization of graphene with boron and nitrogen is a powerful strategy for tuning these properties for specific applications. Molecular self-assembly provides an efficient pathway for the tailored synthesis of doped graphene, depending on the molecular precursor used. This study presents a scalable approach to synthesizing large-area boron- and nitrogen-doped graphene using two borazine precursors bearing thiol functionalities. After self-assembly on electropolished polycrystalline copper foil, the precursors undergo photopolymerization under UV irradiation, and subsequent annealing in vacuum transforms the cross-linked BN-doped layer into a graphenoid structure. X-ray photoelectron spectroscopy confirms the integration of the borazine rings into the BNC architecture, while Raman spectroscopy reveals a red shift in the characteristic G bands along with intense and broad D bands, highlighting boron–nitrogen contributions. Transmission electron microscopy provides insight into the morphology and structural quality of the BNC films. The BNC films were successfully integrated as counter electrodes in dye-sensitized solar cells, achieving a power conversion efficiency of up to 6% under 1 sun illumination and 11.8% under low-intensity indoor ambient light. Hence, this work not only establishes a straightforward, controllable route for heteroatom doping but also introduces a novel concept of Pt-free counter electrodes for efficient indoor energy harvesting applications.

## 1 Introduction

With its extraordinary electronic, mechanical, and thermal properties,^1^ graphene has emerged as a key 2D material for optoelectronics, fuel cells, energy storage devices (supercapacitors), sensors, and photovoltaic devices.^2−5^ However, one of the major limiting factors for the full-scale implementation of graphene in device applications is its gapless semiconducting character. A promising approach to address this challenge is heteroatom doping, which can modify graphene’s semiconducting properties by introducing boron (B) or nitrogen (N) atoms into the graphene lattice.^6,7^ Various experimental and theoretical studies have shown that doping graphene with these atoms converts it into a *p*- or *n*-type semiconductor whose band gap and transport properties can be tuned by varying the dopant concentration.^8,9^ Borazine rings (B_3_N_3_) present an ideal dopant structure, as they are isostructural and isoelectronic to graphene’s carbon rings, and their bond length (1.44 Å) is nearly identical to that of benzene (1.40 Å).^10^ These similarities allow borazine to integrate seamlessly into the graphene lattice, where the electron-donating nitrogen atoms and the electrophilic boron atoms give rise to unique electronic properties (refs (11, 12) and references therein), such as a widened HOMO–LUMO gap or a reduced HOMO energy compared to benzene.^11^ In this context, borazine can be an attractive choice for graphene doping when aiming for applications in UV-emitting OLEDs, H_2_ storage, ceramics, coatings, sensors, or catalysis.^10,13,14^

However, despite promising optoelectronic properties, the practical implementation of a BN-doped graphene film in photovoltaic devices as electrode components remained problematic due to several factors. First, the fabrication and implementation of heteroatom-doped graphene require complicated synthesis and transfer steps that present a high risk of introducing defects and tears in the film, which degrade its quality.^15,16^ In addition, contact with various organic/inorganic solvents in the device architecture significantly reduces the film’s electrical conductivity, surface area, and optical transparency. This behavior deteriorates mass transfer kinetics, which makes the application of graphene-based films in photovoltaic and/or other optoelectronic devices still a key challenge.^17,18^ As leading examples, implementing B-doped reduced graphene oxide as hole transport layers in perovskite solar cells exhibited a power conversion efficiency (η) of up to 8.96% and N-doped graphene, codoped with zinc oxide nanorods as an electron transport layer, achieved an η of up to 16.82%.^4,19^ However, both designs required rather complicated fabrication steps and the addition of metallic nanorods to enhance efficiency.^4,19^ In another type of photovoltaic device, dye-sensitized solar cells (DSSCs) have reached η values of up to 6.73% and 9.38% when B-doped reduced graphene oxide^20^ and N-doped graphene on a platinum film sputtered onto a fluorine-doped tin oxide (FTO) substrate^21^ were employed as counter electrodes (CEs), respectively. Here, the time-consuming preparation, low production yield, and requirement of toxic precursors still call for improvement. Hence, implementing a BN-codoped film, produced in a simpler and more sustainable approach, as a CE in DSSCs represents a frontier solution that is aimed for in this work.

Doping of graphene is still in its early stages, and no mainstream method comparable to chemical vapor deposition for the growth of pure graphene exists.^6,9,22,23^ Current methods for preparing BN-doped graphene often rely on multistep chemical routes and hydrothermal processes, which limit their scalability.^22,24^ Initial efforts using chemical vapor deposition to codoped graphene have encountered issues with dopant segregation, where BN nanodomains formed in the carbon sheet.^6^ To mitigate segregation, Xu et al. used plasma treatment to create defects in graphene, which were filled with dopants by thermal annealing in the presence of borazine.^9^ However, this approach yielded only low dopant concentrations. Another attempt to synthesize codoped graphene via the decomposition of hexaphenylborazine on heated metallic substrates has been hindered by the loss of dopant atoms at relatively low annealing temperatures (402 °C).^23^

**Figure 1:**
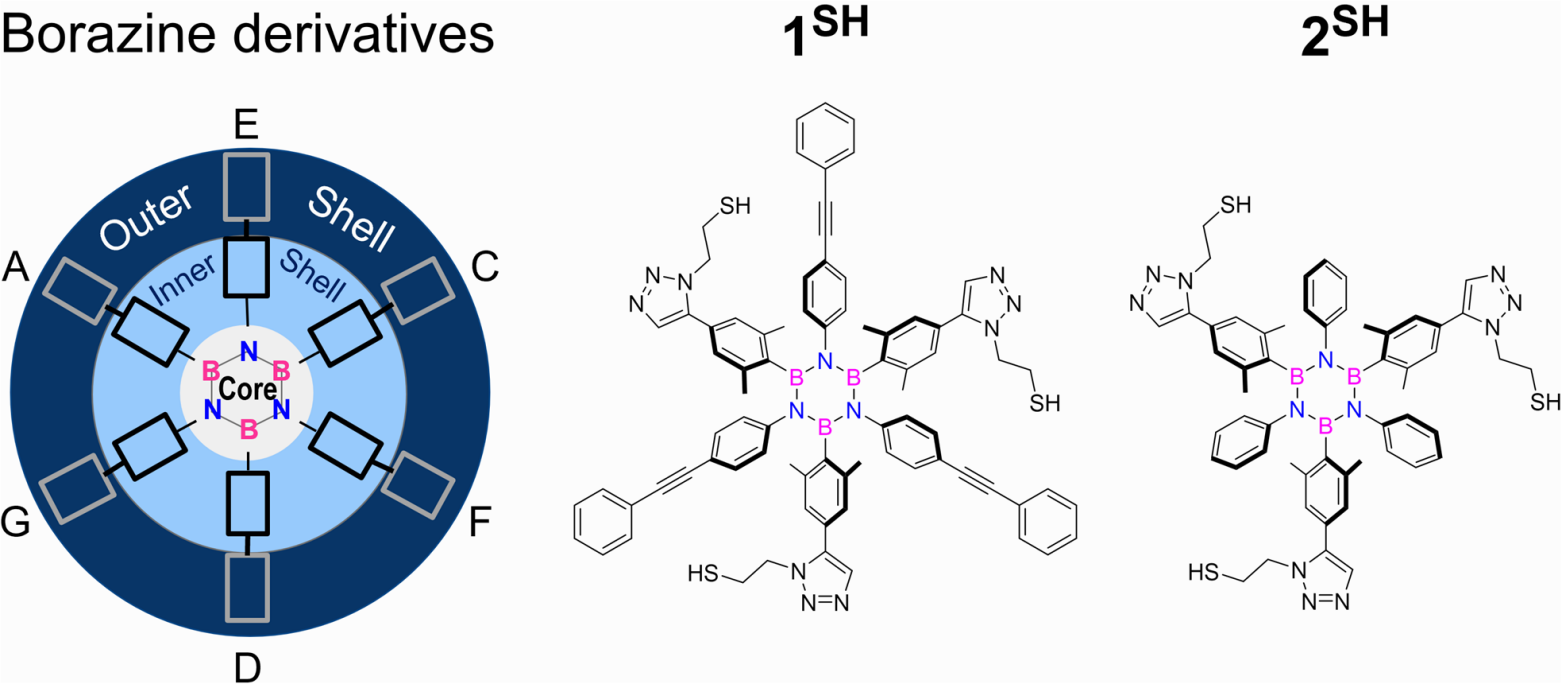
General scheme of a borazine derivative (left).^10^ Chemical structure of thiol-terminated borazines **1**^**SH**^ and **2**^**SH**^.

Herein, we report an alternative bottom-up approach for synthesizing B- and N-codoped graphene, utilizing thiolated BN-containing precursors (see Figure 1). Starting with a molecular precursor bearing a preformed BN core, *i.e*., a borazine ring, is key to avoiding dopant segregation or the loss of dopant atoms during synthesis. Borazine derivatives can easily be synthesized through various organic reactions (*e.g.,* thermal cleavages, weak bases, hydrogenolysis, *etc.*).^10,25^ The B_3_N_3_ core motif allows for the attachment of various functional groups to each nitrogen and boron atom, forming inner and outer shells depending on the chemical nature of the substituents. These functional groups are critical for stabilizing the borazine core, which is inherently sensitive to hydrolysis, and they can influence both the synthesis process and the material’s properties, expanding its potential applications. The borazine derivatives used in this study are thiol borazines **1**^**SH**^ and **2**^**SH**^ (Figure 1). These molecules serve as starting materials for the self-assembly, where the thiol groups anchor the molecules to copper foil. The self-assembled layer is then irradiated with UV light to induce cross-linking and prevent sublimation during subsequent annealing in a vacuum to transform it into doped graphene (Scheme S1). Previous studies by Turchanin^26^ (and references therein) have demonstrated the growth of graphene by UV light- or electron-induced polymerization and annealing of self-assembled monolayers (SAMs). This work aims to adapt this method to self-assembled borazine molecular precursors to produce codoped graphene with boron and nitrogen integrated into the lattice.

After sufficient experimental evidence was collected to confirm that this goal was achieved, the next step was to test a device application by implementing the BN-doped graphene (BNC) film as a CE in DSSCs (Scheme S2). Before realizing the device, the electrocatalytic behavior was tested and found to be propitious. DSSCs with the BN-doped graphene film as the CE exhibited a promising η of up to 6% under standard conditions (AM 1.5G, 1 sun illumination) and 11.8% under indoor illumination. The latter resulted in a significant indoor energy harvesting power of up to 235 μW/cm^2^. To our knowledge, this is the first BN-doped graphene-based CE effectively operating under low-light conditions, while under standard conditions, this device performs slightly better than a recent type of BNC-CE prepared by laser induction technology (5% DSSC power conversion efficiency).^27,28^ Extensive research has already been conducted to improve the catalytic behavior of counter electrodes.^29−32^ The present work makes a significant contribution by demonstrating promising performance under outdoor and indoor conditions, with ample potential for further optimization.

Hence, this work highlights two major findings. First, it demonstrates a straightforward method for preparing BNC layers and the simpler transfer techniques of such layers onto the FTO substrate, which might pave the way for transfer onto large-size substrates. Second, it proves that DSSCs with BN-doped graphene-based CEs deliver a promising performance under both standard and low-light conditions, thereby offering a viable pathway toward developing Pt-free, sustainable counter electrodes for next-generation DSSCs.

## 2 Results and Discussion

### 2.1 Synthesis of Thiol Borazines

**Scheme 1:**
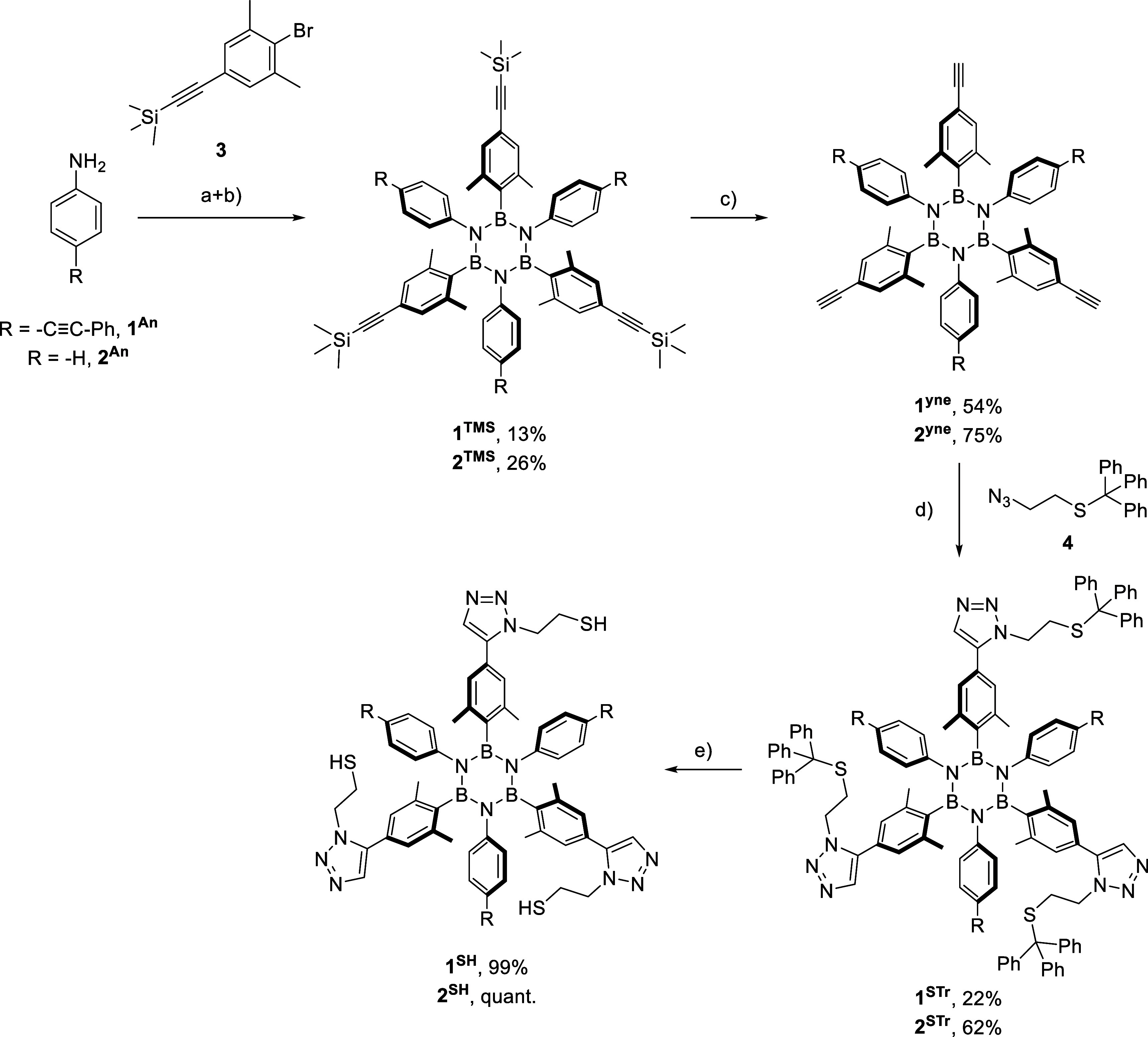
Synthesis of Thiol Borazines Reagents and conditions: (a) BCl_3_, toluene, reflux, 18 h; (b) **3**, *t*BuLi, THF, −84 °C to room temperature (rt), 16 h; (c) TBAF, THF, 0 °C to rt, 1 h; (d) sodium ascorbate, CuSO_4_·5H_2_O, DMF, H_2_O, rt, 16–40 h; (e) TFA, TIPS, CH_2_Cl_2_, 0 °C to rt, 1–3 h.

The synthetic strategy to access thiol borazines **1**^**SH**^ and **2**^**SH**^ has been envisaged through the click reaction of the relative borazine precursors bearing peripheral ethynyl groups **1**^**yne**^ and **2**^**yne**^ with (2-azidoethyl)(trityl)sulfane **4**,^33^ a key step to install thiol groups on the outer shell of the borazines (Scheme 1). Borazine precursors were prepared from 4-(phenylethynyl)aniline (**1**^**An**^) and aniline (**2**^**An**^) upon reaction with BCl_3_ under refluxing conditions.^34−36^ Treatment of the resulting *B*,*B′*,*B″*-trichloro-*N*,*N*′,*N*″-triaryl borazines with the trimethylsilyl (TMS)-protected aryllithium derived from **3**(37) afforded TMS-protected borazines **1**^**TMS**^ and **2**^**TMS**^ in 13% and 26% yield, respectively. Subsequent removal of the TMS protecting groups with tetra-*n*-butylammonium fluoride (TBAF) produced alkyne-terminated borazines **1**^**yne**^ and **2**^**yne**^ with 54% and 75% yields, respectively.^37^ The terminal 2,6-dimethylphenyl-4-acetylene moieties at the boron sites enabled the introduction of the trityl-protected thiol groups through Cu-catalyzed cycloaddition with **4**, affording borazines **1**^**STr**^ and **2**^**STr**^ in 22% and 62% yield, respectively. Ultimately, trityl deprotection with TFA and triisopropylsilane (TIPS) gave access to thiol borazines **1**^**SH**^ and **2**^**SH**^ in quantitative yields.

### 2.2 X-Ray Photoelectron Spectroscopy Characterization

#### 2.2.1 Film Growth from Thiol Borazine Precursor **1**^**SH**^

**Figure 2:**
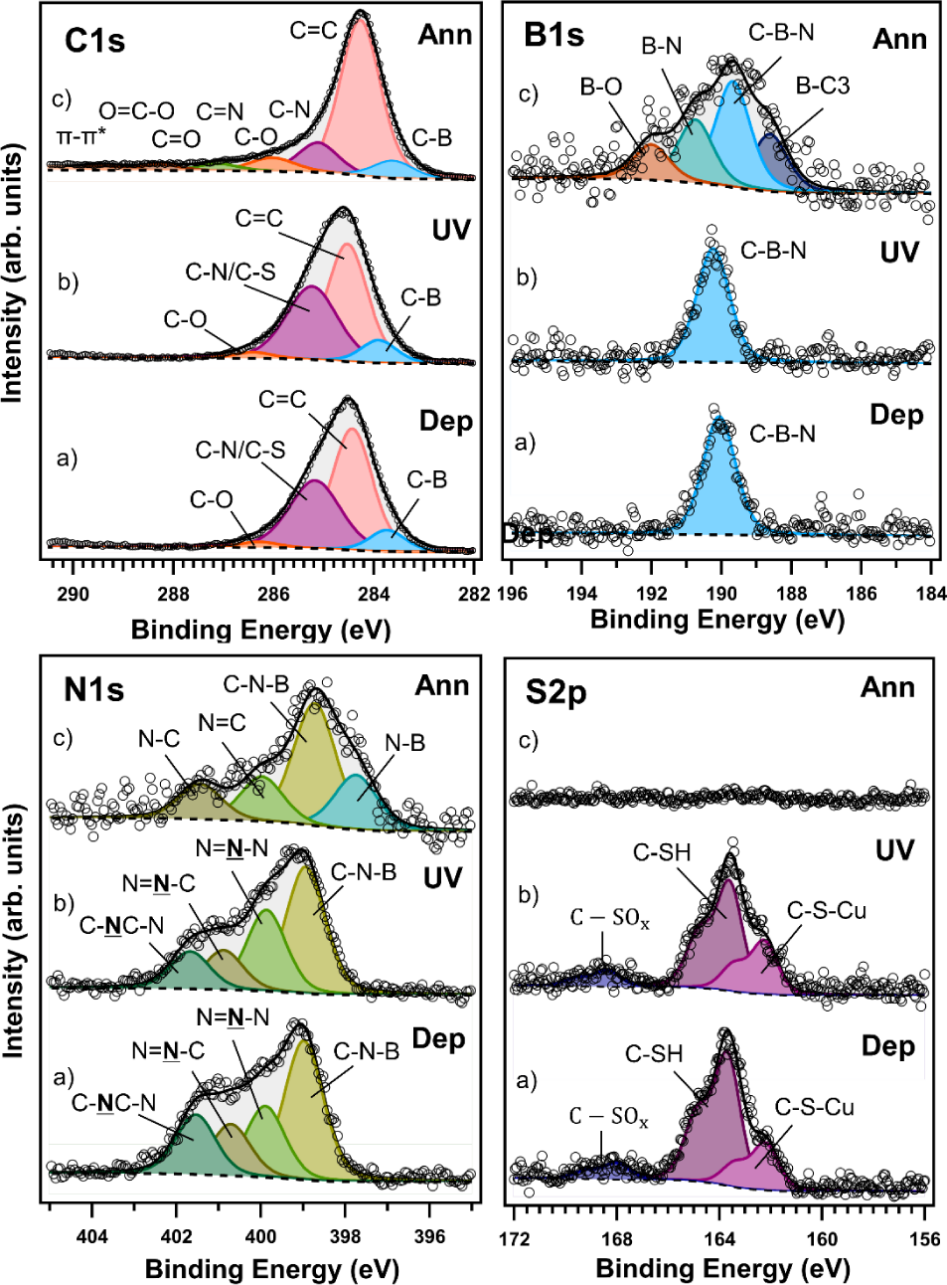
XPS spectra of the C 1s (top left panel), B 1s (top right panel), N 1s (bottom left panel), and S 2p (bottom right panel) core level regions of a self-assembled monolayer of a thiol-terminated borazine derivative **1**^**SH**^ on electropolished copper foil after deposition (a), after UV light-induced polymerization (b) and annealing (c). The corresponding fits are shown as well. For the discussion of the color-coded components see text.

To ascertain that the molecular precursor self-assembled without degradation on electropolished copper foil and could be processed to yield BN-doped graphene, we used X-ray photoelectron spectroscopy (XPS). Figure 2 shows the spectra of the C 1s, B 1s, N 1s, and S 2p core level regions collected for **1**^**SH**^ SAM after deposition (a), after UV light-induced polymerization (b), and after annealing to transform it to BN-doped graphene (c). After deposition, the C 1s region (Figure 2 top left panel, a) can be deconvoluted into four components: the main component was observed at a binding energy (BE) of 284.4 eV, which corresponds to sp^2^ aromatic carbon–carbon bonds; additional components at BEs of 283.7 and 285.1 eV stem from C–B and C–N/C–S bonds, respectively, while the last contribution at 286.2 eV corresponds to C–O, likely due to surface contamination during the sample transfer.^6,7,38^ The B 1s photoemission signal for the same as-prepared self-assembled layer (Figure 2 top right panel, a) is peaked at 190.0 eV, as expected for the C–B–N_2_ structure of the borazine core.^6,39^ The absence of additional components in the B 1s spectrum after self-assembly suggests that the central borazine ring remains intact after deposition. The N 1s spectrum (Figure 2 bottom left panel, a) requires four components for a good fit: the one at a BE of 398.9 eV corresponds to C–N–B_2_ (nitrogen bound to boron in the thiol-borazine ring). The additional components at 399.8 eV, 400.7 eV, and 401.5 eV originated from N=**N**–N (nitrogen bonding with two nitrogens), N=**N**–C (nitrogen bonding with carbon), and C–**N**C–N (three-coordinated nitrogen); these peaks are consistent with the triazole aryl moiety from the molecule.^6,39,40^ The higher intensity of the first peak (borazine ring) compared to triazole components is probably due to X-ray-induced degradation during XPS analysis, which has been reported in other work.^40^ The S 2p region (Figure 2 bottom right panel, a) has three components. The main peak located at a BE of 163.6 eV corresponds to C–SH (free thiol groups not bound to Cu). Another component peaked at 162.2 eV, which is attributed to C–S–Cu (copper sulfides) and hence confirms that a layer of covalently anchored, integer borazine derivatives was produced. Although the self-assembly was carried out in a desiccator in the dark, a small peak at 167.9 eV due to C–SO_*x*_ (oxidized sulfur) is detected, possibly from adsorbed oxygen.^41^ The composition of the self-assembled layer was deduced from the XPS intensities of 78.7 at % carbon, 3.2 at % boron, 9.7 at % nitrogen, 3.9 at % sulfur, and 4.5 at % oxygen, which agrees with the stoichiometry of the molecule.

The next step in the synthesis of BN-doped graphene involved inducing photopolymerization of the adsorbed molecules. Without polymerization, the borazine derivatives would likely desorb from the surface during annealing rather than converting into graphene, as previously observed for biphenyl thiol (BPT).^42^ Based on prior optimized experiments with BPT exposed to UV radiation,^42^ an irradiation time of 6 h was chosen (see Experimental Section). In the XPS spectra of the UV-irradiated (polymerized) sample (see Figure 2, b), the main components of all spectral lines exhibited a slight shift of 0.2 eV toward higher BE, indicating a change in the chemical environment, as expected when dehydrogenation takes place and a cross-linked structure is formed.^41^ The C 1s spectrum (Figure 2 top left panel, b) was deconvoluted into the same four components discussed for the spectrum taken after self-assembly. The S 2p spectrum (Figure 2 bottom right panel, b) shows that the relative spectral intensity of the C–SH component decreased from 67% (after deposition) to 61% (after UV irradiation), while the C–S–Cu peak increased from 24% (after deposition) to 30% (after UV irradiation), proving that at least one out of three anchoring groups of the molecules chemisorbed on the surface. In the B 1s spectrum (Figure 2 top right panel, b), the line shape did not change after UV irradiation, implying no major changes in the bonding environment. In the N 1s spectrum (Figure 2 bottom left panel, b), the relative intensities of the components associated with N =N–C and C–NC–N moieties decreased by 8% after UV irradiation.

After polymerization, the sample was transferred to a vacuum furnace and annealed at 827 °C for 2 h to convert the polymerized SAM into a graphitic layer. After this heat treatment, we moved the sample back to the UHV system, where it was quickly annealed at 250 °C to desorb any contaminants and then taken to the photoemission spectrometer to collect again the C 1s, N 1s, B 1s, and S 2p XPS spectra.

Comparing the C 1s core-level region of the XPS spectrum after annealing with that of the polymerized SAM of **1**^**SH**^ shown in Figure 2 top left panel, (b) and (c), one notes that the main component of the C 1s line is now peaked at a BE of 284.2 eV, a slightly lower binding energy than that of CVD-grown graphene^43^ but still typical for graphitic carbon.^44^ The smaller contribution at lower BE (283.6 eV) corresponding to C–B is still present. At higher BEs, there are contributions at 285.1 eV, now attributed to C–N alone because there is no more S in the layer (see S 2p core-level region, Figure 2 bottom right panel, c); at 286.0 eV assigned to C–O, even if slightly shifted. Moving further to higher BE, there are new components at 287.1 eV, 288.2 eV, 289.4 eV, and 291.0 eV, corresponding to C =N, C=O, O–C=O, and the (π–π*) shakeup (see Figure S33 for a more detailed view of the smaller components).^6,39^ Comparing the C 1s spectral intensity after annealing with that after UV irradiation, one notes that no carbon was lost in the heat treatment, indirectly confirming that the UV light indeed polymerized the SAM.

One can get a first idea of the thickness of the annealed layer by comparing the intensity ratio of the C 1s and Cu 2p XPS signals with those retrieved from the spectrum of a single layer of CVD-grown graphene on copper foil, where *I*_C 1s_/*I*_Cu 2p_ ∼ 1 (see Figure S34). For the polymerized **1**^**SH**^ SAM, after annealing *I*_C 1s_/*I*_Cu 2p_ > 4 for some samples, the copper signal was no longer visible because it was completely attenuated (Figure S35), thus indicating that there is more than a single layer of carbon on the surface.

By comparing the B 1s spectra in Figure 2 top right panel, (b) and (c), one notes that the spectrum after annealing comprises three additional components: next to the one at 189.6 eV corresponding to C–B–N from the borazine ring, there are also contributions at BE 188.6 eV due to B–C_3_, at 190.7 eV deriving from B–N bonds (similar to h-BN, where B is bound to three N atoms),^6,45,46^ at 192.0 eV attributable to B–O that possibly formed during the transfer of the sample through air.^47^ The B 1s spectrum after annealing (c) suggests that borazine rings remained isolated in the carbon matrix, while some rings decomposed during heat treatment or segregated into small BN domains surrounded by sp^2^-graphitic carbon.^6^

This picture is supported by the N 1s spectrum (Figure 2 bottom left panel, c), which consists of four components: the component at a BE of 397.7 eV arises from N–B, similar to h-BN domains;^6,9^ the component at 398.7 eV stems from C–N–B (nitrogen bonds to both carbon and boron) from the borazine ring.^39^ The last two components at 399.9 and 401.4 eV correspond to N=C and N–C, two bonding structures that commonly remain after the triazole’s thermal decomposition or ring breakdown.^48,49^ The total amount of nitrogen decreased by 2/3 after annealing, and, as already mentioned, S disappeared completely (Figure 2 bottom right panel, c). In other words, the boron to nitrogen ratio changed from 1:3 in the as-deposited SAM to 1:1 after photopolymerization and annealing. Since B and N are still present in isolated borazine rings surrounded by C and some segregated small BN domains, a 1:1 ratio is considered suitable for use in electronic devices^6,9^ and better than what can be obtained by bulk synthesis methods.^9,50^ In summary, from the analysis of the annealed samples, one concludes that there is nitrogen- and boron-codoped carbon on the surface and that this carbon is graphitic in nature.

#### 2.2.2 Film Growth from Thiol borazine **2**^**SH**^

**Figure 3:**
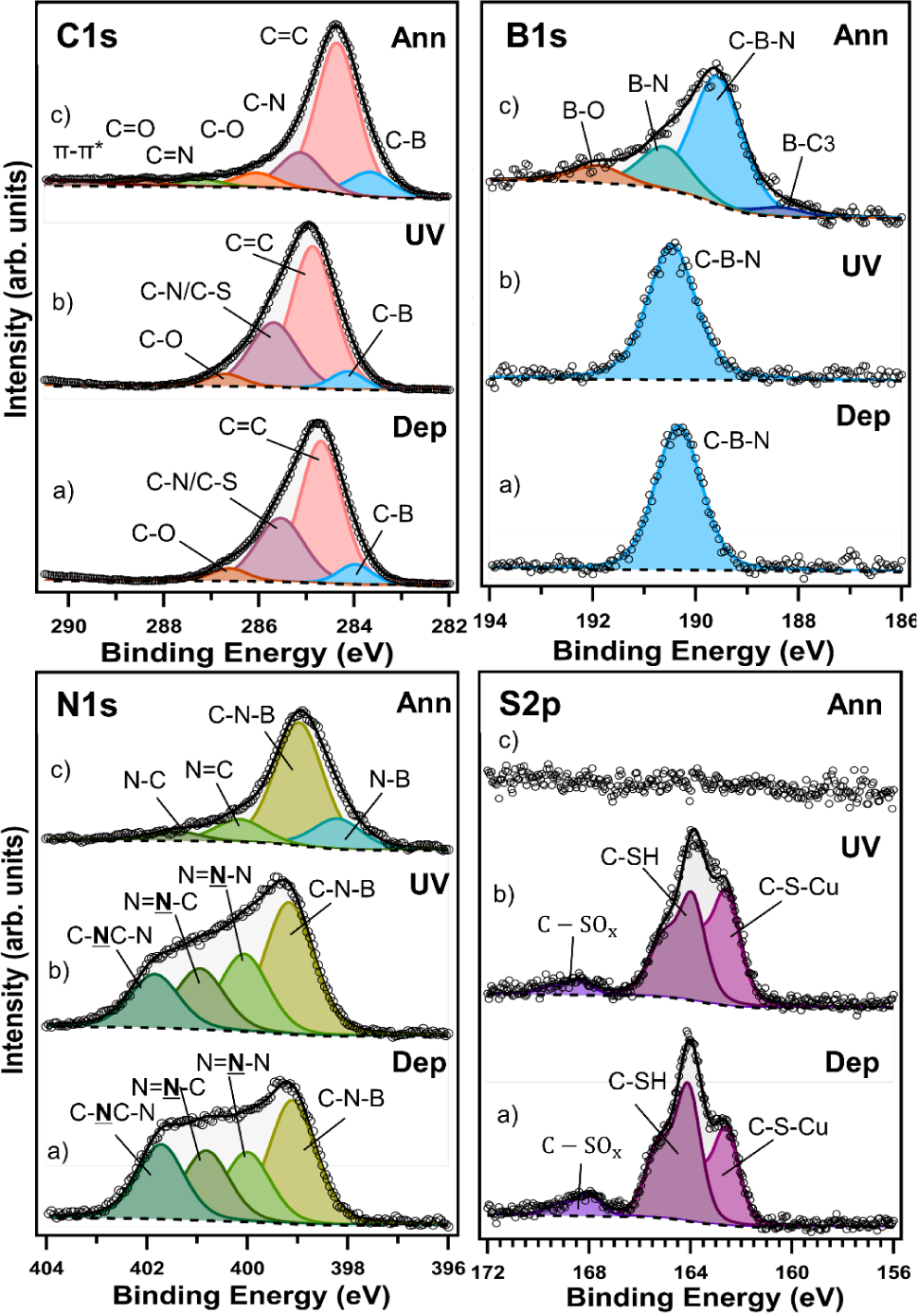
XPS spectra of the C 1s (top left panel), B 1s (top right panel), N 1s (bottom left panel), and S 2p (bottom right panel) core level regions of a self-assembled monolayer of the thiol-terminated borazine derivative **2**^**SH**^ on electropolished copper foil after deposition (a), after UV light-induced polymerization (b) and annealing (c). The corresponding fits are shown as well. For the discussion of the color-coded components, see text.

Borazine molecular precursor **2**^**SH**^ (C_54_H_57_B_3_N_12_S_3_), with a reduced carbon content, was then investigated. As in the previous experiment, we confirmed the molecules’ successful deposition via self-assembly on electropolished copper foil using XPS. Figure 3 shows the C 1s, B 1s, N 1s, and S 2p photoemission lines of **2**^**SH**^ collected after deposition (a), after UV light-induced polymerization (b) and after annealing to transform it into B- and N-codoped graphene (c).

The spectra after the self-assembly of **2**^**SH**^ on electropolished Cu foil were similar to those of **1**^**SH**^, indicating consistent deposition behavior. The main component in the C 1s core level region (Figure 3, top left panel, a) was observed at a BE of 284.6 eV, which corresponds to sp^2^ aromatic carbon–carbon bonds; the additional components at BEs of 283.9 eV and 285.5 eV stem from C–B bonds and C–N/C–S bonds, respectively, while the last contribution at 286.6 eV corresponds to C–O, likely due to surface contamination during sample transfer.^6,7,38,51^ The B 1s photoemission signal for the same as-prepared self-assembled layer (Figure 3 top right panel, a) is peaked at 190.3 eV, as expected for the C–B–N_2_ structure of the borazine core.^6,7^ The absence of additional B 1s components suggests that, also for this system, the central borazine ring remains intact after deposition. The N 1s spectrum (Figure 3 bottom left panel, a) requires again four components for a good fit; the one at a BE of 399.1 eV corresponds to C–N–B_2_ (nitrogen bound to boron in the thiol-borazine ring), while the component at 400.0 eV is due to N=N–N (nitrogen bonding with two nitrogens). Another component at 400.8 eV is ascribed to N=N–C, and the fourth component at 401.7 eV derives from three-coordinated nitrogen (the last three peaks stem from the triazole aryl of the molecule).^6,9,39,40^Figure 3 shows a higher intensity for the first peak (borazine core) than other peaks (triazole components) due to possible X-ray-induced degradation, as discussed for **1**^**SH**^ and reported in the literature.^40^

As for **1**^**SH**^, the S 2p spectrum (Figure 3 bottom right panel, a) showed a component peaked at a BE of 164.0 eV attributed to C–SH (free thiol groups not bound to Cu). Another component peaked at the lower BE of 162.9 eV testifies to the formation of C–S–Cu (copper sulfides) and hence points to the fact that we produce a layer of integer borazine molecules, covalently anchored during the self-assembly. A small component at 168.0 eV attributed to C–SO_*x*_ (oxidized sulfur) was detected, possibly from adsorbed oxygen while introducing the sample in UHV conditions, as it happened with **1**^**SH**^.^41^ The composition of the **2**^**SH**^ SAM was 72.9 at % carbon, 3.9 at % boron, 14.2 at % nitrogen, 4.3 at % sulfur, and 4.7 at % oxygen, which agrees with the stoichiometry of the molecule.

Photopolymerization for 6 h is mirrored by the same changes in the XPS spectra (Figure 3b) as discussed for **1**^**SH**^, namely a shift of the main components of all spectral lines to higher binding energies, pointing to dehydrogenation. The N 1s line (Figure 3 bottom left panel, b) shows a different relative intensity of the components: three-coordinated nitrogen decreased by 6% in favor of the other two components due to the decomposition of the triazole during the UV irradiation and XPS measurements. In the S 2p spectrum (Figure 3 bottom right panel, b), the relative spectral intensity due to copper sulfide increased from 38% of the total S 2p intensity before the UV irradiation to 48% after UV irradiation. This suggests that when hydrogen is lost from C-SH moieties during the UV irradiation, the sulfur binds immediately with Cu. After these light-induced reactions take place, half of the anchoring groups of **2**^**SH**^ are grafted to the surface.

After photopolymerization, the sample was annealed in UHV at 500 °C for 2 h to convert the polymerized molecules into graphitic carbon, aiming for graphene formation at a lower temperature. Unlike in the experiment with **1**^**SH**^, we kept the sample in UHV during heat treatment to avoid contaminants from air exposure. In Figure 3, all core level photoemission lines (c) collected after annealing are shifted by ∼0.4–0.8 eV toward lower binding energies. In the C 1s spectrum, shown in Figure 3 (top left panel, c), the main component is now peaked at a BE of 284.3 eV, a typical value for graphitic carbon (C–C), as also observed for **1**^**SH**^ (vide supra). The smaller contribution at lower BE (283.6 eV) corresponds to C–B. The contributions of C–N, C–O, C=N, and C=O are located at 285.1, 286.0, 287.2, 288.5, and 290.5 eV, respectively. There is the (π–π*) shakeup (for a more detailed view of the small contribution of the peaks, see Figure S33).^6,39,52^ The C 1s line after annealing confirmed indirectly that UV light indeed polymerized the self-assembled layers, but the ratio of the carbon and copper photoemission intensities *I*_C 1s_/*I*_Cu 2p_ (see Figure S35) indicates that there is more than a single layer of carbon on the surface. As seen for **1**^**SH**^, comparing with *I*_C 1s_/*I*_Cu 2p_ of a single layer of CVD-grown graphene on copper foil (Figure S34) leads to the conclusion that also the BN-doped graphene produced from **2**^**SH**^ comprises several layers, even though **2**^**SH**^ has 24 carbon atoms less than **1**^**SH**^.

The B 1s spectrum collected after annealing (Figure 3 top right panel, c) consists of four components. Despite the precautions taken to avoid contamination from air exposure, at the BE of 191.9 eV, there is the spectral fingerprint of B–O bonds.^45^ The second component at 190.6 eV corresponds to B–N bonds as in hBN,^6,45^ the third and most intense one at 189.5 eV is due to C–B–N_2_ from the borazine rings,^39^ and the small fourth component at 188.3 eV stems from B–C_3_.^44^ It is important to note that as discussed for **1**^**SH**^, also the B 1s spectrum of the BN-doped graphene obtained from **2**^**SH**^ indicates that molecules decomposed or segregated BN domains formed during annealing, but the relative intensity of the component due to integer borazine rings is higher when **2**^**SH**^ is used as the precursor molecule. Similar components with higher intensities were observed after annealing the photopolymerized **1**^**SH**^ SAM. Presumably, the higher annealing temperature used in the case of **1**^**SH**^ led to more segregated BN domains and favored molecular decomposition (Figure 2).

The presence of borazine surrounded by sp^2^-graphitic carbon in the film obtained from **2**^**SH**^ is supported by the N 1s spectrum (Figure 3 bottom left panel, c), which consists of four components; the component at a BE of 398.2 eV arises from N–B similar to h-BN domains,^6^ while the component at 398.9 eV stems from C–N–B_2_ (nitrogen bound to boron from the borazine rings).^39^ The other two components at 400.1 and 401.3 eV stem from N=C or pyridinic nitrogen and N–C or graphitic nitrogen (also found in annealed N-doped carbon samples),^47,53^ both presumably the result of the triazole decomposition. The intensity of the N 1s peak decreased by 50% after heat treatment, and sulfur disappeared completely. In other words, the boron to nitrogen ratio changed from 1:3 in the as-deposited SAM of **2**^**SH**^ to 1:1 in the polymerized and heat-treated sample. The composition of this film, as deduced from XPS, was 88.0 at % carbon, 5.8 at % nitrogen, 4.1 at % boron, and 2.1 at % oxygen. This final stoichiometry shows higher atomic percentages of N and B, along with reduced oxygen content compared to the doped graphene derived from **1**^**SH**^ (see Figure S35). A higher BN content in the graphitic carbon, especially when associated with integer borazine rings, significantly enhances the chemical reactivity or charge carrier mobility, making the BNC film obtained from **2**^**SH**^ more promising for further testing and device applications.

### 2.3 Raman Spectroscopy Characterization of the BNC Films

**Figure 4:**
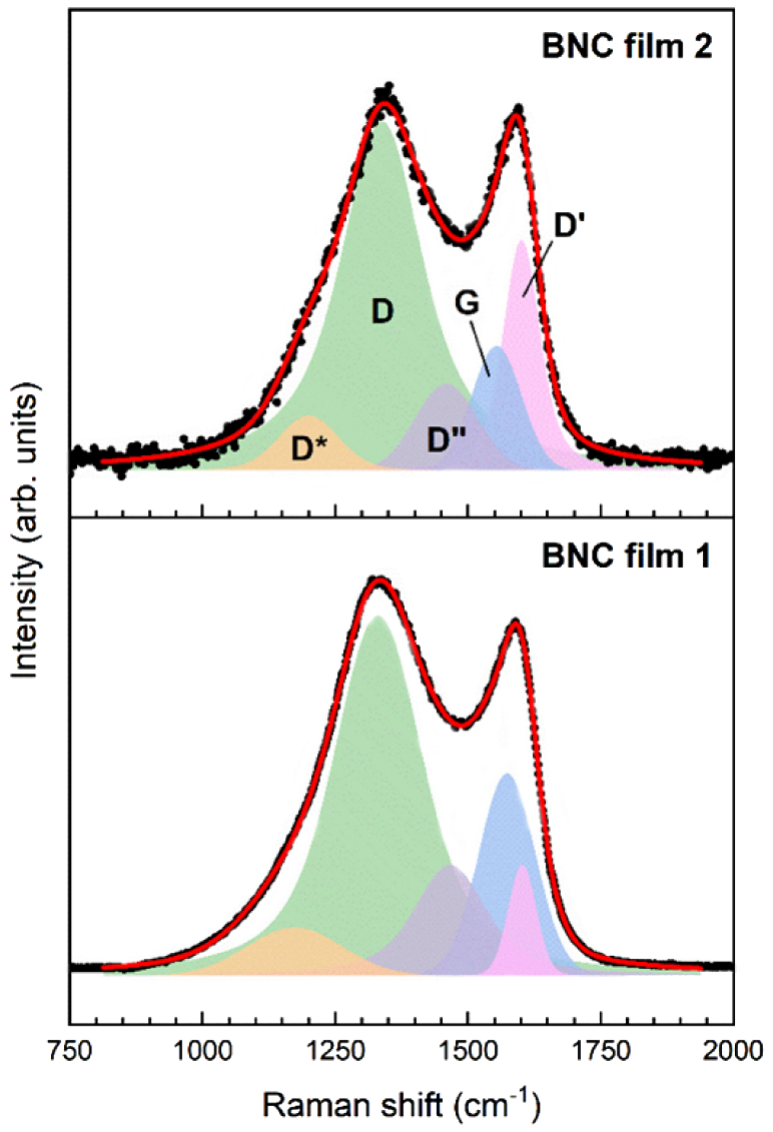
Raman spectra acquired after annealing of the polymerized thiol-borazine SAM on electropolished copper foil. Bottom spectrum corresponds to the film from **1^SH^** and the top spectrum corresponds to the film from **2^SH^**. The excitation wavelength was 632.8 nm.

Raman spectroscopy was employed to examine the effects of incorporating borazine rings into the graphitic backbone of the BNC films. Figure 4 shows the deconvoluted Raman spectra of the films prepared by using thiol borazine precursors **1**^**SH**^ and **2**^**SH**^. The deconvolution was performed by fitting each spectrum with five symmetric Voigt-type peaks corresponding to the D*, D, D″, G, and D′ bands characteristic of graphene-based materials with defects (ref (54), and references therein). The curve-fitting parameters are presented in Table S1, and the calculated intensity ratios, *i.e*., the peak area ratios, are presented in Table S2.

The spectra reveal intense and broad D bands positioned at 1330–1340 cm^–1^, which typically correspond to the presence of structural defects in the planar sp^2^ carbon network of graphene.^55^ It is also worth noting that the D bands of both films overlap with those corresponding to the E_2g_ vibrational mode in hBN at 1366 cm^–1^.^56^ The presence of borazine rings is evidenced by the redshifts of the G band positions of both films relative to those of single-layer graphene, multilayer graphene, and graphite (∼1584 cm^–1^).^57,58^ Similar G band-shaped redshifts originating from doping graphene with heteroatoms have been reported in other works and attributed to the sensitivity of the G band to the charge carrier concentration and to dopants. Furthermore, the redshift of the G band suggests that borazine rings are n-type dopants (ref (59) and references therein).

The D-to-G and D′-to-G band intensity ratios (*I*_D_/*I*_G_ and *I*_D′_/*I*_G_, respectively), which are directly proportional to the defect concentration in graphene-based materials (refs (54)(60) and references therein) are correlated with the level of BN doping in the films. As can be seen in Table S2, *I*_D_/*I*_G_ and *I*_D′_/*I*_G_ of the film prepared from **2**^**SH**^ are higher than those of the film obtained from **1**^**SH**^. This result is corroborated by the stronger redshift of the G band for this sample and agrees with the XPS data, which indicated that more borazine rings are integrated into the graphitic lattice of the film prepared from **2**^**SH**^ than in the one prepared from **1**^**SH**^. However, the *I*_D_/*I*_G_ ratio can also serve to estimate the average in-plane crystallite size (*L*_a_) of the sp^2^ carbon domains in each film using the following equation:^61^
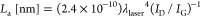
1where λ_laser_ is the laser excitation wavelength in nanometers. For the films prepared from **1**^**SH**^ and **2**^**SH**^, the calculated *L*_a_ values were 10.7 and 5.8 nm, respectively. Following a different approach, the average distance between the in-plane lattice defects (*L*_D_) can be calculated using
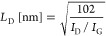
2

For BNC films obtained from **1**^**SH**^ and **2**^**SH**^, the calculated *L*_D_ values were found to be 5.3 and 3.9 nm, respectively. This analysis concludes that the film prepared from **2**^**SH**^ has smaller crystalline domains and a higher defect density than the one fabricated using **1**^**SH**^.

#### 2.3.1 Transmission Electron Microscopy Characterization of the BNC Films

Direct insight into the morphological properties of the codoped graphene films obtained from **1**^**SH**^ and **2**^**SH**^ was gained from transmission electron microscopy (TEM). For this, the BNC films were transferred from the Cu foil to a TEM grid by chemically etching the copper substrate, as detailed in the Experimental Section.

**Figure 5:**
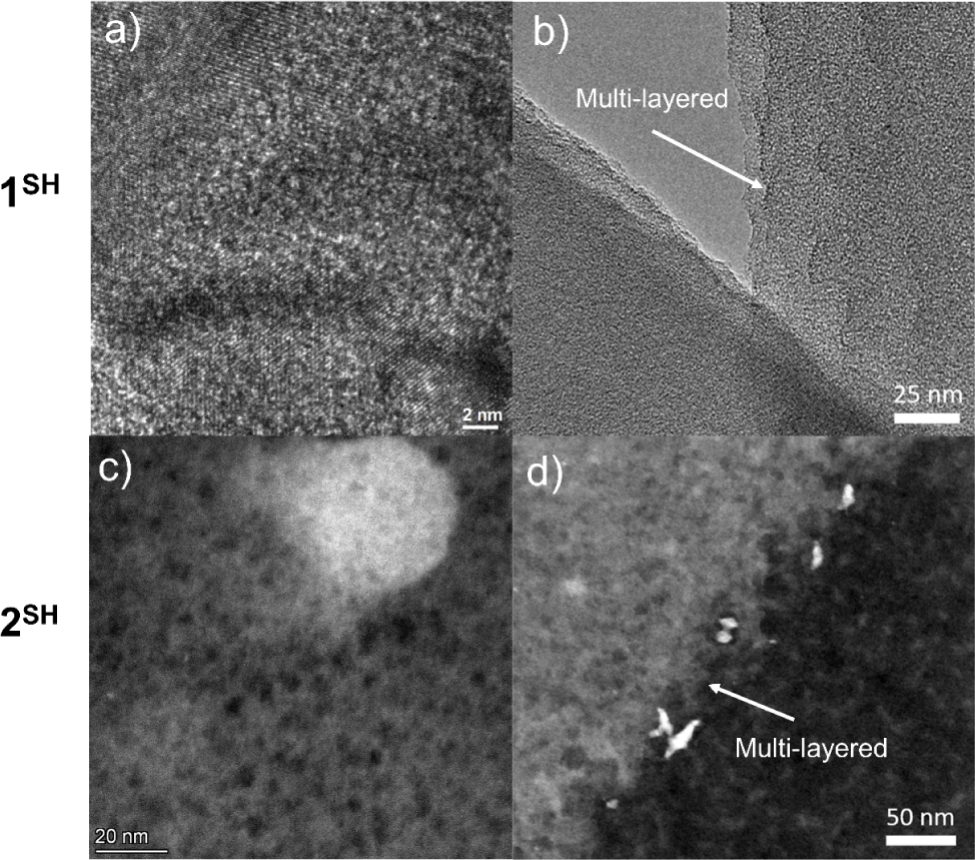
TEM micrographs of codoped graphene samples resulting from annealing a polymerized **1**^**SH**^ SAM (a, b) or a **2**^**SH**^ SAM (c, d) on electropolished copper foil; the images were collected after transfer to a TEM grid.

The TEM images (see Figure S36) demonstrate that both molecular precursors yield continuous films that can be easily transferred to a TEM grid, albeit with some macroscopic folds, as most clearly seen for the film from **2**^**SH**^ and some minor Cu residues. Figure 5 shows the high-angle annular dark field scanning TEM (HAADF STEM) images from the BNC films obtained from **1**^**SH**^ (b) and **2**^**SH**^ (d), both taken at the edge of the respective film. The difference in morphology between the two is striking. While the film obtained from **1**^**SH**^ clearly presents a layered structure that resembles few-layered graphene,^62^ the BNC film from **2**^**SH**^ is much more disordered, and no clear layered structure could be identified. Figure 5a shows the film obtained from **1**^**SH**^ at higher magnification, but no clear crystal structure could be seen, nor was it possible to identify segregated small domains of BN. We attempted to image the film from **2**^**SH**^ at higher magnification but were unsuccessful. Figure 5c hints that this film contains nanopores. The differences in morphology can be attributed to the difference in annealing temperature: 827 °C for the photopolymerized SAM of **1**^**SH**^ and 500 °C for the one obtained from **2**^**SH**^.

Having proven that BNC films can be fabricated and transferred to another substrate, we moved on toward device application by implementing the film prepared from **2**^**SH**^ as a state-of-the-art BN-doped graphene (BNC) film as the CE in a DSSC architecture (see Scheme S2). The reasons for this choice are 2-fold – (i) thiol borazine **2**^**SH**^ comes with simpler synthesis steps along with a higher production yield (15% vs. 1% for **1**^**SH**^), minimizing the challenge of heteroatom-doped graphene compounds yield;^18^ (ii) more importantly, the film produced from **2**^**SH**^ exhibited more intact borazine rings along with less segregation in BN domains, as confirmed by the XPS study.

#### 2.3.2 Electrocatalytic Behavior of the BNC Films

**Figure 6:**
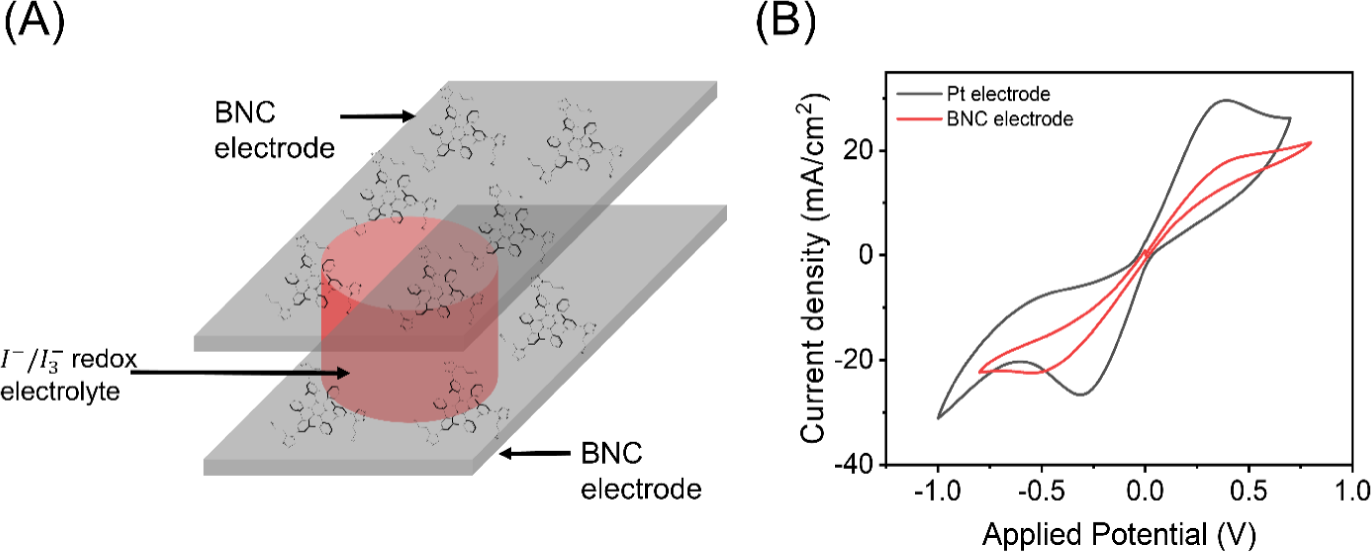
BNC symmetric cell sketch (A) and linear sweep voltammograms of BNC and Pt cells (B).

First, we investigated the electrocatalytic behavior of the BNC films by carrying out linear sweep voltammetry (LSV) in a symmetric cell configuration (FTO/BNC film/tri-iodide electrolyte/BNC film/FTO using FTO/Pt electrodes as reference, see Experimental Section).^63^Figure 6 shows that both BNC and Pt cells feature symmetrical redox behavior with well-defined oxidation and reduction peaks. In detail, BNC cells exhibit a slightly lower cathodic (*J*_PC_) and anodic (*J*_PA_) peak current density (Figure 6B; *J*_PC_ and *J*_PA_ of −22.4 mA/cm^2^ and +18.2 mA/cm^2^ for BNC cells vs. *J*_PC_ and *J*_PA_ of −26.6 mA/cm^2^ and 29.2 mA/cm^2^ for Pt cells). In addition, the peak-to-peak (*E*_PP_) potential for BNC cells was slightly higher than that of Pt cells (0.89 V vs. 0.64 V). Thus, the BNC cells can be considered good enough to perform as CEs in DSSCs (refs (17, 18, 64) and references therein).

#### 2.3.3 DSSC Fabrication and Characterization

**Figure 7:**
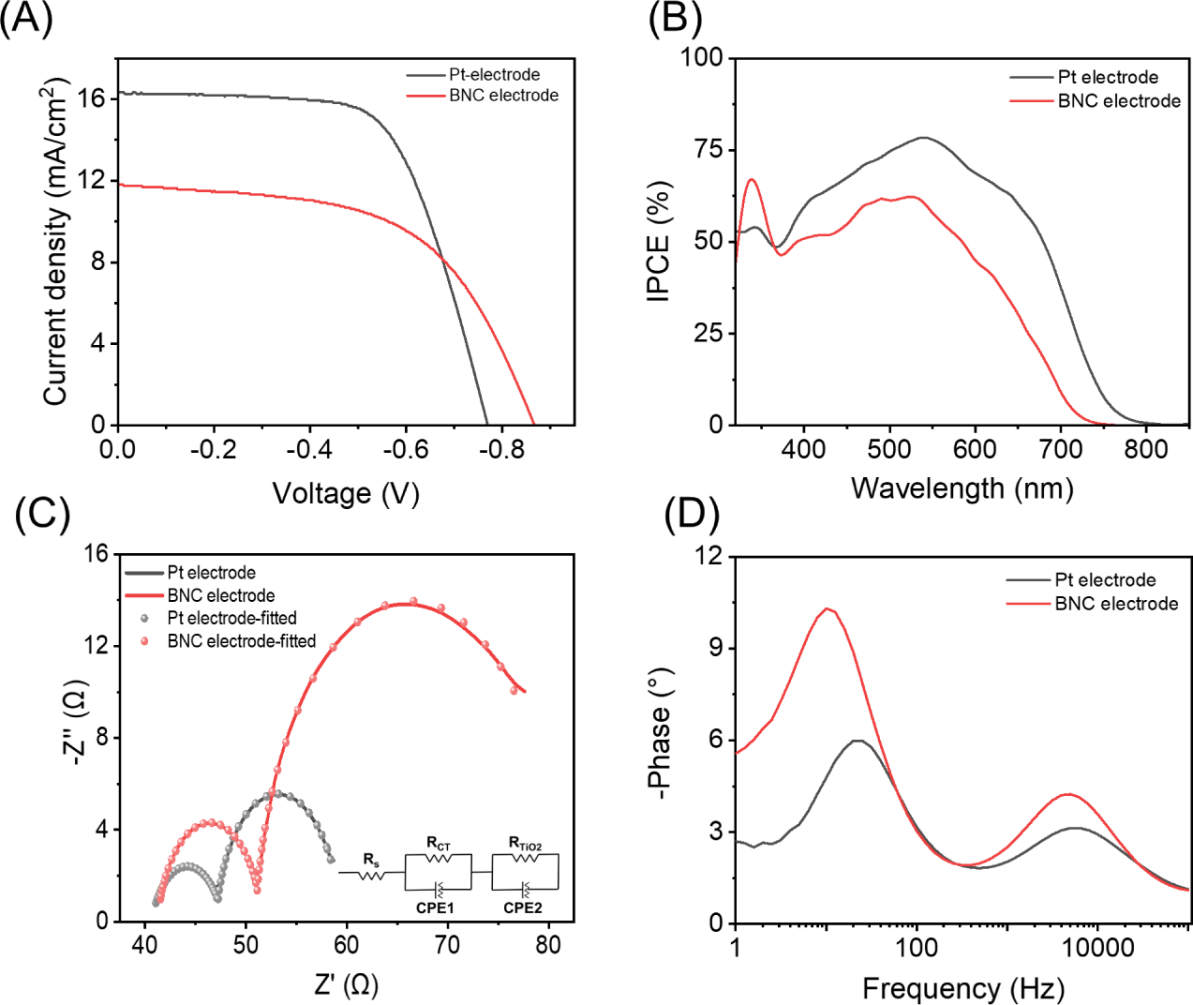
(A) Light *J*–*V* (under standard 1 sun condition), (B) IPCE spectrum, (C) Nyquist plot (the equivalent circuit model applied for fitting is shown in the inset), and (D) Bode phase plot of DSSCs fabricated with BNC and Pt counter electrodes.

After having demonstrated the promising electrocatalytic behavior of BNC electrodes, the next step was their integration as CEs in classical DSSCs with N719-sensitized TiO_2_ photoanodes and a liquid iodine/tri-iodide-based electrolyte (see Experimental Section). At first, the photocurrent–voltage (*J*–*V*) performance was measured under standard 1 sun AM 1.5G conditions. As expected from the electrocatalytic behavior of the above BNC cells, the short-circuit current (*J*_sc_) reached a value of 11.80 mA/cm^2^, which is lower than that of the reference Pt CEs (16.34 mA/cm^2^). This was also confirmed by the incident photon conversion efficiency (IPCE) measurements that showed a lower conversion for the BNC-DSSCs with an overall calculated *J*_sc_ of 9.64 mA/cm^2^ (14.70 mA/cm^2^ for Pt-reference devices); see Figure 7. However, the fill factor (FF) amounted to *ca*. 60% for both Pt- and BNC-DSSCs and was thus not strongly impacted by the type of CE chosen. To understand the effect of the CE on device performance, we carried out an electrochemical impedance spectroscopy (EIS) analysis at 1 sun and open-circuit voltage (*V*_oc_) conditions using the equivalent model shown in Figure 7C.^65^ In detail, the Nyquist plot consists of two semicircles (Figure 7C): a small semicircle associated with the catalytic electrolyte regeneration at the CE interface and a large semicircle related to charge collection at the TiO_2_ electrode. This is confirmed by the Bode phase plots that exhibit two frequency peaks associated with the CE and TiO_2_ electrodes.^66^ Specifically, both BNC and Pt CEs showed similar charge transfer resistances of 10.2 and 7.1 Ω at frequencies of around 3800 and 5,200 Hz – Figure 7D. This value is remarkable, taking into account the difference in electrode thickness compared to the reference Pt nanoclusters – see the Experimental Section. Finally, the resistance associated with the TiO_2_ electrode is significantly higher for BNC-DSSCs than for Pt-DSSCs, as expected from the lower *J*_sc_ values – Figure 7C and Table 1.^64^ Overall, the BNC CEs demonstrate a promising η of up to 6%, close to that of the reference Pt-DSSCs (8%). The device parameter distribution is tabulated in Figure S37. Interestingly, our BNC electrodes provide outstanding η values considering the recent prior art, in which, for example, nitrogen and boron dual-doped porous graphene counter electrodes prepared by one-step laser induction achieved around 5% DSSC efficiency.^27,28^ Although graphene-based counter electrodes have been previously investigated, there are relatively few reports, to the best of our knowledge, on the use of boron- and nitrogen-doped graphene as counter electrodes in DSSCs. A comparison with previous works is provided in Table S3.

**Table 1:** Figures of Merits of DSSCs Fabricated with BNC and Pt CEs.^a^

CE	*J*_SC_ (mA/cm^2^)	*V*_OC_ (mV)	FF (%)	η (%)	*R*_CT_ (Ω)	(Ω)
Pt	16.34 ± 0.62	778 ± 8.38	63 ± 2.5	8.01 ± 0.38	7.1 ± 0.6	12.15 ± 0.74
BNC	11.8 ± 0.47	866 ± 10.91	59 ± 1.9	6.03 ± 0.17	10.2 ± 0.86	31.18 ± 3.75

a The figures of merit of the devices tabulated in the table correspond to the most representing, and the standard error is calculated based on the three independent devices measured under the same conditions.

**Figure 8:**
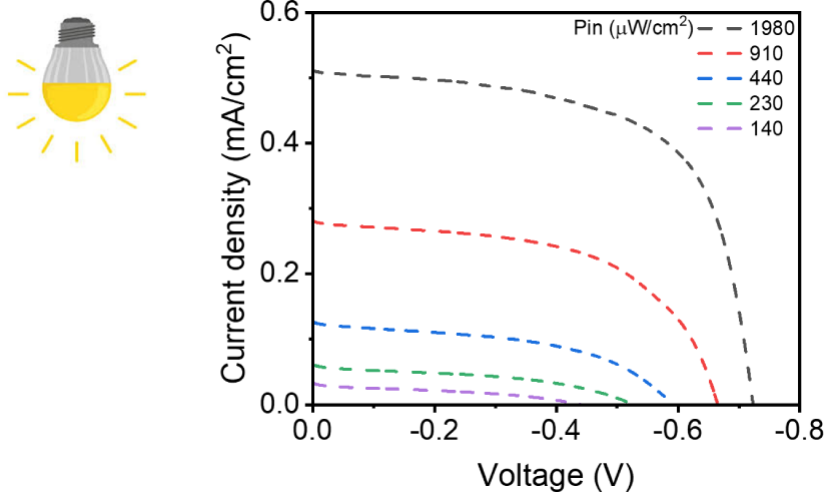
*J*–*V* curves of the DSSC devices fabricated with BNC CEs fabricated from **2**^**SH**^ under indoor ambient condition.

As a final step to further demonstrate the potential of BNC-CEs, we evaluated the performance of BNC-DSSCs under low-intensity ambient light conditions relevant for indoor energy harvesting.^67^ Specifically, we measured the *J*–*V* characteristics of the devices across a range of incident power densities from 140 to 1980 μW/cm^2^, using a commercial white LED (WINGER 3 W 6500 K; see Experimental Section).^68^ Under these dim light conditions, the BNC devices displayed well-defined *J*–*V* curves, achieving a remarkable η of up to 11.8% at *P*_in_ = 1980 μW/cm^2^ (Figure 8 and Table 2). Notably, these devices were able to harvest energy (*P*_out_) within the range of 5–235 μW/cm^2^, highlighting their strong potential for powering indoor household devices and Internet of Things devices.^69^

**Table 2:** Figures of Merit of DSSCs Fabricated with BNC CEs Were Measured under Light Indoor Ambient Conditions.^a^

Input power (μW/cm^2^)	*J*_SC_ (mA/cm^2^)	*V*_OC_ (mV)	FF (%)	η (%)	Output power (μW/cm^2^)
1980	511 ± 11	727 ± 17	63 ± 0.37	11.89 ± 0.54	235 ± 11
910	280 ± 5	668 ± 14	56 ± 0.54	11.51 ± 0.53	104 ± 4
440	126 ± 3	595 ± 16	48 ± 0.66	8.18 ± 0.45	36 ± 2
230	60 ± 2	530 ± 11	43 ± 0.68	5.94 ± 0.30	14 ± 1
140	32 ± 1	444 ± 11	35 ± 0.63	3.55 ± 0.21	5 ± 0.3

a The figures of merit of the devices tabulated in the table correspond to the most representing, and the standard error is calculated based on the three independent devices measured under the same conditions.

## 3 Conclusion

This work demonstrated that two different thiol-bearing borazine derivatives can serve as molecular precursors for synthesizing boron- and nitrogen-codoped graphenoid films. The experimental approach consisted of self-assembling the borazine derivatives on copper foil, followed by photopolymerization with UV light to prevent desorption during the subsequent heat treatment to transform the cross-linked molecules into codoped graphene. The success of the synthesis was demonstrated by X-ray photoelectron spectroscopy, which also revealed key differences between films derived from the two precursors. Films derived from **2**^**SH**^, the precursor with fewer carbon atoms, exhibited a higher proportion of intact borazine rings within the graphene matrix and showed reduced segregation of BN domains. These results were partly attributed to the lower annealing temperature used for **2**^**SH**^, which also largely influenced the morphology, as deduced from Raman and electron microscopy studies. TEM evidenced a few-layer graphene-like structure for the film from **1**^**SH**^ but a more defect-rich architecture for the film from **2**^**SH**^. The electrocatalytic behavior of BNC films prepared from **2**^**SH**^ when employed as CE in DSSCs was judged promising, reaching an η of up to 6% under standard conditions (AM 1.5G, 1 sun illumination) and 11.8% under indoor illumination conditions. Importantly, this BNC-CE effectively operated under low-light conditions, resulting in a significant indoor energy harvesting power of up to 235 μW/cm^2^. Despite the success of this bottom-up self-assembly method to prepare BN-doped electrodes, work is ongoing to control the defect density and doping level better to establish clear correlations between these parameters and the electronic and catalytic performance, which are of paramount relevance for optimizing device behavior. Overall, this work has proven to be a straightforward synthesis strategy for B- and N-doped graphene films that can be employed as effective CEs in DSSCs, offering promising performance under both standard solar and indoor lighting conditions. Hence, a new route toward the milestone of Pt-free and sustainable CEs in DSSCs can be envisaged.

## 4 Experimental Section

### 4.1 Synthesis of Precursors

Experimental details for the synthesis of borazines **1**^**SH**^ and **2**^**SH**^ are found in the Supporting Information.

### 4.2 Synthesis of the BNC Films

Copper foils were used for the deposition of the two molecular precursors. The copper foils (thickness 25 μm, 99.99% purity, Goodfellow and Alfa Aesar) were cleaned by electropolishing before the self-assembly of the molecular precursors. For this, the Cu foil was connected to the positive terminal of the power supply (E0300–0.1-L, Delta Elektronika) and immersed in the electrolyte (with another copper foil as the anode), an aqueous solution (2:1:1) of Milli-Q water, phosphoric acid (85 wt % in H_2_O, Sigma-Aldrich), and acetic acid (purity 99%, Sigma-Aldrich). After a voltage of 2.5 V was applied for 5 min, the surface oxide had dissolved in the electrolyte due to a hydrogen reduction reaction, which took place at the cathode. After electropolishing, the surface was smooth and showed no more traces of oxide, as confirmed by XPS (not shown). The electropolished foils were thoroughly rinsed with Milli-Q water, ethanol (≥99.50%, ACS reagent, Sigma-Aldrich), and chloroform (≥99.0% purity, Lab-Scan) before being immersed in one of the thiol borazine solutions. The self-assembly occurred by immersing the Cu foil in a 0.3 mM solution of **1**^**SH**^ in chloroform for 24 h or in a 0.2 mM solution of **2**^**SH**^ in chloroform for 15 h, both in the dark at room temperature. After self-assembly, the substrates were rinsed with chloroform, thoroughly dried with an argon gas stream (5.0 purity, Linde), and introduced immediately into the UHV system (base pressure ∼9 × 10^–10^ mbar). There, the adsorbed layers were first characterized by X-ray photoelectron spectroscopy, and then polymerization was induced by irradiation with a commercial He–I discharge lamp (VUV-Discharge lamp HIS-13 Omicron Focus, photon energy 21.22 eV, operating at a pressure of ∼10^–8^ mbar). After polymerization, samples of **1**^**SH**^/Cu foil were transferred through the air to a vacuum furnace (EHA 12/150B, Carbolite Gero) operating at a base pressure of ∼10^–5^ mbar, where they were annealed at 827 °C for 2 h to induce the transformation to doped graphene. **2**^**SH**^/Cu foil samples instead never left the UHV chamber, and after polymerization, they were annealed to 500 °C for 2 h (*p* = ∼1 × 10^–9^ mbar) to induce the transformation to doped graphene.

### 4.3 Graphene Growth by Chemical Vapor Deposition

As a reference sample, a single layer of graphene was grown by chemical vapor deposition on Cu foil (thickness 25 μm, 99.99% purity, Goodfellow) in a vacuum furnace (base pressure 10^–5^ mbar). Before transferring to the oven, the copper surface was etched in 0.25 mM sulfuric acid (H_2_SO_4_, Sigma-Aldrich) and rinsed with deionized water (Milli-Q, resistivity 18 GΩ cm) to remove the oxide layer from the surface. The Cu foil was then annealed in a mixture of 0.5 mbar hydrogen (Messer, purity 5.0) and 0.1 mbar argon (Linde, purity 5.0) for 40 min. The graphene was grown by exposing the Cu foil to 0.1 mbar argon, 0.5 mbar hydrogen, and 0.5 mbar methane (Messer, purity 4.0) for 2 min at 907 °C. The sample was subsequently cooled to room temperature in 0.1 mbar argon at a cooling rate of 10 °C/min.^43^

### 4.4 Characterization of the BNC Films

XPS spectra were collected with a Scienta R4000 spectrometer, equipped with a monochromatic Al Kα X-ray source (*h*ν = 1486.6 eV) and a hemispherical electron analyzer, operating at a base pressure of ∼9 × 10^–10^ mbar. The overall experimental resolution was 0.4 eV. XPS spectra analysis was done using the least-squares curve-fitting program WinSpec developed at the LISE, University of Namur, Belgium, and included a Shirley baseline subtraction and a peak deconvolution using a linear combination of Gaussian and Lorentzian functions, taking into account the experimental resolution. The spectra were fitted with a minimum number of peaks, consistent with the structure of the molecules on the surface. The binding energies of isolated peaks are given at ±0.05 eV; when more than one component was needed to reproduce the raw data, the error in the component position was ±0.1 eV. The uncertainties in the intensity determinations were approximately 1%. In this study, all measurements were conducted by using freshly prepared samples. Three SAMs were analyzed for each thiol borazine; spectra of the as-deposited SAM and of the photopolymerized SAM before and after annealing were collected in three distinct spots of each sample to check for reproducibility. Raman spectra in the range of 500–3000 cm^–1^ were collected with an Olympus BX51 microscope, fiber-coupled to an Andor Technology DU416A-LDC-DD camera coupled to a Shamrock 163 spectrograph, and a 500 l/mm grating blazed at 750 nm. A HeNe laser (Thorlabs, random polarization) with a wavelength of 632.8 nm was used; the laser power was 9 mW, and the spot size diameter was estimated at 1.0 μm with a 100× objective and 2 μm with a 50× objective. Each spectrum was the average of 40 scans (0.5 s per scan) collected at a 4 cm^–1^ resolution. Spectra were acquired in five different spots of each sample to check for homogeneity. Transmission electron microscopy (TEM) images for the films from **1**^**SH**^ and **2**^**SH**^ were acquired with a JEOL 2010F TEM and an aberration-corrected Thermo Fisher Scientific Titan Cubed electron microscope, equipped with a field emission gun and operated with an accelerating voltage of 200 and 300 keV, respectively. TEM images in bright-field mode were collected with a Gatan CCD camera.

### 4.5 Transfer of the BNC Films to Other Substrates

For inspection with TEM, the BNC films grown on copper foils were transferred onto Quantifoil2/2 TEM grids (Quantifoil Micro Tools GmbH), consisting of a 100 μm-spaced Au mesh onto which an amorphous carbon membrane of 12 nm thickness has been deposited. This membrane contains circular holes (2 μm diameter) with a spacing of 2 μm (except for the TEM grid Quantifoil for the micrograph in Figure S36b, which had a 200 μm diameter). Free-standing BNC films, free of residues and impurities, were prepared on TEM grids by the following procedure. The Quantifoil2/2 TEM grid was directly placed on the copper foil with the amorphous carbon membrane in contact with codoped graphene. A drop of 2-propanol alcohol (IPA, 99.95% purity, Merck) was deposited onto the codoped graphene. During the evaporation of IPA, the codoped graphene and amorphous carbon membrane attached to one another. The sample was then placed in a 5 mM solution of FeCl_3_ (99.99%, Sigma-Aldrich) in water to etch away the copper foil. The TEM grid with graphene was then thoroughly rinsed three times with ultrapure deionized water (Milli-Q, resistivity 18 GΩ cm), followed by annealing at 130 °C for 5 min in an oven (UNE 200, Memmert) to remove the excess water.

For the electrochemical studies of electrodes and device fabrication purposes, the BNC films obtained from thiol borazine **2**^**SH**^ were transferred from Cu foils to fluorine-doped tin oxide (FTO) glass slides – Pilkington TEC15 (12–15 Ω/sq, 84% total transmittance, 74% direct transmittance, 0.6% haze, 12.5 nm roughness, and 2.2–3.2 mm glass thickness) purchased from XOP glass. Before the transfer, the FTO glasses were cleaned as reported elsewhere. The samples were placed (BNC side facing up) in a container with the Cu etching solution (iron trichloride, hydrochloric acid, Sigma-Aldrich) until all the copper was removed. The copper etching solution was slowly changed by deionized water (Milli-Q) in order to wash away any contamination from the BNC film. When all the etching solution was replaced by water, the FTO glass slide was cautiously placed below the floating film, and the film was transferred onto the FTO glass by reducing the water level in the container and letting the film to land on the new substrate. This process was carried out with great care to avoid trapping bubbles between the FTO glass and the film. The films on the new substrate were dried in air at room temperature for 12 h before being used for device fabrication.

### 4.6 Device Fabrication and Characterization

Ti-nanoxide T/SP paste was purchased from Solaronix; 18NR-AO Active Opaque TiO_2_ paste was purchased from Greatcell Solar Materials. H_2_PtCl_6_, used to fabricate counter electrodes, as well as tetra-butyl ammonium iodide (TBAI), 4-tert-butylpyridine (TBP), iodine (I_2_), sodium iodide (NaI), and acetonitrile (ACN), were purchased from Sigma-Aldrich. FTO glass substrates were cleaned with Derquim soap solution, deionized water, and isopropanol in an ultrasonication bath each for 15 min. Afterward, these substrates were dried gently with nitrogen gas flow and further treated with a UV-ozone cleaner (Model No. 256–220, Jelight Company) for 20 min to eliminate any organic residue from the electrode surface. The cleaned FTO glasses were immersed in a 0.4 mM aqueous TiCl_4_ solution (titanium(IV) chloride solution, 0.09 M in 20% HCl, purchased from Sigma-Aldrich) at 70 °C for 30 min and washed with water and ethanol, followed by blow-drying with nitrogen. The treated electrodes were annealed further at 450 °C for 30 min at a rate of increase of 3.5 °C/min. After cooling down, the first T/SP TiO_2_ paste was doctor-bladed using circular Scotch tape with 0.196 cm^2^ active area on FTO glass and dried at 125 °C for 6 min. Similar steps were followed with the 18NR AO Active Opaque TiO_2_ paste. The electrodes were then sintered at 325 °C for 5 min, 375 °C for 5 min, 450 °C for 15 min, and finally 500 °C for 15 min. After cooling, the electrodes were immersed in a 0.5 mM dye solution. The ruthenium dye N719 (ditetrabutyl ammonium cis-bis(isothiocyanato) bis(2,2^´^-bipyridyl-4,4^´^-dicarboxylato)ruthenium(II)) was prepared in an acetonitrile and *tert*-butanol solution (0.5 mM in ACN:TBA 1:1 v/v). The reference counter electrodes were fabricated with 5 mM H_2_PtCl_6_ in an absolute ethanol solution by drop-casting on FTO glass (26 μL) and annealed at 400 °C for 20 min. The sealing of the device was done in three steps: (i) a thermoplastic polyamide heat-resistant biadhesive tape was placed across the periphery of the N719-soaked TiO_2_ film, (ii) 2–3 drops of iodide/triiodide (I^–^/I_3_^–^) redox electrolyte spread over the TiO_2_ film. The I^–^/I_3_^–^ electrolyte solution was composed of 0.4 M NaI, 0.1 M TBAI, 0.5 M TBP, and 0.05 M I_2_ in an acetonitrile solution, and (iii) the CEs were placed on top of the TiO_2_ films and clamped. The symmetric cells were prepared by assembling two CEs (two platinum and two BNC films deposited on FTO) separately by the thermoplastic polyamide heat-resistant biadhesive tape and sealed with the above iodide/triiodide electrolyte.

The photocurrent–voltage measurements of the DSSC device were carried out with a Solar Simulator, Sol3A Class AAA (450 W xenon lamp; Newport) calibrated with a KG5-filtered silicon reference cell (Newport/Oriel, Model 91150 V). *J*–*V* curves were recorded in the range of 0–1.0 V with a scan rate of 0.1 V/s using a potentiostat (PGSTAT204, Metrohm Autolab) under standard 1 sun illumination. The incident photon-to-current conversion efficiency (IPCE) was measured with a QEPVSI-b (Newport) in the spectral range of 300–900 nm using the Lasing Scan software. The electrocatalytic behavior of the symmetric cells was measured with linear sweep voltammetry in the range of −1.0 V to +0.8 V at a scan rate of 0.1 V/s and using the same potentiostat. Electrochemical impedance spectroscopy (EIS) of the devices was performed under constant AM 1.5G 100 mW/cm^2^ by applying a bias at the open-circuit voltage of each device (*V*_OC_) with an amplitude of 10 mV within the frequency range of 0.1 Hz to 10^5^ Hz. Upon measurement, the data were fitted with an equivalent circuit model (see inset of Nyquist plot in Figure 7C) for a dye-sensitized solar cell. The indoor ambient was simulated with a standard daylight white LED lamp (WINGER 3W, 6500K). The input power density (*P*_in_) was calibrated using an Oriel 91150V reference cell and power meter by increasing the distance of the LED, as reported in the literature.^70^

## Supporting Information Available

Compiled information about experimental details for the synthesis of the precursors, synthetic procedures, spectral data and the characterization of the BN-doped graphene films (PDF)

## References

[ref1] Akinwande D.; Brennan C. J.; Bunch J. S.; Egberts P.; Felts J. R.; Gao H.; Huang R.; Kim J.-S.; Li T.; Li Y.; Liechti K. M.; Lu N.; Park H. S.; Reed E. J.; Wang P.; Yakobson B. I.; Zhang T.; Zhang Y.-W.; Zhou Y.; Zhu Y. A Review on Mechanics and Mechanical Properties of 2D Materials—Graphene and Beyond. Extreme Mech. Lett. 2017, 13, 42–77. 10.1016/j.eml.2017.01.008.

[ref2] Ngidi N. P. D.; Ollengo M. A.; Nyamori V. O. Heteroatom-Doped Graphene and Its Application as a Counter Electrode in Dye-Sensitized Solar Cells. Int. J. Energy Res. 2019, 43 (5), 1702–1734. 10.1002/er.4326.

[ref3] Qu L.; Liu Y.; Baek J.-B.; Dai L. Nitrogen-Doped Graphene as Efficient Metal-Free Electrocatalyst for Oxygen Reduction in Fuel Cells. ACS Nano 2010, 4 (3), 1321–1326. 10.1021/nn901850u. 20155972

[ref4] Selvakumar D.; Murugadoss G.; Alsalme A.; Alkathiri A. M.; Jayavel R. Heteroatom Doped Reduced Graphene Oxide Paper for Large Area Perovskite Solar Cells. Solar Energy 2018, 163, 564–569. 10.1016/j.solener.2018.01.084.

[ref5] Wang H.; Hu Y. H. Graphene as a Counter Electrode Material for Dye-Sensitized Solar Cells. Energy Environ. Sci. 2012, 5 (8), 8182–8188. 10.1039/c2ee21905k.

[ref6] Ci L.; Song L.; Jin C.; Jariwala D.; Wu D.; Li Y.; Srivastava A.; Wang Z. F.; Storr K.; Balicas L.; Liu F.; Ajayan P. M. Atomic Layers of Hybridized Boron Nitride and Graphene Domains. Nat. Mater. 2010, 9 (5), 430–435. 10.1038/nmat2711. 20190771

[ref7] Chang C.-K.; Kataria S.; Kuo C.-C.; Ganguly A.; Wang B.-Y.; Hwang J.-Y.; Huang K.-J.; Yang W.-H.; Wang S.-B.; Chuang C.-H.; Chen M.; Huang C.-I.; Pong W.-F.; Song K.-J.; Chang S.-J.; Guo J.-H.; Tai Y.; Tsujimoto M.; Isoda S.; Chen C.-W.; Chen L.-C.; Chen K.-H. Band Gap Engineering of Chemical Vapor Deposited Graphene by in Situ BN Doping. ACS Nano 2013, 7 (2), 1333–1341. 10.1021/nn3049158. 23273110

[ref8] Zhang C.; Fu L.; Liu N.; Liu M.; Wang Y.; Liu Z. Synthesis of Nitrogen-Doped Graphene Using Embedded Carbon and Nitrogen Sources. Adv. Mater. 2011, 23 (8), 1020–1024. 10.1002/adma.201004110. 21341318

[ref9] Xu J.; Jang S. K.; Lee J.; Song Y. J.; Lee S. The Preparation of BN-Doped Atomic Layer Graphene via Plasma Treatment and Thermal Annealing. J. Phys. Chem. C 2014, 118 (38), 22268–22273. 10.1021/jp504773h.

[ref10] Bonifazi D.; Fasano F.; Lorenzo-Garcia M. M.; Marinelli D.; Oubaha H.; Tasseroul J. Boron–Nitrogen Doped Carbon Scaffolding: Organic Chemistry, Self-Assembly and Materials Applications of Borazine and Its Derivatives. Chem. Commun. 2015, 51 (83), 15222–15236. 10.1039/C5CC06611E. 26411675

[ref11] Caputo L.; Nguyen V.-H.; Charlier J.-C. First-Principles Study of the Structural and Electronic Properties of BN-Ring Doped Graphene. Phys. Rev. Mater. 2022, 6 (11), 114001 10.1103/PhysRevMaterials.6.114001.

[ref12] Otero N.; Pouchan C.; Karamanis P. Quadratic Nonlinear Optical (NLO) Properties of Borazino (B_3_N_3_)-Doped Nanographenes. J. Mater. Chem. C 2017, 5 (32), 8273–8287. 10.1039/C7TC01963G.

[ref13] Dosso J.; Battisti T.; Ward B. D.; Demitri N.; Hughes C. E.; Williams P. A.; Harris K. D. M.; Bonifazi D. Boron–Nitrogen-Doped Nanographenes: A Synthetic Tale from Borazine Precursors. Chem. – A Eur. J. 2020, 26 (29), 6608–6621. 10.1002/chem.201905794. 32023358

[ref14] Fresta E.; Dosso J.; Cabanillas-González J.; Bonifazi D.; Costa R. D. Origin of the Exclusive Ternary Electroluminescent Behavior of BN-Doped Nanographenes in Efficient Single-Component White Light-Emitting Electrochemical Cells. Adv. Funct. Mater. 2020, 30 (33), 1906830 10.1002/adfm.201906830.

[ref15] Burton O. J.; Winter Z.; Watanabe K.; Taniguchi T.; Beschoten B.; Stampfer C.; Hofmann S. Putting High-Index Cu on the Map for High-Yield, Dry-Transferred CVD Graphene. ACS Nano 2023, 17 (2), 1229–1238. 10.1021/acsnano.2c09253. 36594782 PMC9878973

[ref16] Lee S. J.; Theerthagiri J.; Nithyadharseni P.; Arunachalam P.; Balaji D.; Madan Kumar A.; Madhavan J.; Mittal V.; Choi M. Y. Heteroatom-Doped Graphene-Based Materials for Sustainable Energy Applications: A Review. Renewable Sustainable Energy Rev. 2021, 143, 110849 10.1016/j.rser.2021.110849.

[ref17] Chen Y.; Gong X.-L.; Gai J.-G. Progress and Challenges in Transfer of Large-Area Graphene Films. Adv. Sci. 2016, 3 (8), 1500343 10.1002/advs.201500343. PMC5067701 27812479

[ref18] Liu F.; Li P.; An H.; Peng P.; McLean B.; Ding F. Achievements and Challenges of Graphene Chemical Vapor Deposition Growth. Adv. Funct. Mater. 2022, 32 (42), 2203191 10.1002/adfm.202203191.

[ref19] Chandrasekhar P. S.; Dubey A.; Qiao Q. High Efficiency Perovskite Solar Cells Using Nitrogen-Doped Graphene/ZnO Nanorod Composite as an Electron Transport Layer. Solar Energy 2020, 197, 78–83. 10.1016/j.solener.2019.12.062.

[ref20] Fang H.; Yu C.; Ma T.; Qiu J. Boron-Doped Graphene as a High-Efficiency Counter Electrode for Dye-Sensitized Solar Cells. Chem. Commun. 2014, 50 (25), 3328–3330. 10.1039/c3cc48258h. 24535331

[ref21] Lin C.-A.; Lee C.-P.; Ho S.-T.; Wei T.-C.; Chi Y.-W.; Huang K. P.; He J.-H. Nitrogen-Doped Graphene/Platinum Counter Electrodes for Dye-Sensitized Solar Cells. ACS Photonics 2014, 1 (12), 1264–1269. 10.1021/ph500219r.

[ref22] Tabassum H.; Zou R.; Mahmood A.; Liang Z.; Guo S. A Catalyst-Free Synthesis of B, N Co-Doped Graphene Nanostructures with Tunable Dimensions as Highly Efficient Metal Free Dual Electrocatalysts. J. Mater. Chem. A 2016, 4 (42), 16469–16475. 10.1039/C6TA07214C.

[ref23] Imamura G.; Chang C. W.; Nabae Y.; Kakimoto M.; Miyata S.; Saiki K. Electronic Structure and Graphenization of Hexaphenylborazine. J. Phys. Chem. C 2012, 116 (30), 16305–16310. 10.1021/jp304879s.

[ref24] Kim I. T.; Song M. J.; Kim Y. B.; Shin M. W. Microwave-Hydrothermal Synthesis of Boron/Nitrogen Co-Doped Graphene as an Efficient Metal-Free Electrocatalyst for Oxygen Reduction Reaction. Int. J. Hydrogen Energy 2016, 41 (47), 22026–22033. 10.1016/j.ijhydene.2016.08.069.

[ref25] Marchionni D.; Basak S.; Khodadadi A. N.; Marrocchi A.; Vaccaro L. Synthesis and Applications of Organic Borazine Materials. Adv. Funct. Mater. 2023, 33 (49), 2303635 10.1002/adfm.202303635.

[ref26] Turchanin A. Graphene Growth by Conversion of Aromatic Self-Assembled Monolayers. Annalen der Phys. 2017, 529 (11), 1700168 10.1002/andp.201700168.

[ref27] Yang B.; Guan Y.; Lu Y.; Chu Z. Laser-Induced Nitrogen and Boron Co-Doped Graphene Film for Dye-Sensitized Solar Cell Applications. Mater. Lett. 2024, 366, 136505 10.1016/j.matlet.2024.136505.

[ref28] Lin K.-Y.; Nguyen M. T.; Waki K.; Jiang J.-C. Boron and Nitrogen Co-Doped Graphene Used As Counter Electrode for Iodine Reduction in Dye-Sensitized Solar Cells. J. Phys. Chem. C 2018, 122 (46), 26385–26392. 10.1021/acs.jpcc.8b06956.

[ref29] Dhillon K. K.; Patyal M.; Bhogal S.; Gupta N. A Review on Advanced Counter Electrode Materials for High-Efficiency Dye Sensitized Solar Cells. J. Coord. Chem. 2024, 77 (17–19), 1933–1968. 10.1080/00958972.2024.2404070.

[ref30] Dhonde M.; Sahu K.; Das M.; Yadav A.; Ghosh P.; Murty V. V. S. Review—Recent Advancements in Dye-Sensitized Solar Cells; From Photoelectrode to Counter Electrode. J. Electrochem. Soc. 2022, 169 (6), 066507 10.1149/1945-7111/ac741f.

[ref31] Prajapat K.; Dhonde M.; Sahu K.; Bhojane P.; Murty V.; Shirage P. M. The Evolution of Organic Materials for Efficient Dye-Sensitized Solar Cells. J. Photochem. Photobiol., C 2023, 55, 100586 10.1016/j.jphotochemrev.2023.100586.

[ref32] Prajapat K.; Mahajan U.; Kumar A.; Dhonde M.; Sahu K.; Vyas S.; Riyad Y. M.; El-Bahy Z. M. Next-Generation Counter Electrodes for Dye-Sensitized Solar Cells: A Comprehensive Overview. Sustainable Mater. Technol. 2024, 42, e01178 10.1016/j.susmat.2024.e01178.

[ref33] Rocard L.; Berezin A.; De Leo F.; Bonifazi D. Templated Chromophore Assembly by Dynamic Covalent Bonds. Angew. Chem. Int. Ed. 2015, 54 (52), 15739–15743. 10.1002/anie.201507186. 26637106

[ref34] Kervyn S.; Fenwick O.; Stasio F. D.; Shin Y. S.; Wouters J.; Accorsi G.; Osella S.; Beljonne D.; Cacialli F.; Bonifazi D. Polymorphism, Fluorescence, and Optoelectronic Properties of a Borazine Derivative. Chem. – A Eur. J. 2013, 19 (24), 7771–7779. 10.1002/chem.201204598. 23616404

[ref35] Kalashnyk N.; Nagaswaran P. G.; Kervyn S.; Riello M.; Moreton B.; Jones T. S.; De Vita A.; Bonifazi D.; Costantini G. Self-Assembly of Decoupled Borazines on Metal Surfaces: The Role of the Peripheral Groups. Chem. – A Eur. J. 2014, 20 (37), 11856–11862. 10.1002/chem.201402839. PMC4449113 25079120

[ref36] Schwarz M.; Garnica M.; Fasano F.; Demitri N.; Bonifazi D.; Auwärter W. BN-Patterning of Metallic Substrates through Metal Coordination of Decoupled Borazines. Chem. – A Eur. J. 2018, 24 (38), 9565–9571. 10.1002/chem.201800849. 29701892

[ref37] Marinelli D.; Fasano F.; Najjari B.; Demitri N.; Bonifazi D. Borazino-Doped Polyphenylenes. J. Am. Chem. Soc. 2017, 139 (15), 5503–5519. 10.1021/jacs.7b01477. 28276248

[ref38] Herrera-Reinoza N.; dos Santos A. C.; de Lima L. H.; Landers R.; de Siervo A. Atomically Precise Bottom-Up Synthesis of h-BNC: Graphene Doped with h-BN Nanoclusters. Chem. Mater. 2021, 33 (8), 2871–2882. 10.1021/acs.chemmater.1c00081.

[ref39] Belser A.; Greulich K.; Grüninger P.; Bettinger H. F.; Peisert H.; Chassé T. Visualization of the Borazine Core of B_3_N_3_-Doped Nanographene by STM. ACS Appl. Mater. Interfaces 2020, 12 (16), 19218–19225. 10.1021/acsami.0c02324. 32223213

[ref40] Saad A.; Vard C.; Abderrabba M.; Chehimi M. M. Triazole/Triazine-Functionalized Mesoporous Silica As a Hybrid Material Support for Palladium Nanocatalyst. Langmuir 2017, 33 (28), 7137–7146. 10.1021/acs.langmuir.7b01247. 28635285

[ref41] Turchanin A.; Käfer D.; El-Desawy M.; Wöll C.; Witte G.; Gölzhäuser A. Molecular Mechanisms of Electron-Induced Cross-Linking in Aromatic SAMs. Langmuir 2009, 25 (13), 7342–7352. 10.1021/la803538z. 19485375

[ref42] Zehra T.; Syari’ati A.; Ivashenko O.; Bignardi L.; Van Dorp W. F.; De Hosson J. T. M.; Rudolf P. Graphene Growth from Photo-Polymerized Bi-Phenylthiol Self-Assembled Monolayers. Front. Nanotechnol. 2024, 6, 1366542 10.3389/fnano.2024.1366542.

[ref43] Bignardi L.; Dorp W. F. V.; Gottardi S.; Ivashenko O.; Dudin P.; Barinov A.; Hosson J. T. M. D.; Stöhr M.; Rudolf P. Microscopic Characterisation of Suspended Graphene Grown by Chemical Vapour Deposition. Nanoscale 2013, 5 (19), 9057–9061. 10.1039/C3NR02386A. 23945527

[ref44] Susi T.; Pichler T.; Ayala P. X-Ray Photoelectron Spectroscopy of Graphitic Carbon Nanomaterials Doped with Heteroatoms. Beilstein J. Nanotechnol. 2015, 6 (1), 177–192. 10.3762/bjnano.6.17. 25671162 PMC4311644

[ref45] Mo Z.-J.; Hao Z.-H.; Ping X.-J.; Kong L.-N.; Yang H.; Cheng J.-L.; Zhang J.-K.; Jin Y.-H.; Li L. Synthesized Few-Layers Hexagonal Boron Nitride Nanosheets*. Chinese Phys. B 2018, 27 (1), 016102 10.1088/1674-1056/27/1/016102.

[ref46] Miyamoto Y.; Rubio A.; Cohen M. L.; Louie S. G. Chiral Tubules of Hexagonal. Phys. Rev. B 1994, 50 (7), 4976–4979. 10.1103/PhysRevB.50.4976. 9976827

[ref47] Matsoso B. J.; Ranganathan K.; Mutuma B. K.; Lerotholi T.; Jones G.; Coville N. J. Synthesis and Characterization of Boron Carbon Oxynitride Films with Tunable Composition Using Methane, Boric Acid and Ammonia. New J. Chem. 2017, 41 (17), 9497–9504. 10.1039/C7NJ01886J.

[ref48] Shao Y.; Zhang S.; Engelhard M. H.; Li G.; Shao G.; Wang Y.; Liu J.; Aksay I. A.; Lin Y. Nitrogen-Doped Graphene and Its Electrochemical Applications. J. Mater. Chem. 2010, 20 (35), 7491 10.1039/c0jm00782j.

[ref49] Wang Y.; Shao Y.; Matson D. W.; Li J.; Lin Y. Nitrogen-Doped Graphene and Its Application in Electrochemical Biosensing. ACS Nano 2010, 4 (4), 1790–1798. 10.1063/1.4870424. 20373745

[ref50] Tai J.; Hu J.; Chen Z.; Lu H. Two-Step Synthesis of Boron and Nitrogen Co-Doped Graphene as a Synergistically Enhanced Catalyst for the Oxygen Reduction Reaction. RSC Adv. 2014, 4 (106), 61437–61443. 10.1039/C4RA10162F.

[ref51] Avila J.; Razado I.; Lorcy S.; Fleurier R.; Pichonat E.; Vignaud D.; Wallart X.; Asensio M. C. Exploring Electronic Structure of One-Atom Thick Polycrystalline Graphene Films: A Nano Angle Resolved Photoemission Study. Sci. Rep. 2013, 3 (1), 2439 10.1038/srep02439. 23942471 PMC3743056

[ref52] Bepete G.; Voiry D.; Chhowalla M.; Chiguvare Z.; Coville N. J. Incorporation of Small BN Domains in Graphene during CVD Using Methane, Boric Acid and Nitrogen Gas. Nanoscale 2013, 5 (14), 6552–6557. 10.1039/C3NR01699D. 23759928

[ref53] Panchakarla L. S.; Govindaraj A.; Rao C. N. R. Nitrogen- and Boron-Doped Double-Walled Carbon Nanotubes. ACS Nano 2007, 1 (5), 494–500. 10.1021/nn700230n. 19206671

[ref54] Zygouri P.; Tsiodoulos G.; Angelidou M.; Papanikolaou E.; Athinodorou A.-M. V.; Simos Y.; Spyrou K.; Subrati M.; Kouloumpis A.; Kaloudi A. S.; Asimakopoulos G.; Tsamis K.; Peschos D.; Vezyraki P.; Ragos V.; Gournis D. P. Graphene Oxide and Oxidized Carbon Nanodiscs as Biomedical Scaffolds for the Targeted Delivery of Quercetin to Cancer Cells. Nanoscale Adv. 2024, 6 (11), 2860–2874. 10.1039/D3NA00966A. 38817436 PMC11134231

[ref55] Madito M. J. Correlation of the Graphene Fermi-Level Shift and the Enhanced Electrochemical Performance of Graphene-Manganese Phosphate for Hybrid Supercapacitors: Raman Spectroscopy Analysis. ACS Appl. Mater. Interfaces 2021, 13 (31), 37014–37026. 10.1021/acsami.1c07104. 34318656

[ref56] Purdie D. G.; Pugno N. M.; Taniguchi T.; Watanabe K.; Ferrari A. C.; Lombardo A. Cleaning Interfaces in Layered Materials Heterostructures. Nat. Commun. 2018, 9 (1), 5387 10.1038/s41467-018-07558-3. 30568160 PMC6300598

[ref57] Kaniyoor A.; Ramaprabhu S. A Raman Spectroscopic Investigation of Graphite Oxide Derived Graphene. AIP Adv. 2012, 2 (3), 032183 10.1063/1.4756995.

[ref58] Graziotto L.; Macheda F.; Venanzi T.; Marchese G.; Sotgiu S.; Ouaj T.; Stellino E.; Fasolato C.; Postorino P.; Metzelaars M.; Kögerler P.; Beschoten B.; Calandra M.; Ortolani M.; Stampfer C.; Mauri F.; Baldassarre L. Infrared Resonance Raman of Bilayer Graphene: Signatures of Massive Fermions and Band Structure on the 2D Peak. Nano Lett. 2024, 24 (6), 1867–1873. 10.1021/acs.nanolett.3c03502. 38306119

[ref59] Yadav R.; Joshi P.; Hara M.; Yana T.; Hashimoto S.; Yoshimura M. Intercorrelation between Physical and Electrochemical Behavior of Nitrogen-Doping in Graphene for Symmetric Supercapacitor Electrode. SN Appl. Sci. 2020, 2 (10), 1630 10.1007/s42452-020-03401-x.

[ref60] Kaloudi A. S.; Zygouri P.; Spyrou K.; Athinodorou A.-M.; Papanikolaou E.; Subrati M.; Moschovas D.; Datta K. K. R.; Sideratou Z.; Avgeropoulos A.; Simos Y. V.; Tsamis K. I.; Peschos D.; Yentekakis I. V.; Gournis D. P. A Strategic Synthesis of Orange Waste-Derived Porous Carbon via a Freeze-Drying Method: Morphological Characterization and Cytocompatibility Evaluation. Molecules 2024, 29 (16), 3967 10.3390/molecules29163967. 39203045 PMC11357121

[ref61] Subrati A.; Gurzęda B.; Jeżowski P.; Kościński M.; Nowaczyk G.; Kempiński M.; Florczak P.; Peplińska B.; Jarek M.; Al Wahedi Y.; Kempiński W.; Smardz L.; Krawczyk P. The Impact of Oxygen-Clustering on the Transformation of Electrochemically-Derived Graphite Oxide Framework. Carbon 2024, 217, 118641 10.1016/j.carbon.2023.118641.

[ref62] Canton-Vitoria R.; Alsaleh A. Z.; Rotas G.; Nakanishi Y.; Shinohara H.; Souza F. D.; Tagmatarchis N. Graphene Performs the Role of an Electron Donor in Covalently Interfaced Porphyrin-Boron Azadipyrromethene Dyads and Manages Photoinduced Charge-Transfer Processes. Nanoscale 2022, 14 (40), 15060–15072. 10.1039/D2NR03740H. 36200654

[ref63] Martínez-Muíño A.; Rana M.; Vilatela J. J.; Costa R. D. Origin of the Electrocatalytic Activity in Carbon Nanotube Fiber Counter-Electrodes for Solar-Energy Conversion. Nanoscale Adv. 2020, 2 (10), 4400–4409. 10.1039/D0NA00492H. 36132932 PMC9417869

[ref64] Monreal-Bernal A.; Vilatela J. J.; Costa R. D. CNT Fibres as Dual Counter-Electrode/Current-Collector in Highly Efficient and Stable Dye-Sensitized Solar Cells. Carbon 2019, 141, 488–496. 10.1016/j.carbon.2018.09.090.

[ref65] Sarker S.; Ahammad A. J. S.; Seo H. W.; Kim D. M. Electrochemical Impedance Spectra of Dye-Sensitized Solar Cells: Fundamentals and Spreadsheet Calculation. Int. J. Photoenergy 2014, 2014 (1), 851705 10.1155/2014/851705.

[ref66] Hung I.-M.; Bhattacharjee R. Effect of Photoanode Design on the Photoelectrochemical Performance of Dye-Sensitized Solar Cells Based on SnO_2_ Nanocomposite. Energies 2016, 9 (8), 641 10.3390/en9080641.

[ref67] Margraf J. T.; Lodermeyer F.; Strauss V.; Haines P.; Walter J.; Peukert W.; Costa R. D.; Clark T.; Guldi D. M. Using Carbon Nanodots as Inexpensive and Environmentally Friendly Sensitizers in Mesoscopic Solar Cells. Nanoscale Horiz. 2016, 1 (3), 220–226. 10.1039/C6NH00010J. 32260624

[ref68] Tingare Y. S.; Vinh N. S.; Chou H.-H.; Liu Y.-C.; Long Y.-S.; Wu T.-C.; Wei T.-C.; Yeh C.-Y. New Acetylene-Bridged 9,10-Conjugated Anthracene Sensitizers: Application in Outdoor and Indoor Dye-Sensitized Solar Cells. Adv. Energy Mater. 2017, 7 (18), 1700032 10.1002/aenm.201700032.

[ref69] Pecunia V.; Occhipinti L. G.; Hoye R. L. Z. Emerging Indoor Photovoltaic Technologies for Sustainable Internet of Things. Adv. Energy Mater. 2021, 11 (29), 2100698 10.1002/aenm.202100698.

[ref70] Hora C.; Santos F.; Sales M. G. F.; Ivanou D.; Mendes A. Dye-Sensitized Solar Cells for Efficient Solar and Artificial Light Conversion. ACS Sustainable Chem. Eng. 2019, 7 (15), 13464–13470. 10.1021/acssuschemeng.9b02990.

