# Supplementary material for: Bottom-Up Fabrication of BN-Doped Graphene Electrodes from Thiol-Terminated Borazine Molecules Working in Solar Cells
Source: ACS Appl Mater Interfaces. 2025 Apr 2;17(15):23062–75. doi: 10.1021/acsami.4c23116 (PMC12012745; doi:10.1021/acsami.4c23116)
Supplement: Supplementary file 1 — am4c23116_si_001.pdf [file am4c23116_si_001.pdf]

Supporting Information

Bottom-up fabrication of BN-doped graphene electrodes from thiol-terminated borazine molecules working in solar cells

Carolina M. Ibarra-Barreno [a♦], Sanchari Chowdhury [b♦], Martina Crosta [c♦], Tashfeen Zehra [a], Francesco Fasano [d], Paromita Kundu [e†], Jenthe Verstraelen [e], Sara Bals [e], Mohammed Subrati [f], Davide Bonifazi [c], Rubén D. Costa [b*], and Petra Rudolf [a*]*

[a] Zernike Institute for Advanced Materials, University of Groningen, Nijenborgh 3, 9747 AG, Groningen, The Netherlands.

[b] Technical University of Munich, Campus Straubing for Biotechnology and Sustainability, Chair of Biogenic Functional Materials, Schulgasse 20, 94315 Straubing, Germany.

[c] Institute of Organic Chemistry, Faculty of Chemistry, University of Vienna, Währinger Strasse 38, 1090 Vienna, Austria.

[d] School of Chemistry, Cardiff University, Park Place, Main Building, Cardiff CF10 3AT, United Kingdom.

[e] EMAT, University of Antwerp, Faculty of Science / Department of Physics Campus Groenenborger U407, Groenenborgerlaan 171, 2020 Antwerp, Belgium.

[f] Institute of Nanoscience and Nanotechnology, National Center for Scientific Research 'Demokritos', 15310 Agia Paraskevi, Attica, Greece.

*Corresponding authors: p.rudolf@rug.nl; ruben.costa@tum.de; davide.bonifazi@univie.ac.at

Table of contents

S1 Synthesis of precursors: General remarks 3

S1.1 Instrumentation 3

S1.2 Materials and methods 4

S2 Synthetic procedures and spectral data 5

S2.1 Synthesis of 4-(phenylethynyl)aniline 1^{An} 5

S2.2 Synthesis of ((4-bromo-3,5-dimethylphenyl)ethynyl)trimethylsilane 3 5

S2.3 Synthesis of *N*,*N*',*N*''-tri(4-phenylethynyl)phenyl-*B*,*B*',*B*''-tri(2,6-dimethyl-4-((trimethylsilyl)ethynyl)phenyl)borazine 1^{TMS} 6

S2.4 Synthesis of *N*,*N*',*N*''-tri(4-phenylethynyl)phenyl-*B*,*B*',*B*''-tri(2,6-dimethyl-4-ethynylphenyl)borazine 1^{yne} 7

S2.5 Synthesis of 2-(tritylthio)ethanol 5 7

S2.6 Synthesis of (2-azidoethyl)(trityl)sulfane 4 8

S2.7 Synthesis of *N*,*N*',*N*''-tri(4-phenylethynyl)phenyl-*B*,*B*',*B*''-tri(2,6-dimethylphenyl-4-(1-(2-(tritylthio)ethyl)-1,2,3-triazol-5-yl))borazine 1^{STr} 9

S2.8 Synthesis of *N*,*N*',*N*''-tri(4-phenylethynyl)phenyl-*B*,*B*',*B*''-tri(2,6-dimethylphenyl-4-(1-(2-thioethyl)-1,2,3-triazol-5-yl))borazine 1^{SH} 10

S2.9 Synthesis of *N*,*N*',*N*''-triphenyl-*B*,*B*',*B*''-tri(2,6-dimethyl-4-((trimethylsilyl)ethynyl)phenyl)borazine 2^{TMS} 10

S2.10 Synthesis of *N*,*N*',*N*''-triphenyl-*B*,*B*',*B*''-tri(2,6-dimethyl-4-ethynylphenyl)borazine 2^{yne} 11

S2.11 Synthesis of *N*,*N*',*N*''-triphenyl-*B*,*B*',*B*''-tri(2,6-dimethylphenyl-4-(1-(2-(tritylthio)ethyl)-1,2,3-triazol-5-yl))borazine 2^{STr} 12

S2.12 Synthesis of *N*,*N*',*N*''-triphenyl-*B*,*B*',*B*''-tri(2,6-dimethylphenyl-4-(1-(2-thioethyl)-1,2,3-triazol-5-yl))borazine 2^{SH} 13

S3 NMR spectra 14

S3.1 Characterization of 1^{An} 14

S3.2 Characterization of 3 15

S3.3 Characterization of 1^{TMS} 16

S3.4 Characterization of 1^{yne} 18

S3.5 Characterization of 5 19

S3.6 Characterization of 4 20

S3.7 Characterization of 1^{STr} 21

S3.8 Characterization of 1^{SH} 23

S3.9 Characterization of 2^{TMS} 24

S3.10 Characterization of 2^{yne} 26

S3.11 Characterization of 2^{STr} 27

S3.12 Characterization of 2^{SH} 29

S4 Characterization of the BN-doped graphene films – extra material 31

S5 References 38

S1 Synthesis of precursors: General remarks

S1.1 Instrumentation

Thin layer chromatography (TLC) was conducted on pre-coated aluminium sheets with 0.20 mm Machevery-Nagel Alugam SIL G/UV254 with fluorescent indicator UV254 or 0.20 mm Merck Millipore Silica gel 60 with fluorescent indicator F254.

Column chromatography was carried out using Merck Gerduran silica gel 60 (particle size 40-63 and 63-200 μm).

Melting points (mp) were measured on a Büchi Melting Point B-545 (T_{max} = 300 °C) or a DigiMelt MPA 160 (T_{max} = 260 °C) in open capillary tubes, under air, and are uncorrected. According to the limitations of the apparatus, the compounds that did not melt or decompose (dec) up to 300 or 260 °C are presented as "> 300 °C" or "> 260 °C".

Nuclear magnetic resonance (NMR) ^{1}H, ^{13}C, and ^{11}B spectra were obtained on a 400 MHz NMR (*Jeol JNM EX-400*) and 500 MHz NMR (*Jeol JNM EX-500)*, or on a Bruker spectrometer AV III HD 700, AV III 600 or AV NEO 400 at the NMR centre of the University of Vienna. All spectra were obtained at room temperature (rt). Carbon spectra were acquired with a complete decoupling for the proton. Boron spectra were measured in quartz NMR tubes. Proton and carbon chemical shifts are reported in parts per million (ppm, δ scale) according to tetramethylsilane ($\delta_H = \delta_C = 0$ ppm) using the solvent residual signal (*) as an internal reference ($CDCl_3$: δ_H = 7.26 ppm, δ_C = 77.16 ppm; CD_2Cl_2: δ_H = 5.32 ppm, δ_C = 54.00 ppm). Boron chemical shifts are reported in ppm, referencing the external standard boron signal of $BF_3{\cdot}Et_2O$ (δ_B = 0 ppm). Coupling constants (*J*) are given in Hz. Resonance multiplicity is described as s (singlet), d (doublet), dd (doublet of doublets), t (triplet), q (quartet), p (pentet), m (multiplet), and bs (broad signal).

Infrared spectra (IR) were recorded on a Perkin-Elmer Spectrum II FT-IR System with Specac Silver Gate Evolution single-reflection ATR mounted with a diamond mono-crystal or a Bruker Alpha FT-IR spectrometer in ATR mode. Selected absorption bands are reported in wavenumbers (cm^{-1}).

High-resolution mass spectrometry ***(HRMS)*** analyses were performed by the Centre de spectrométrie de masse at the Université de Mons in Belgium and the Mass Spectrometry

Centre at the University of Vienna. MALDI-MS were recorded using a Waters QtoF Premier mass spectrometer equipped with, operating at 337 nm with a maximum output of 500 mW delivered to the sample in 4 ns pulses at 20 Hz repeating rate. Time-of-flight analyses were performed in the reflectron mode at a resolution of about 10,000. The matrix, trans-2-[3-(4-tert-butyl-phenyl)-2-methyl-2-propenylidene]malonitrile (DCTB), was prepared as a 40 mg/mL solution in $CHCl_3$. The matrix solution (1 μL) was applied to a stainless-steel target and air-dried. Analyte samples were dissolved in a suitable solvent to obtain 1 mg/mL solutions. 1 μL aliquots of those solutions were applied onto the target area already bearing the matrix crystals, and air-dried. For recording the single-stage MS spectra, the quadrupole (rf-only mode) was set to pass ions from 100 to 1000 Th, and all ions were transmitted in the pusher region of the time-of-flight analyser mass. Alternatively, MALDI-MS spectra were recorded on a Bruker Autoflex Speed MALDI-timsTOF (matrix: 2-[(2E)-3-(4-tert-butylphenyl)-2-methylprop-2-enylidene]malononitrile (DCTB)) mass spectrometer. ESI mass spectra were obtained on a Bruker maXis UHR ESI-Qq-TOF mass spectrometer in the positive ion mode, and GC mass spectra were measured on an Agilent 7200B GC/Q-TOF mass spectrometer.

S1.2 Materials and methods

Chemicals were purchased from Sigma Aldrich, Acros Organics, TCI, ABCR, Alfa Aesar, Fluorochem, Thermo Fisher Scientific, and BLDpharm and used as received. Solvents were purchased from Sigma Aldrich, while deuterated solvents were purchased from Eurisotop. THF was distilled from sodium-benzophenone, and toluene was refluxed over calcium hydride. Aniline was distilled from calcium hydride. Anhydrous conditions were achieved by drying glassware in oven at 120 °C for at least 12 h and by flaming the reaction vessels with a heat gun under vacuum and purging with argon. The inert atmosphere was maintained using argon-filled balloons equipped with a syringe and needle that was used to penetrate the silicon septa used to close the flask's necks. As an alternative to Schlenk line techniques, inert conditions were achieved using an argon-filled MBraun LabStar glove box when stated. The addition of liquid reagents was performed using argon-purged plastic or glass syringes. The solutions were degassed by freeze-pump-thaw procedure: solutions were frozen in liquid nitrogen and kept under a vacuum for 10–15 min before thawing. Low-temperature baths were prepared using different solvent mixtures depending on the desired temperature: -84°C with liquid N2/EtOAc and 0 °C with ice/water.

S2 Synthetic procedures and spectral data

S2.1 Synthesis of 4-(phenylethynyl)aniline 1^{An}

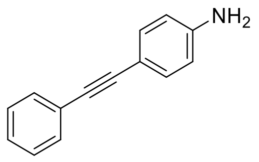

To a solution of 4-iodoaniline (2.00 g, 9.14 mmol) in diisopropylamine (25 mL), [$Pd(PPh_3)_2Cl_2$] (0.066 g, 0.098 mmol) and CuI (0.019 g, 0.098 mmol) were added. The mixture was subjected to three freeze-pump-thaw cycles, and phenylacetylene (1.05 mL, 9.60 mmol) was added. The reaction mixture was stirred for 3 h at room temperature, then it was diluted with EtOAc (50 mL), washed with H_2O (3 × 70 mL) and brine (70 mL). The organic layer was dried over $MgSO_4$ and evaporated under reduced pressure. The residue was purified by recrystallization from hot hexane, affording **2** as a white crystalline solid (1.69 g, 95 % yield). Spectral properties are in agreement with those reported in the literature.[1]

^{1}H NMR (400 MHz, $CDCl_3$) δ 7.50 (dd, *J* = 7.8, 1.7, 2H), 7.36–7.29 (m, 5H), 6.64 (d, *J* = 8.5, 2H), 3.82 (bs, 2H). ^{13}C NMR (100 MHz, $CDCl_3$) δ 146.8, 133.0, 131.4, 128.4, 127.8, 123.9, 114.8, 112.6, 90.2, 87.4. EI-HRMS calc. for $[C_{14}H_{11}N]^+$: 194.0970; found: 194.0985.

S2.2 Synthesis of ((4-bromo-3,5-dimethylphenyl)ethynyl)trimethylsilane 3

Prepared according to literature procedure.[2]

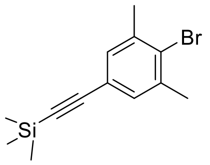

In a 150 mL Schlenk tube, to a solution of 2-bromo-5-iodo-*m*-xylene (2.99 g, 9.60 mmol) in diisopropylamine (60 mL), [$PdCl_2(PPh_3)_2$] (202 mg, 0.288 mmol) and CuI (110 mg, 0.576 mmol) were added. The mixture was subjected to three freeze-pump-thaw cycles and trimethylsilylacetylene (1.64 mL, 11.5 mmol) was added. The mixture was stirred at room temperature for 16 h, then diluted with water (50 mL) and extracted with CH_2Cl_2 (3 x 80 mL). The combined organic layers were dried over Na_2SO_4 and the solvents removed under reduced pressure. The residue was purified by short silica gel plug filtration (heptane), affording **3** as a yellow oil (2.78 g, quantitative yield). The product was further purified by distillation over

CaH_2 (0.04 mbar, 105 °C) for subsequent lithiation reaction. Spectral properties are in agreement with those reported in the literature.[2]

^{1}H NMR (400 MHz, $CDCl_3$) δ 7.19 (s, 2H), 2.38 (s, 6H), 0.27 (s, 9H). ^{13}C NMR (101 MHz, $CDCl_3$) δ 138.4, 131.4, 128.3, 121.6, 104.6, 94.6, 23.8, 0.09. GC-HRMS $[M]^+$ calc. for $[C_{13}H_{17}BrSi]^+$: 282.0257, found: 282.0251.

S2.3 Synthesis of *N*,*N'*,*N''*-tri(4-phenylethynyl)phenyl-*B*,*B'*,*B''*-tri(2,6-dimethyl-4-((trimethylsilyl)ethynyl)phenyl)borazine 1^{TMS}

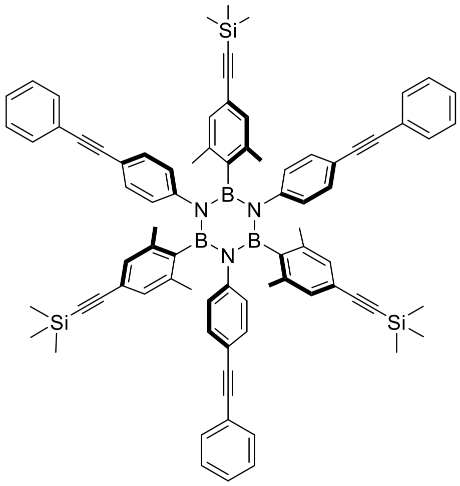

To a solution of **1^{An}** (1.40 g, 7.25 mmol) in dry toluene (20 mL) under argon, a solution of BCl_3 (1 M in heptane, 15 mL, 15 mmol) was added dropwise at 0 °C. The resulting white suspension was refluxed for 24 h. The solution was cooled down to room temperature and stripped with five freeze-pump-thaw cycles to remove the excess of HCl. In parallel, to a solution of **3** (3.0 g, 8.2 mmol) in anhydrous THF (40 mL), tBuLi (1.7 M in hexane, 10 mL, 15 mmol) was added dropwise at -84 °C. The solution was stirred at 0 °C for 10 min. The degassed toluene mixture containing the trichloro-borazine intermediate was cannulated dropwise to the organometallic-containing solution at 0 °C and stirred at room temperature for 16 h. The reaction mixture was diluted with EtOAc (100 mL), washed with H_2O (3 x 100 mL) and brine (1 x 100 mL). The organic layer was dried over $MgSO_4$ and evaporated under reduced pressure. The residue was purified by silica gel column chromatography (cyclohexane/CH_2Cl_2 9:1), affording **1^{TMS}** as a white solid (0.50 g, 13 % yield).

mp > 300 °C. ^{1}H NMR (400 MHz, $CDCl_3$) δ 7.44–7.42 (m, 6H), 7.30–7.28 (m, 9H), 6.99–6.97 (m, 6H), 6.76–6.75 (m, 12H), 2.24 (s, 18H), 0.16 (s, 27H). ^{13}C NMR (100 MHz, $CDCl_3$) δ

145.4, 137.5, 131.63, 131.55, 130.9, 128.41, 128.38, 128.2, 126.7, 123.3, 121.9, 120.0, 105.8, 93.4, 89.5, 89.1, 23.0, 0.06. ^{11}B NMR (128 MHz, $CDCl_3$) *δ* 35.6. MALDI-HRMS $[M]^+$ calc. for $[C_{81}H_{78}B_3N_3Si_3]^+$: 1210.5861, found: 1210.5881. IR (film in CH_2Cl_2) ν_{max} (cm^{-1}): 2969, 2151, 1738, 1596, 1491, 1358, 1308, 1289, 1246, 1228, 1216, 1157, 1024, 957, 840, 759, 697, 656, 563, 528.

S2.4 Synthesis of *N*,*N'*,*N''*-tri(4-phenylethynyl)phenyl-*B*,*B'*,*B''*-tri(2,6-dimethyl-4-ethynylphenyl)borazine 1^{yne}

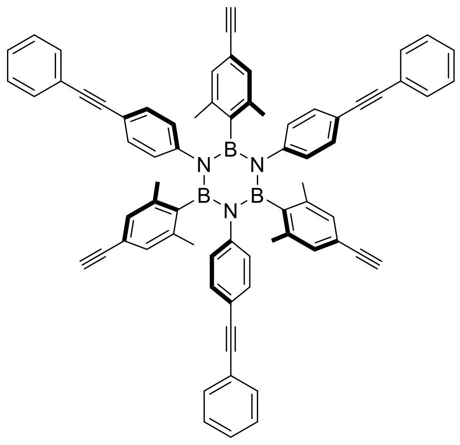

To a solution of **1^{TMS}** (0.32 g, 0.27 mmol) in THF (5 mL), TBAF (1 M solution in THF, 0.9 mL, 0.90 mmol) was added dropwise at 0 °C. The reaction mixture was stirred at room temperature for 1 h, then diluted with EtOAc (30 mL), washed with H_2O (3 x 30 mL) and brine (30 mL). The organic layer was dried over $MgSO_4$ and the solvents removed under reduced pressure. The residue was purified by silica gel column chromatography (cyclohexane/CH_2Cl_2 6:4), affording **1^{yne}** as a white solid (0.132 g, 54 % yield).

^{1}H NMR (300 MHz, $CDCl_3$) δ 7.44–7.41 (m, 6H), 7.30–7.28 (m, 9H), 7.01–6.98 (m, 6H), 6.77–6.75 (m, 12H), 2.91 (s, 3H), 2.26 (s, 18H). ^{13}C NMR (100 MHz, $CDCl_3$) *δ* 145.4, 140.0, 137.6, 131.6, 130.9, 129.6, 128.4, 128.3, 126.8, 123.2, 121.0, 119.9, 89.5, 89.1, 84.2, 76.7, 23.0. ^{11}B NMR (128 MHz, $CDCl_3$) δ 36.1. MALDI-HRMS $[M]^+$ calc. for $[C_{72}H_{54}B_3N_3]^+$: 994.4675, found: 994.4661. IR (film in CH_2Cl_2) ν_{max} (cm^{-1}): 3280, 2965, 2220, 1725, 1625, 1533, 1490, 1442, 1435, 1325, 1315, 1222, 1216, 1088, 1014, 890, 874, 827, 766, 745, 730, 695, 675, 648, 614, 600, 566, 545, 528, 502.

S2.5 Synthesis of 2-(tritylthio)ethanol 5

Prepared according to literature procedure.[3]

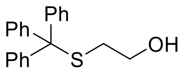

To a solution of mercaptoethanol (1.26 g, 16.1 mmol) in THF (10 mL), trityl chloride (5.40 g, 19.4 mmol) was added at room temperature. The reaction mixture was stirred at 50 °C for 4 h. The solvent was removed under reduced pressure, and the residue purified by silica gel chromatography (CH2Cl2/heptane 8:2 to CH2Cl2/EtOAc 8:2), affording **5** as a white solid (3.62 g, 70 % yield). Spectral properties are in agreement with those reported in the literature.[4]

^{1}H NMR (700 MHz, $CDCl_3$) δ 7.45–7.41 (m, 6H), 7.31–7.27 (m, 6H), 7.24–7.20 (m, 3H), 3.38 (q, *J* = 6.2, 2H), 2.49 (t, *J* = 6.2, 2H). ^{13}C NMR (176 MHz, $CDCl_3$) δ 144.9, 129.7, 128.1, 126.9, 66.8, 61.0, 35.4. ESI-HRMS $[M+H]^+$ calc. for $[C_{21}H_{21}OS]^+$: 343.1127, found: 343.1128.

S2.6 Synthesis of (2-azidoethyl)(trityl)sulfane 4

Prepared according to literature procedure.[5]

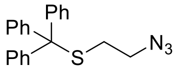

In a 25 mL oven-dry Schlenk tube, a solution of mesyl chloride (535 mg, 4.67 mmol) in dry CH_2Cl_2 (4 mL) was added dropwise to a solution of 2-(tritylthio)ethanol **5** (1.00 g, 3.12 mmol) and Et_3N (1.30 mL, 9.36 mmol) in dry CH_2Cl_2 (8 mL) at 0 °C. The white suspension was stirred at 0 °C for 30 min and at rt for 2 h. The reaction was quenched with water (30 mL) and extracted with CH_2Cl_2 (3 x 50 mL). The combined organic layers were washed with a sat. aq. solution of NH_4Cl (50 mL) and brine (50 mL), dried over Na_2SO_4, and concentrated under reduced pressure. The crude material was dissolved in dry DMF (20 mL), and NaN_3 (406 mg, 6.24 mmol) was added. The reaction mixture was stirred at 70 °C for 3 h. Water (50 mL) was added, and the aqueous phase was extracted with Et_2O (2 x 80 mL). The combined organic layers were washed with brine (4 x 60 mL), dried over Na_2SO_4, and the solvent removed under reduced pressure. The crude material was dissolved in CH_2Cl_2 (20 mL), MeOH (40 mL) was added, and the solution was concentrated under reduced pressure, affording **4** as white crystals (677 mg, 63 % yield). Spectral properties are in agreement with those reported in the literature.[5]

^{1}H NMR (400 MHz, $CDCl_3$) δ 7.46–7.40 (m, 6H), 7.34–7.27 (m, 6H), 7.25–7.19 (m, 3H), 2.93 (t, *J* = 7.2, 2H), 2.44 (t, *J* = 7.2, 2H). ^{13}C NMR (101 MHz, $CDCl_3$) δ 144.6, 129.7, 128.2, 127.0, 77.4, 50.4, 31.3. ESI-HRMS $[M+Na]^+$ calc. for $[C_{21}H_{19}N_3SNa]^+$: 368.1192, found: 368.1195.

S2.7 Synthesis of *N*,*N'*,*N''*-tri(4-phenylethynyl)phenyl-*B*,*B'*,*B''*-tri(2,6-dimethylphenyl-4-(1-(2-(tritylthio)ethyl)-1,2,3-triazol-5-yl))borazine 1^STr^

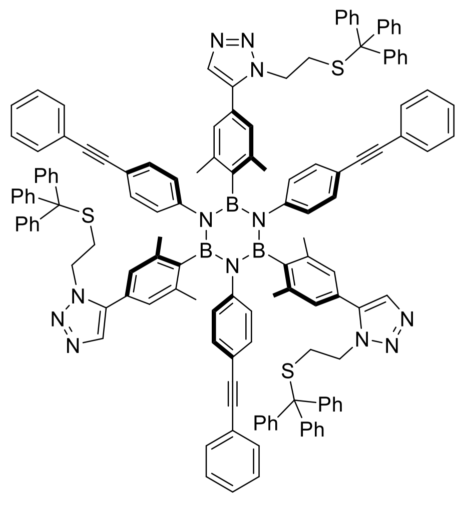

To a solution of **1**^yne^ (79 mg, 0.079 mmol) in DMF (12 mL), **4** (91 mg, 0.026 mmol) was added, followed by the successive addition of a solution of $CuSO_4 \cdot 5H_2O$ (12 mg, 0.047 mmol) in H_2O (0.5 mL) and a solution of sodium ascorbate (19 mg, 0.095 mmol) in H_2O (0.5 mL). The reaction mixture was stirred at room temperature for 16 h. A sat. aq. solution of Na_4EDTA was added, and the aqueous phase was extracted with EtOAc. The combined organic layers were washed with brine, dried, and concentrated under reduced pressure. The residue was purified by silica gel chromatography (petroleum ether/EtOAc 6:4), affording **1**^STr^ as a white solid (35 mg, 22 % yield).

mp > 300 °C. ^{1}H NMR (400 MHz, $CDCl_3$) δ 7.34–7.32 (m, 23H), 7.21–7.10 (m, 40H), 6.97 (s, 6H), 6.92–6.89 (m, 6H), 6.79–6.76 (m, 6H), 3.73 (t, J = 6.7, 6H), 2.67 (t, J = 6.8, 6H), 2.28 (s, 18H). ^{13}C NMR (100 MHz, $CDCl_3$) δ 147.9, 145.9, 144.4, 138.1, 131.5, 130.8, 129.7, 128.3, 128.2, 128.1, 127.1, 127.0, 123.4, 123.3, 119.7, 89.4, 89.2, 67.6, 49.1, 32.1, 23.3 (three signals are missing probably due to overlap). ^{11}B NMR (128 MHz, $CDCl_3$) δ: 36.9. MALDI-HRMS calc. for $[C_{135}H_{111}B_3N_{12}S_3]^+$: 2028.8607, found: 2028.8611. IR (film in CH_2Cl_2) ν_{max} (cm^{-1}): 3078, 2960, 2922, 2852, 2220, 1950, 1616, 1595, 1508, 1489, 1352, 1300, 1259, 1083, 1049, 1016, 866, 770, 756, 742, 692, 617.

S2.8 Synthesis of *N*,*N'*,*N''*-tri(4-phenylethynyl)phenyl-*B*,*B'*,*B''*-tri(2,6-dimethylphenyl-4-(1-(2-thioethyl)-1,2,3-triazol-5-yl))borazine $\mathbf{1^{SH}}$

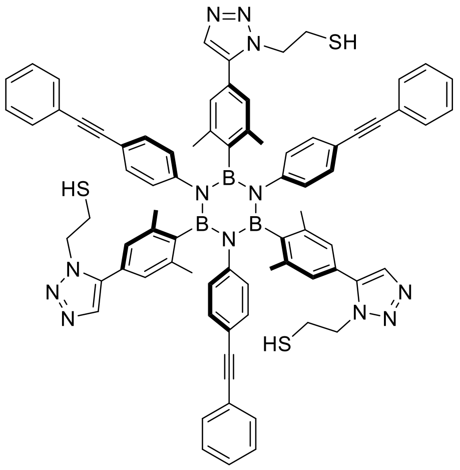

A solution of $\mathbf{1^{STr}}$ (68 mg, 0.033 mmol) in CH_2Cl_2 (5 mL) was cooled down to 0 °C. TFA (0.026 mL, 0.334 mmol) was added, followed by TIPS (0.020 mL, 0.1 mmol). The reaction mixture was stirred at room temperature for 1 h. The solvent was evaporated, the residue dissolved in CH_2Cl_2 and precipitated upon hexane slow addition at 0 °C. The precipitate was filtered and washed with MeOH, affording $\mathbf{1^{SH}}$ as a yellow solid (42 mg, 99 % yield).

mp 80 °C (dec). ^{1}H NMR (300 MHz, $CDCl_3$) δ 7.70 (s, 3H), 7.39–7.28 (m, 6H), 7.26–7.19 (m, 9H), 7.06 (s, 6H), 6.99 (d, *J* = 8.2, 6H), 6.85 (d, *J* = 8.2, 6H), 4.50 (t, *J* = 6.4, 6H), 3.07–2.88 (m, 6H), 2.36 (s, 18H), 1.43 (t, *J* = 8.8, 3H). ^{13}C NMR (100 MHz, $CDCl_3$) *δ* 147.7, 145.8, 139.7, 131.5, 131.0, 128.5, 128.4, 128.2, 126.9, 123.5, 123.2, 120.7, 119.8, 89.34, 89.29, 53.6, 24.8, 23.3 (one signal is missing probably due to overlap). ^{11}B NMR (128 MHz, $CDCl_3$) *δ* 36.0. MALDI-HRMS $[M]^+$ calc. for $[C_{78}H_{69}B_3N_{12}S_3]^+$: 1303.5310, found: 1303.5288. IR (film in CH_2Cl_2) ν_{max} (cm^{-1}): 3136, 3041, 2945, 2856, 2358, 2341, 1595, 1558, 1508, 1436, 1354, 1300, 1166, 1016, 908, 839, 756, 731, 690.

S2.9 Synthesis of *N*,*N'*,*N''*-triphenyl-*B*,*B'*,*B''*-tri(2,6-dimethyl-4-((trimethylsilyl)ethynyl)phenyl)borazine $\mathbf{2^{TMS}}$

Prepared according to literature procedure.[2]

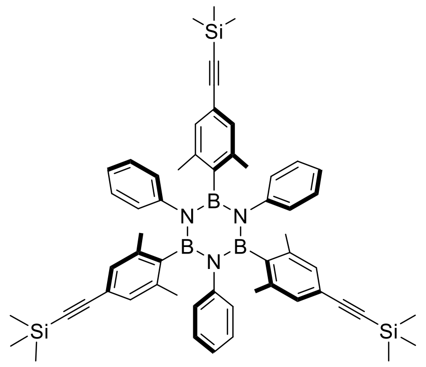

All manipulations of solids and solvents were done in an argon-filled glove box. In a 100 mL Schlenk tube, a solution of BCl_3 (1 M in heptane, 13 mL, 13 mmol) was added dropwise to a solution of distilled aniline $\mathbf{2}^{\mathbf{An}}$ (0.54 mL, 5.9 mmol) in dry toluene (10 mL) at 0 °C. The white suspension was refluxed for 20 h. The solution was allowed to reach room temperature and all volatiles were removed *in vacuo*, then the mixture was dissolved in dry THF (10 mL). Parallely, in a 100 mL Schlenk tube, *t*BuLi (2.3 M in heptane, 6.5 mL, 14.9 mmol) was added dropwise over 10 min to a solution of **3** (2.0 g, 7.1 mmol) in dry THF (31 mL) at -84 °C. The solution was stirred for 10 min at 0 °C. The chloro-borazole solution (at 0 °C) was cannulated dropwise to the aryl lithium solution (at 0 °C). The solution was stirred at 0 °C for 10 min and at room temperature for 16 h. The reaction mixture was diluted with EtOAc (100 mL), washed with water (2 x 100 mL) and brine (100 mL). The organic layer was dried over Na_2SO_4 and concentrated under reduced pressure. The residue was purified by precipitation in MeOH and silica gel plug filtration (CH_2Cl_2) affording $\mathbf{2}^{\mathbf{TMS}}$ as a white solid (466 mg, 26 % yield). Spectral properties are in agreement with those reported in the literature.[2]

^{1}H NMR (400 MHz, $CDCl_3$) δ 6.83–6.60 (m, 21H), 2.24 (s, 18H), 0.15 (s, 27H). ^{13}C NMR (151 MHz, $CDCl_3$) δ 145.4, 137.5, 129.0, 127.2, 126.5, 125.0, 121.1, 106.0, 92.8, 22.9, -0.04 (one signal is missing due to ^{11}B-induced quadrupolar relaxation). ^{11}B NMR (193 MHz, $CDCl_3$) δ 38.0. MALDI-HRMS $[M]^+$ calc. for $[C_{57}H_{66}B_3N_3Si_3]^+$: 909.4865, found: 909.4858.

S2.10 Synthesis of *N*,*N*',*N*''-triphenyl-*B*,*B*',*B*''-tri(2,6-dimethyl-4-ethynylphenyl)borazine $\mathbf{2}^{\mathbf{yne}}$

Prepared according to literature procedure.[2]

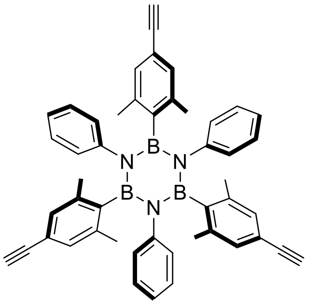

In a 10 mL round-bottom flask, TBAF (1 M solution in THF, 0.37 mL, 0.37 mmol) was added dropwise to a solution of **2$^{\mathbf{TMS}}$** (100 mg, 0.11 mmol) in THF (2.6 mL) at 0 °C. The reaction mixture was stirred at room temperature for 1 h, then diluted with brine (25 mL) and extracted with CH_2Cl_2 (3 x 40 mL). The combined organic layers were dried over Na_2SO_4 and the solvents removed under reduced pressure. The residue was purified by silica gel column chromatography (heptane/CH_2Cl_2 9:1 to 7:3) affording **2$^{\mathbf{yne}}$** as a white solid (57 mg, 75 % yield). Spectral properties are in agreement with those reported in the literature.[2]

^{1}H NMR (600 MHz, $CDCl_3$) δ 6.86–6.73 (m, 15H), 6.70 (s, 6H), 2.87 (s, 3H), 2.26 (s, 18H). ^{13}C NMR (151 MHz, $CDCl_3$) δ 145.4, 137.8, 129.3, 127.3, 126.6, 125.2, 120.4, 84.5, 76.1, 23.0 (one signal is missing due to ^{11}B-induced quadrupolar relaxation). ^{11}B NMR (193 MHz, $CDCl_3$) δ 36.2. MALDI-HRMS $[M]^+$ calc. for $[C_{48}H_{42}B_3N_3]^+$: 693.3674, found: 693.3679.

S2.11 Synthesis of *N*,*N'*,*N''*-triphenyl-*B*,*B'*,*B''*-tri(2,6-dimethylphenyl-4-(1-(2-(tritylthio)ethyl)-1,2,3-triazol-5-yl))borazine 2$^{\mathbf{STr}}$

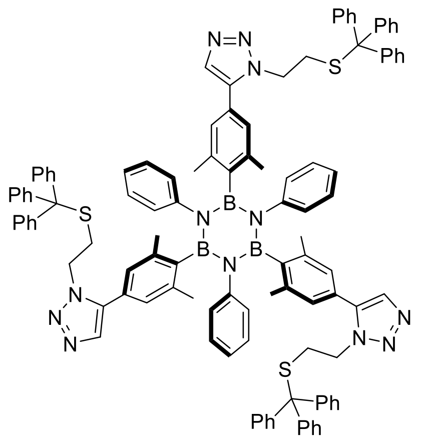

In a 100 mL round-bottom flask, to a solution of **2$^{\mathbf{yne}}$** (90 mg, 0.13 mmol) in DMF (10 mL), **4** (202 mg, 0.585 mmol) was added, followed by the successive addition of a solution of $CuSO_4 \cdot 5H_2O$ (29 mg, 0.12 mmol) in H_2O (1 mL) and a solution of sodium ascorbate (46 mg, 0.23 mmol) in H_2O (1 mL). The reaction mixture was stirred at room temperature for 40 h. A

25 % aq. solution of NH_4OH (70 mL) was added and the crude was extracted with CH_2Cl_2 (4 x 80 mL). The combined organic layers were washed with 25 % aq. NH_4OH (150 mL) and brine (2 x 150 mL), dried over Na_2SO_4 and concentrated under reduced pressure. The residue was purified by silica gel column chromatography (CH_2Cl_2 to CH_2Cl_2/EtOAc 9:1) affording **2^{STr}** as a white solid (139 mg, 62 % yield).

mp 168–170 °C. ^{1}H NMR (400 MHz, CD_2Cl_2) δ 7.44–7.36 (m, 18H), 7.34–7.25 (m, 21H), 7.22 (t, *J* = 7.2, 9H), 7.04 (s, 6H), 6.97 (d, *J* = 7.3, 6H), 6.83 (t, *J* = 7.6, 6H), 6.75 (t, *J* = 7.3, 3H), 3.83 (t, *J* = 6.9, 6H), 2.74 (t, *J* = 6.9, 6H), 2.40 (s, 18H). ^{13}C NMR (151 MHz, CD_2Cl_2) δ 147.9, 146.7, 144.9, 138.9, 130.1, 128.6, 127.7, 127.5, 127.3, 125.3, 123.1, 120.0, 67.8, 49.5, 32.5, 23.7 (two signals are missing due to ^{11}B-induced quadrupolar relaxation and overlap). ^{11}B NMR (193 MHz, CD_2Cl_2) δ 35.5. MALDI-HRMS calc. for $[C_{111}H_{99}B_3N_{12}S_3]^+$: 1729.7585, found: 1729.7667. IR ν_{max} (cm^{-1}): 3058, 3031, 2918, 1614, 1595, 1490, 1443, 1355, 1307, 1248, 1157, 1075, 1046, 1001, 860, 793, 763, 741, 697, 674, 616, 591, 562, 531, 499, 464, 450, 423, 409.

S2.12 Synthesis of *N*,*N*',*N*''-triphenyl-*B*,*B*',*B*''-tri(2,6-dimethylphenyl-4-(1-(2-thioethyl)-1,2,3-triazol-5-yl))borazine 2^{SH}

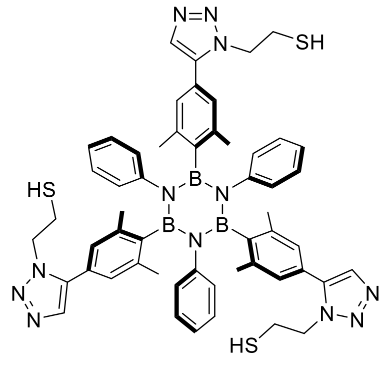

In a 25 mL round-bottom flask, a solution of **2^{STr}** (90 mg, 0.052 mmol) in CH_2Cl_2 (5 mL) was cooled down at 0 °C. TFA (0.116 mL, 1.56 mmol) was added, followed by TIPS (0.064 mL, 0.31 mmol). The reaction mixture was stirred at 0 °C for 30 min and at room temperature for 3 h. The solvent was evaporated and the residue sonicated in heptane. The precipitate was filtered affording **6** as a white solid (52 mg, quantitative yield).

mp > 260 °C. ^{1}H NMR (600 MHz, CD_2Cl_2) δ 7.70 (s, 3H), 7.05 (s, 6H), 7.01–6.98 (m, 6H), 6.88–6.83 (m, 6H), 6.80–6.75 (m, 3H), 4.50 (t, *J* = 6.5, 6H), 3.00 (dt, *J* = 8.7, 6.5, 6H), 2.41 (s, 18H), 1.48 (t, *J* = 8.7, 3H). ^{13}C NMR (151 MHz, CD_2Cl_2) δ 147.6, 146.4, 138.8, 128.9, 127.5,

127.0, 125.1, 123.0, 120.6, 25.0, 23.4 (two signals are missing due to ^{11}B-induced quadrupolar relaxation and overlap). ^{11}B NMR (193 MHz, CD_2Cl_2) δ 35.6. MALDI-HRMS $[M]^+$ calc. for $[C_{54}H_{57}B_3N_{12}S_3]^+$: 1003.4367, found: 1003.4360. IR ν_{max} (cm^{-1}): 2918, 1596, 1491, 1452, 1432, 1355, 1307, 1217, 1170, 1072, 1049, 1024, 861, 798, 764, 750, 727, 700, 664, 591, 564, 532, 480, 425.

S3 NMR spectra

S3.1 Characterization of 1^{An}

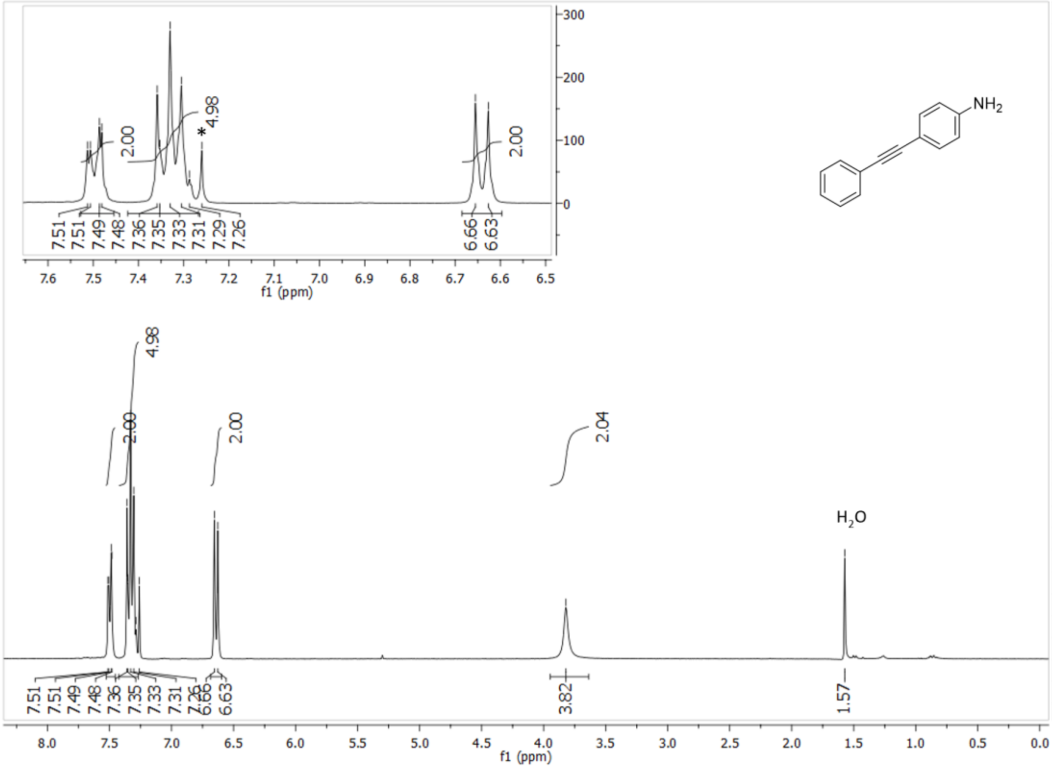

Figure S1.^{1}H NMR (400 MHz, $CDCl_3$) spectrum.

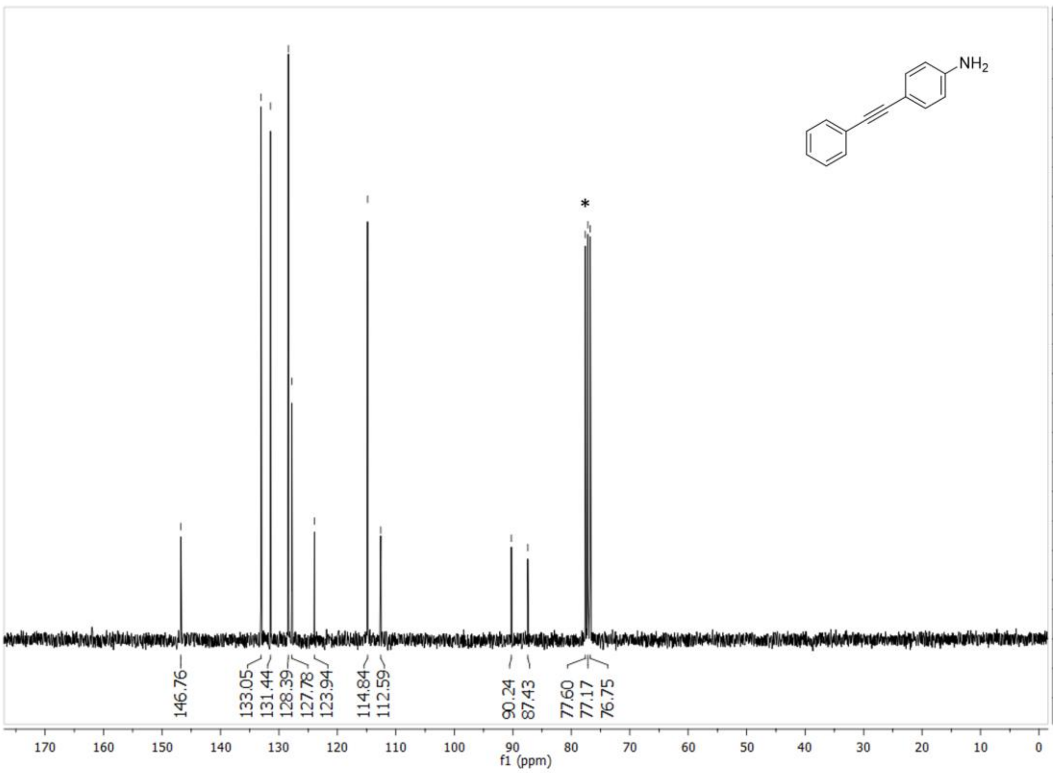

Figure S2.^{13}C NMR (100 MHz, $CDCl_3$) spectrum.

S3.2 Characterization of 3

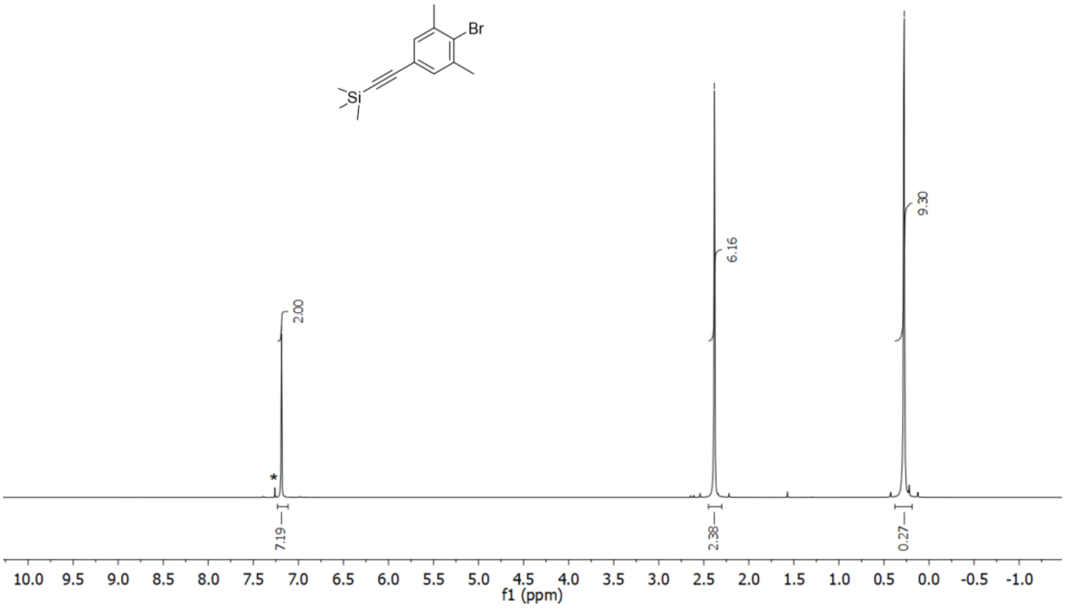

Figure S3.^{1}H NMR (400 MHz, $CDCl_3$) spectrum.

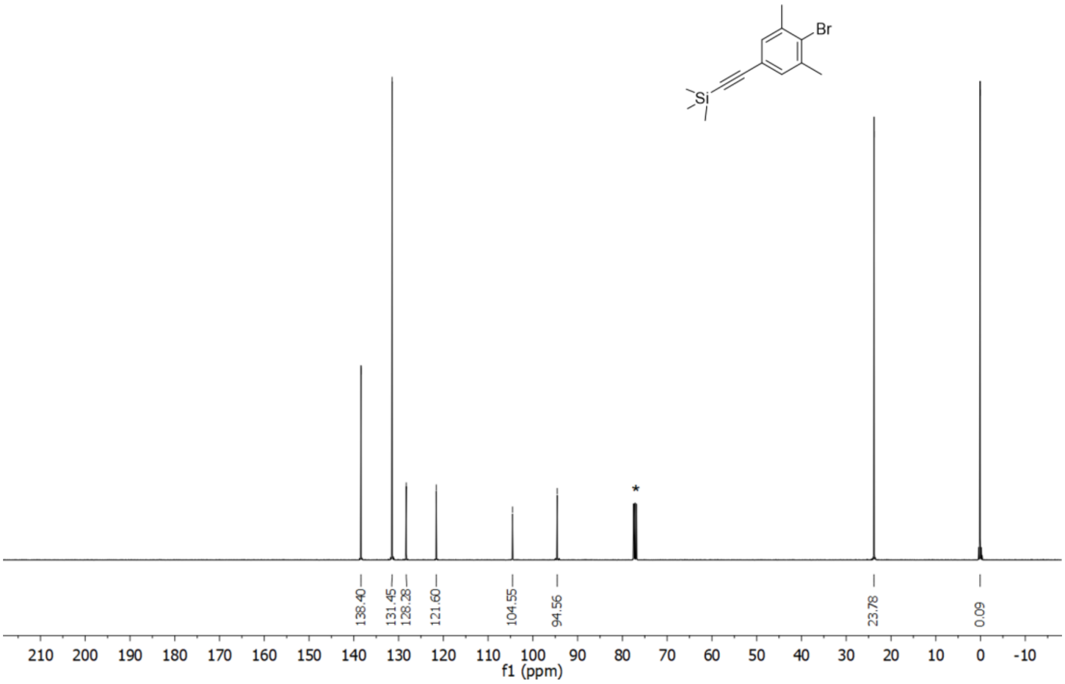

Figure S4.^{13}C NMR (101 MHz, $CDCl_3$) spectrum.

S3.3 Characterization of 1^{TMS}

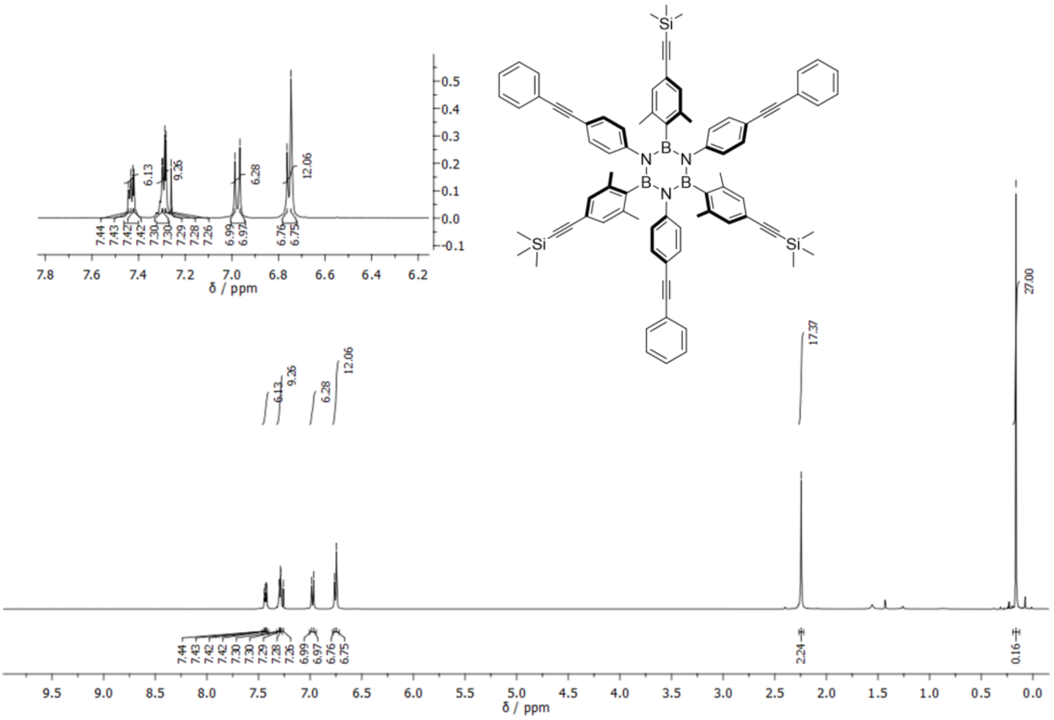

Figure S5.^{1}H NMR (400 MHz, $CDCl_3$) spectrum.

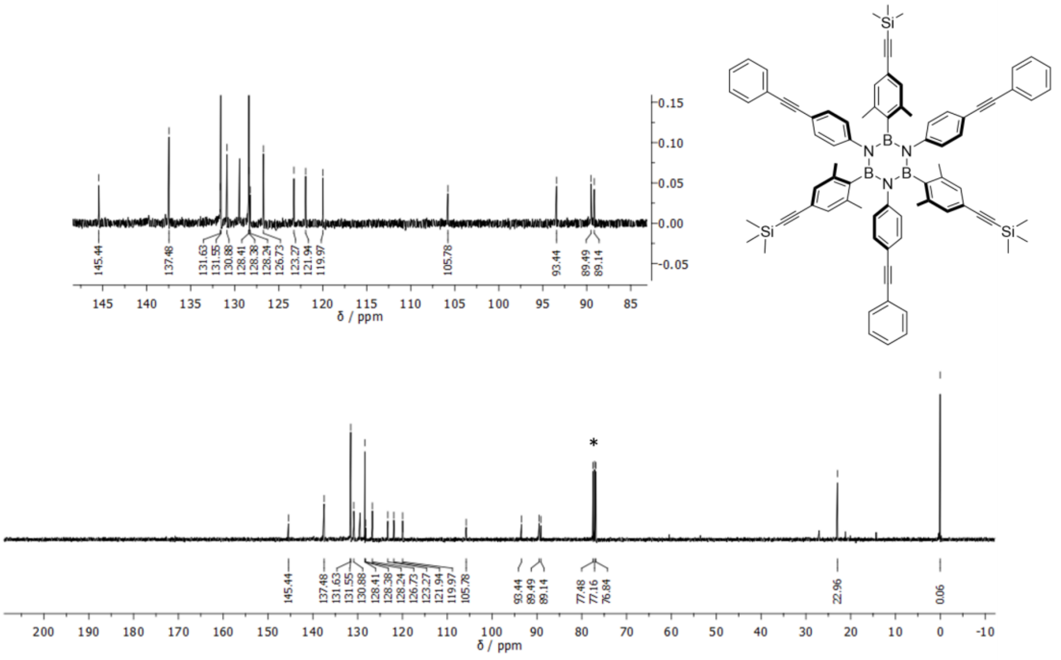

Figure S6.^{13}C NMR (100 MHz, $CDCl_3$) spectrum.

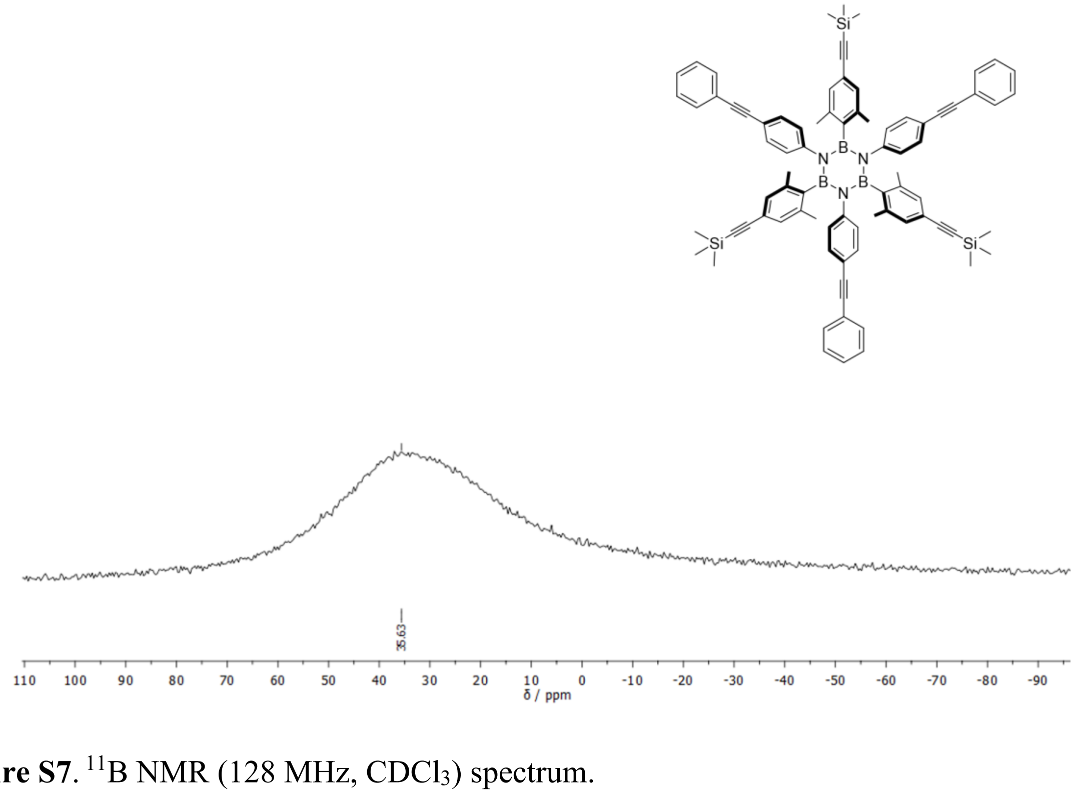

Figure S7. ^{11}B NMR (128 MHz, $CDCl_3$) spectrum.

S3.4 Characterization of 1^{yne}

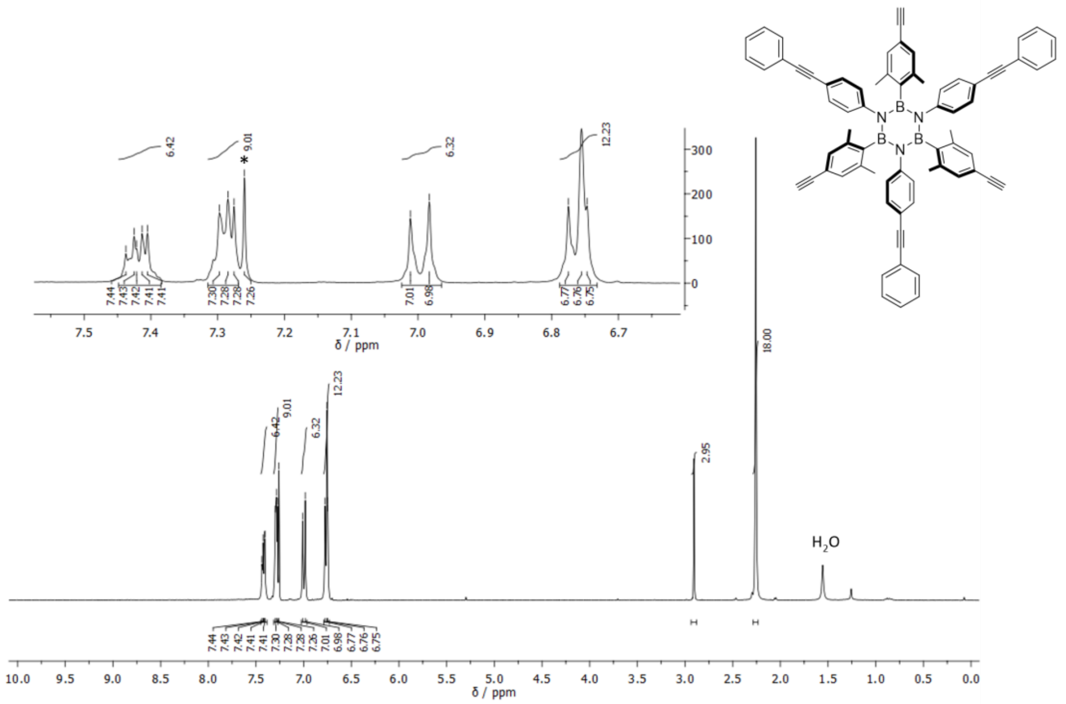

Figure S8. ^{1}H NMR (400 MHz, $CDCl_3$) spectrum.

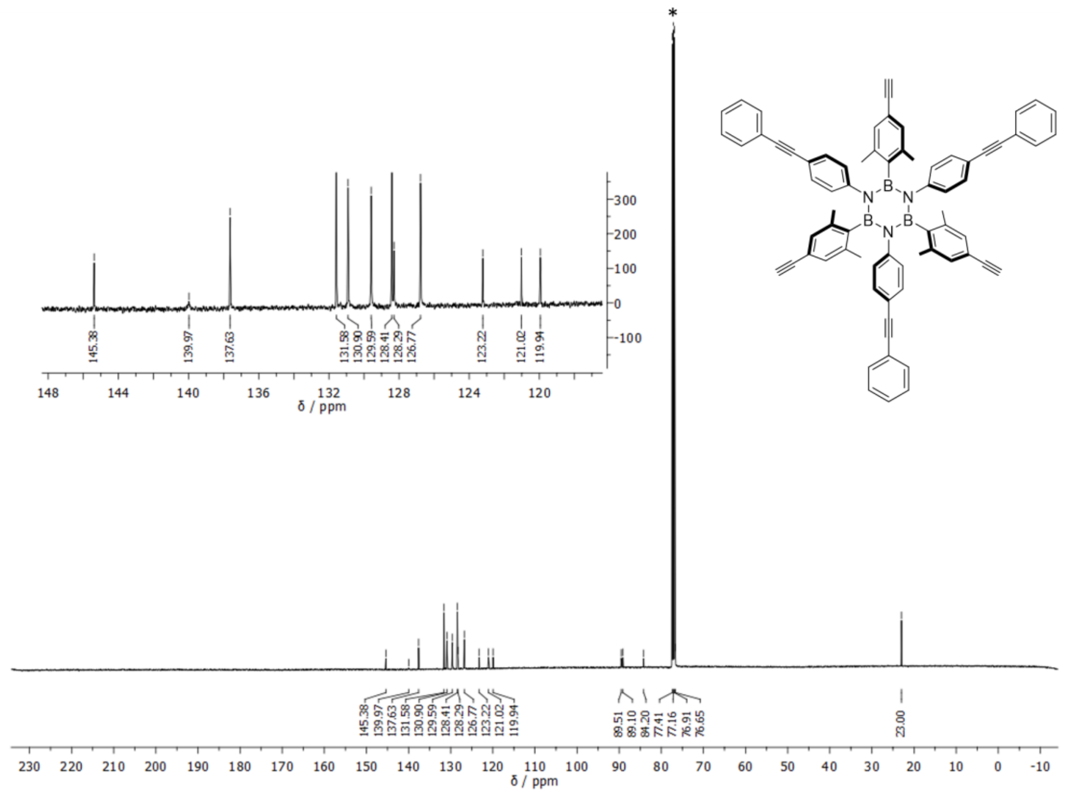

Figure S9. ^{13}C NMR (100 MHz, $CDCl_3$) spectrum.

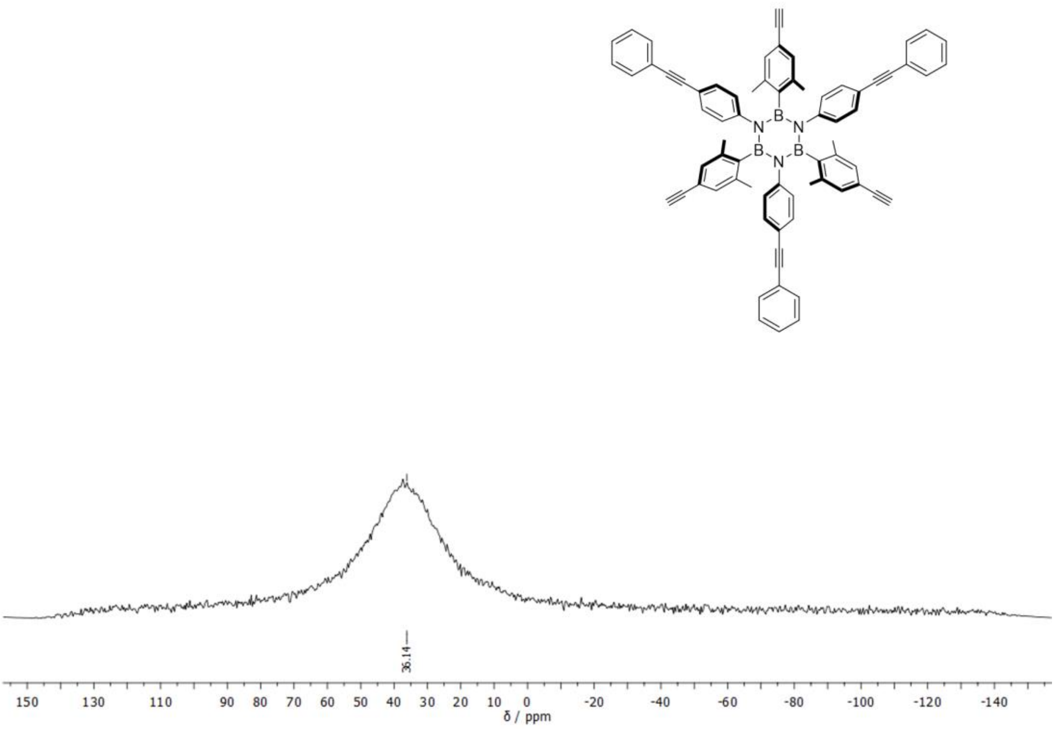

Figure S10. ^{11}B NMR (128 MHz, $CDCl_3$) spectrum.

S3.5 Characterization of 5

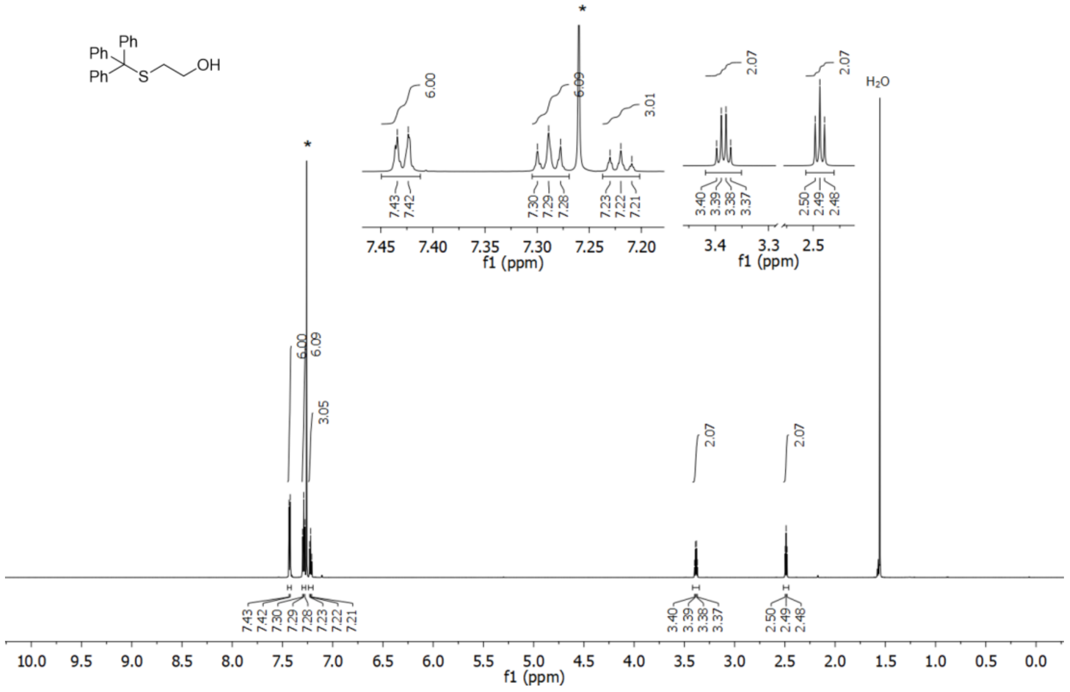

Figure S11. ^{1}H NMR (700 MHz, $CDCl_3$) spectrum.

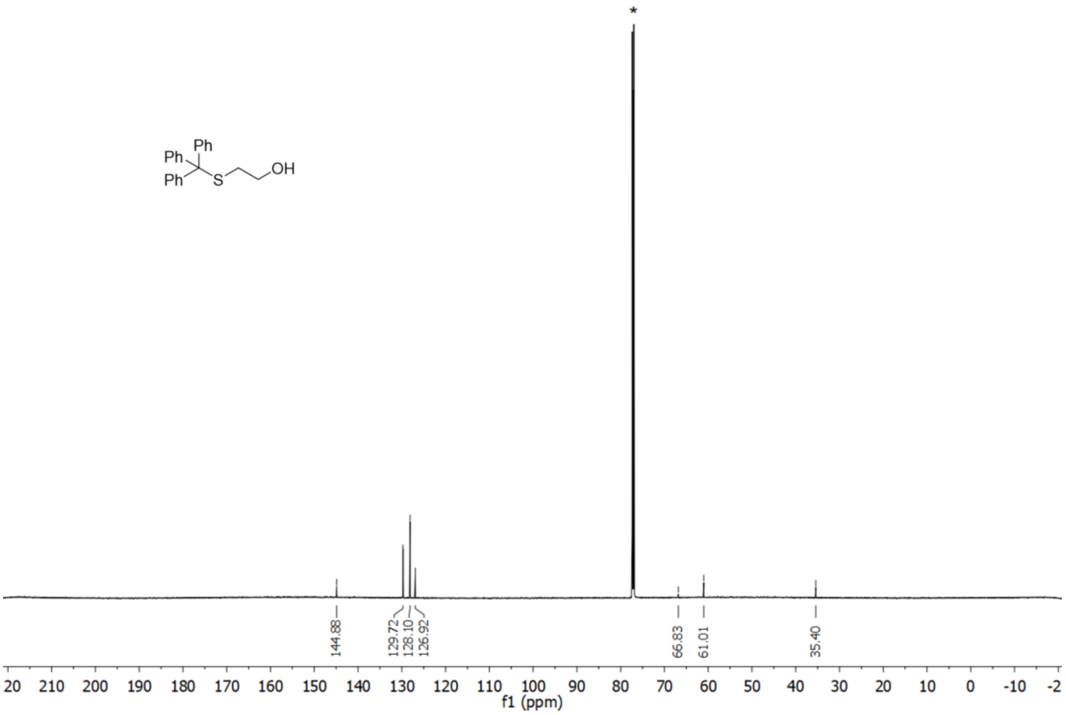

Figure S12. ^{13}C NMR (176 MHz, $CDCl_3$) spectrum.

S3.6 Characterization of 4

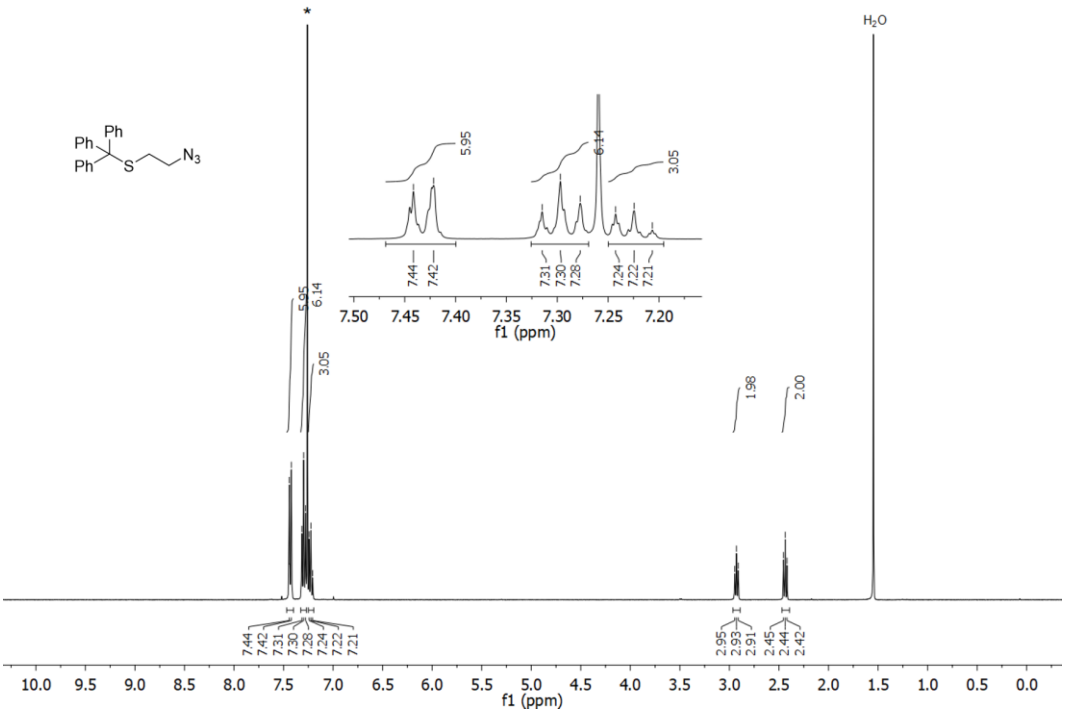

Figure S13. ^{1}H NMR (400 MHz, $CDCl_3$) spectrum.

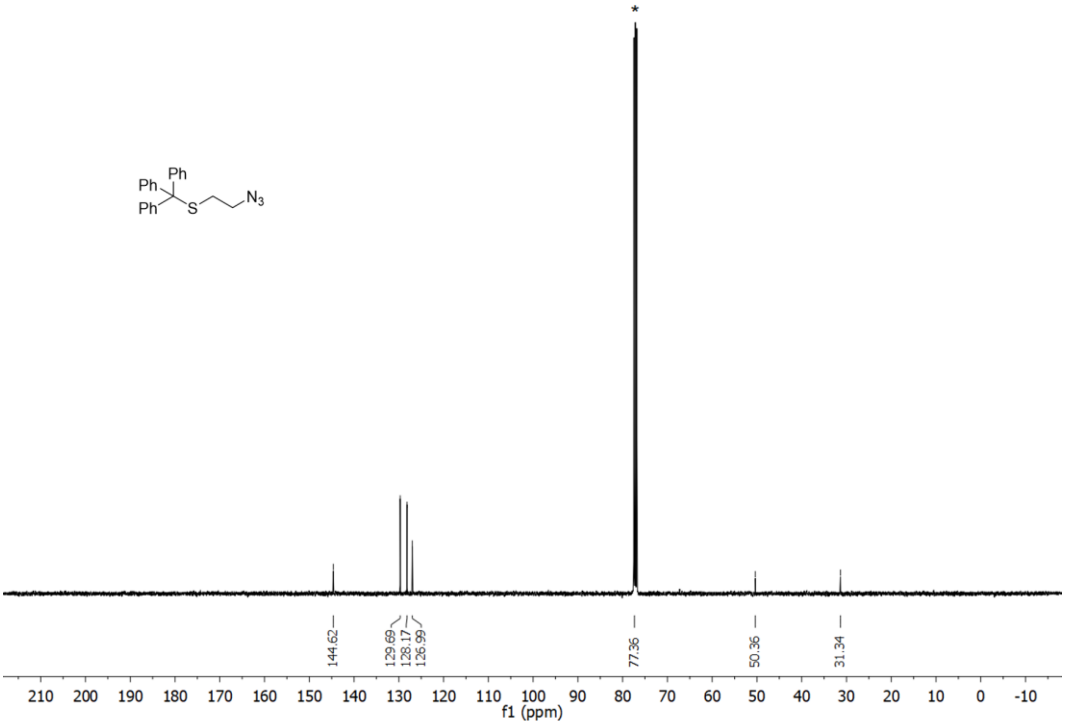

Figure S14. ^{13}C NMR (101 MHz, $CDCl_3$) spectrum.

S3.7 Characterization of 1^{STr}

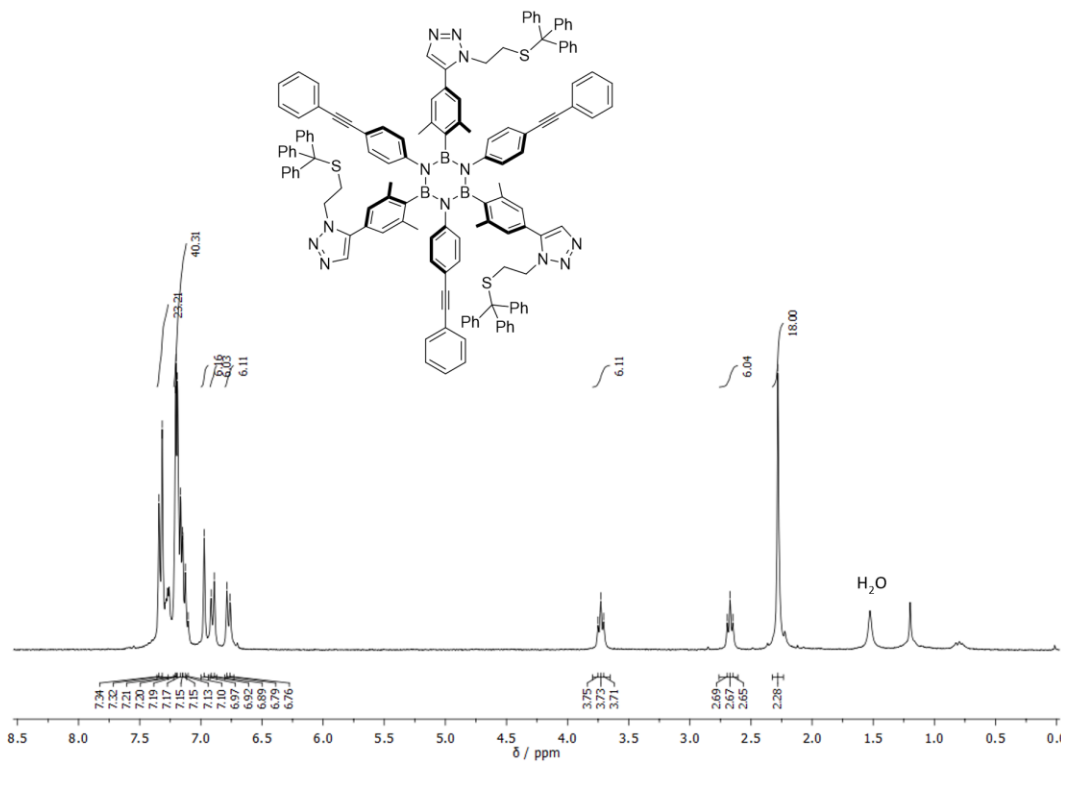

Figure S15. ^{1}H NMR (400 MHz, $CDCl_3$) spectrum.

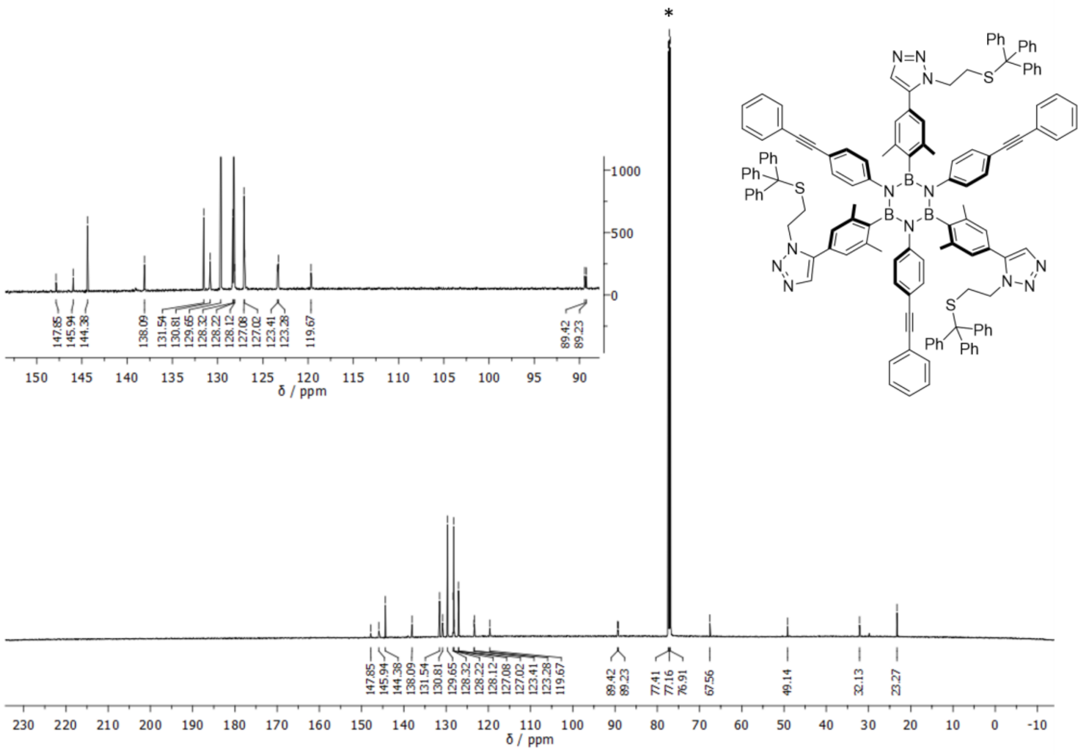

Figure S16. ^{13}C NMR (100 MHz, $CDCl_3$) spectrum.

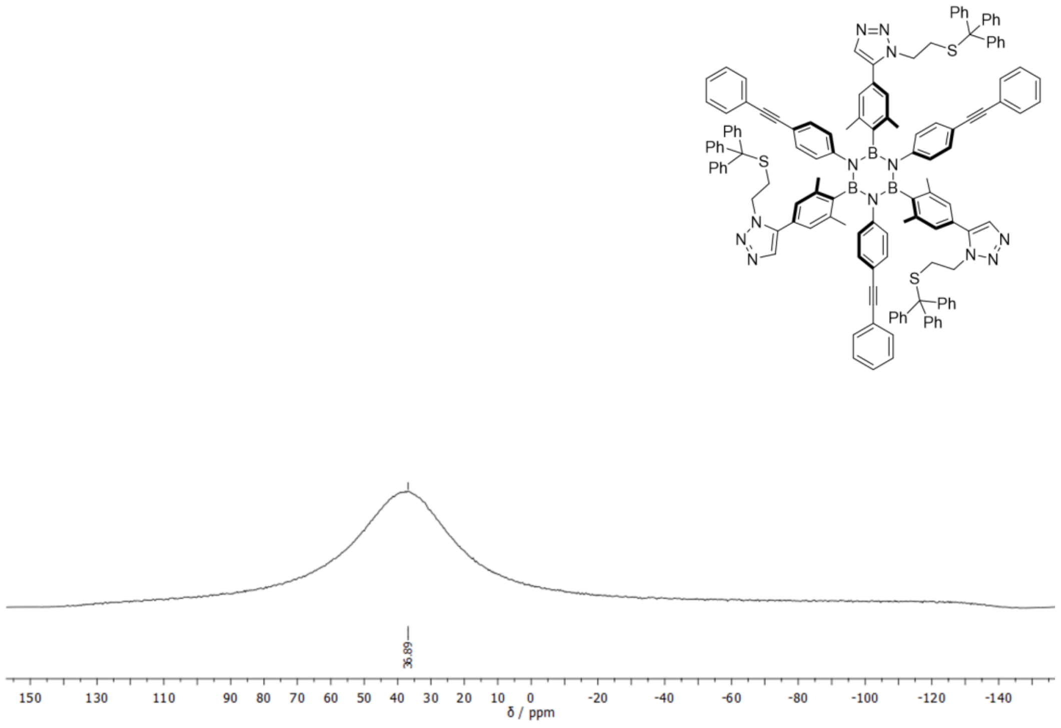

Figure S17. ^{11}B NMR (128 MHz, $CDCl_3$) spectrum.

S3.8 Characterization of 1^{SH}

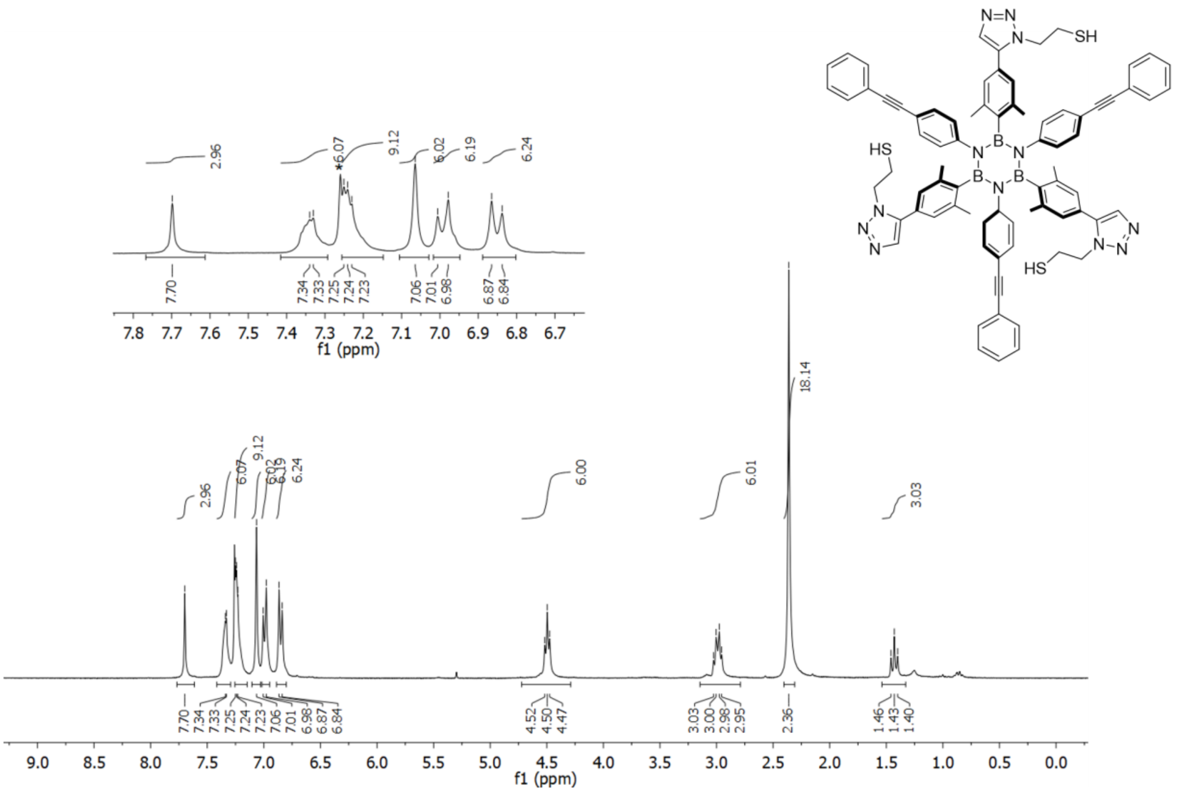

Figure S18. ^{1}H NMR (400 MHz, $CDCl_3$) spectrum.

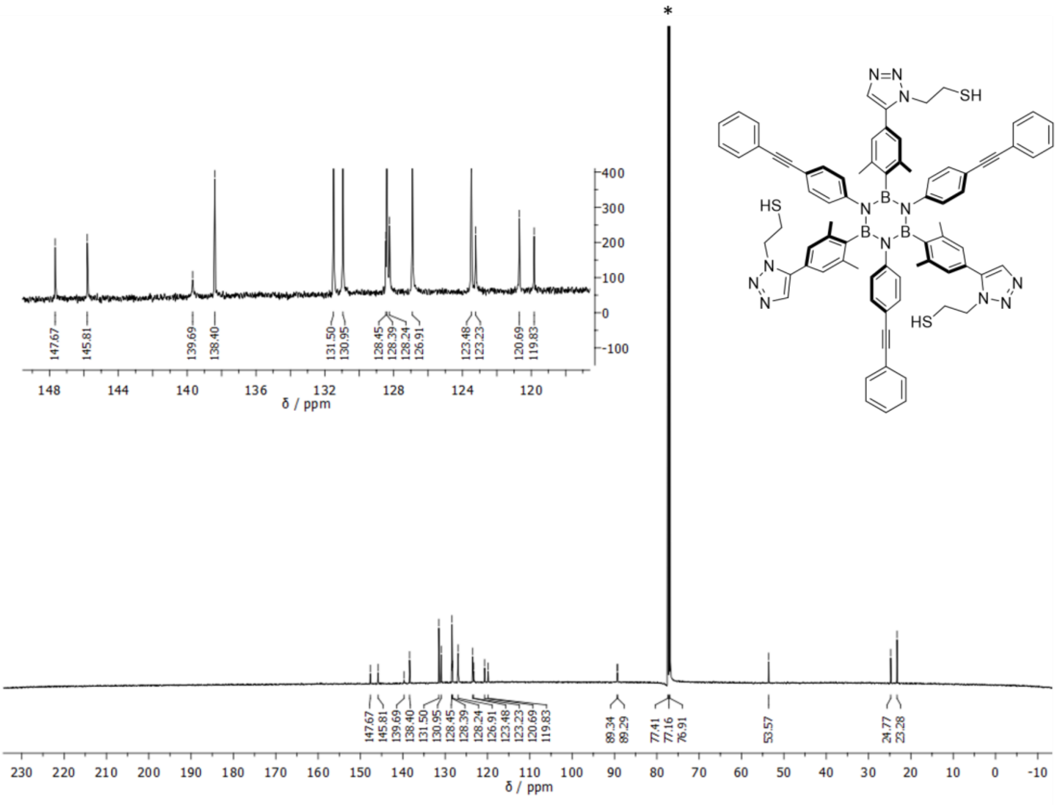

Figure S19. ^{13}C NMR (100 MHz, $CDCl_3$) spectrum.

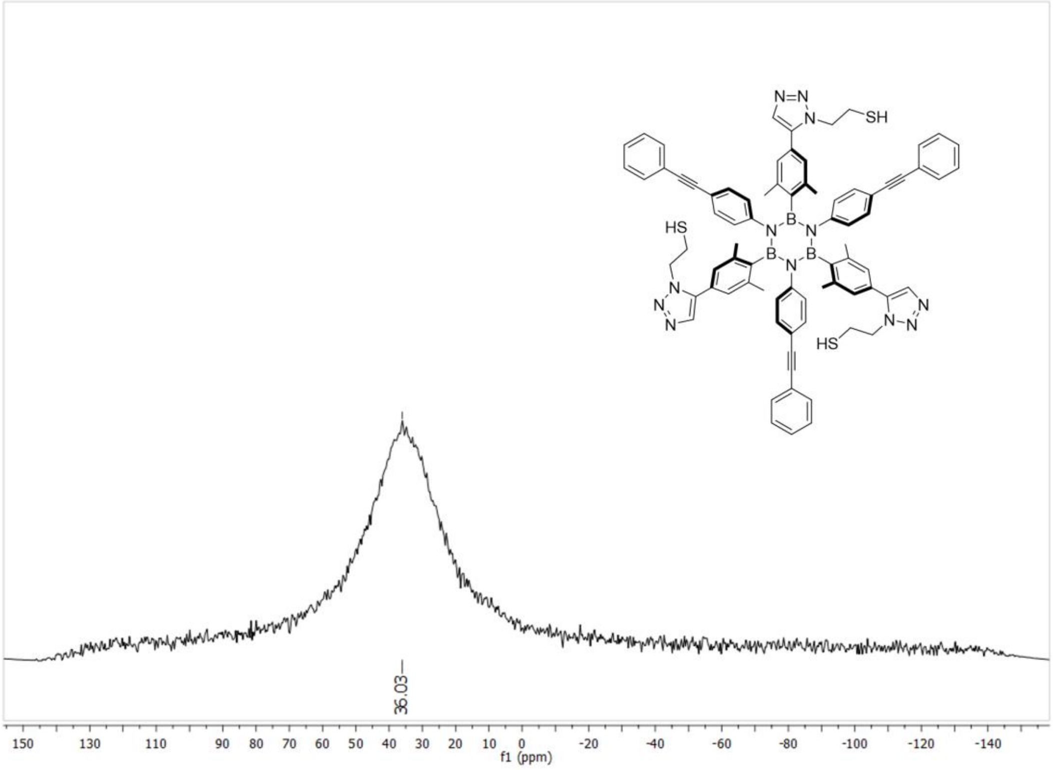

Figure S20. ^{11}B NMR (128 MHz, $CDCl_3$) spectrum.

S3.9 Characterization of 2^{TMS}

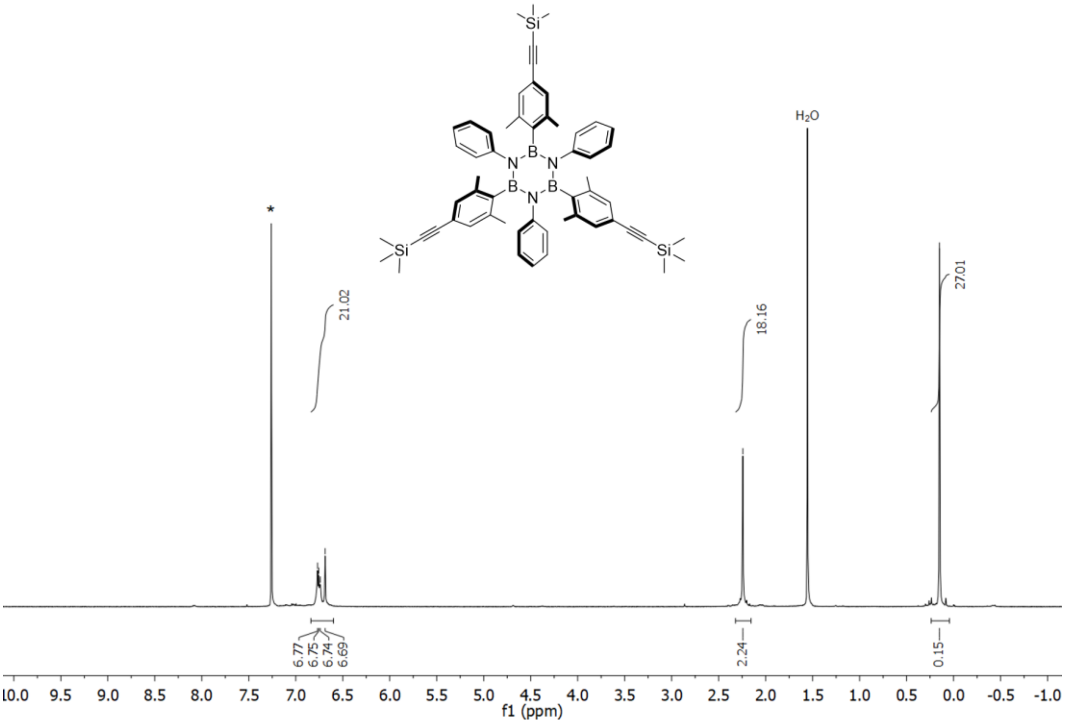

Figure S21. ^{1}H NMR (400 MHz, $CDCl_3$) spectrum.

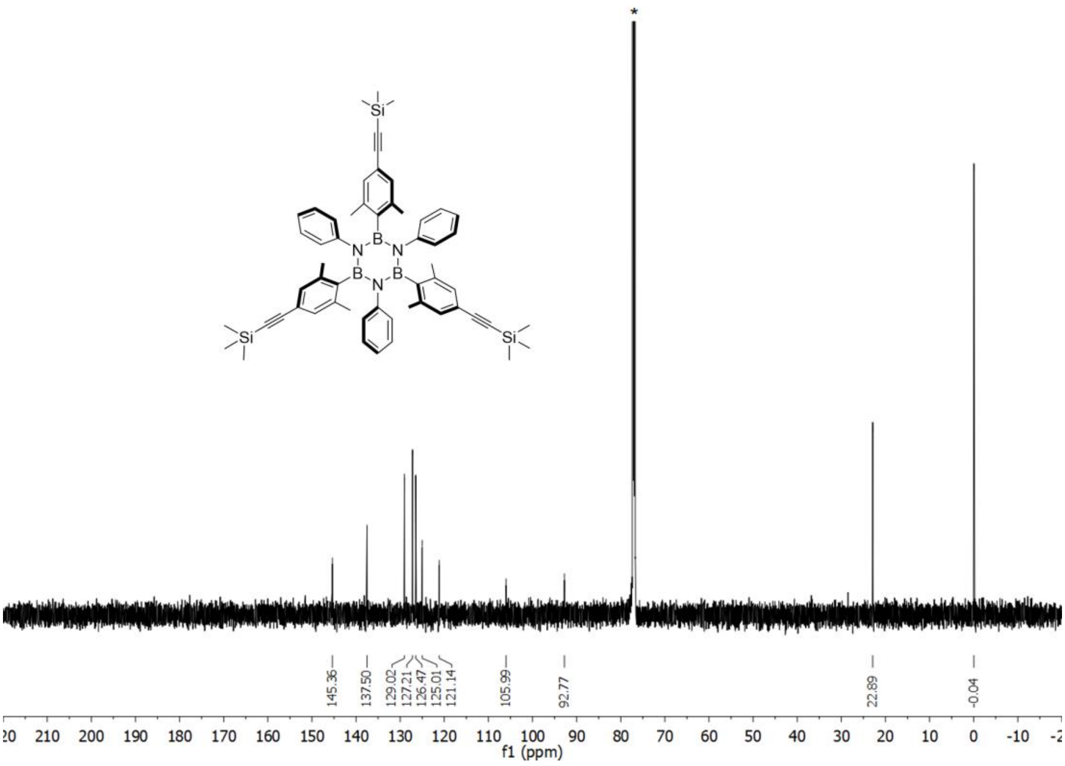

Figure S22. ^{13}C NMR (151 MHz, $CDCl_3$) spectrum.

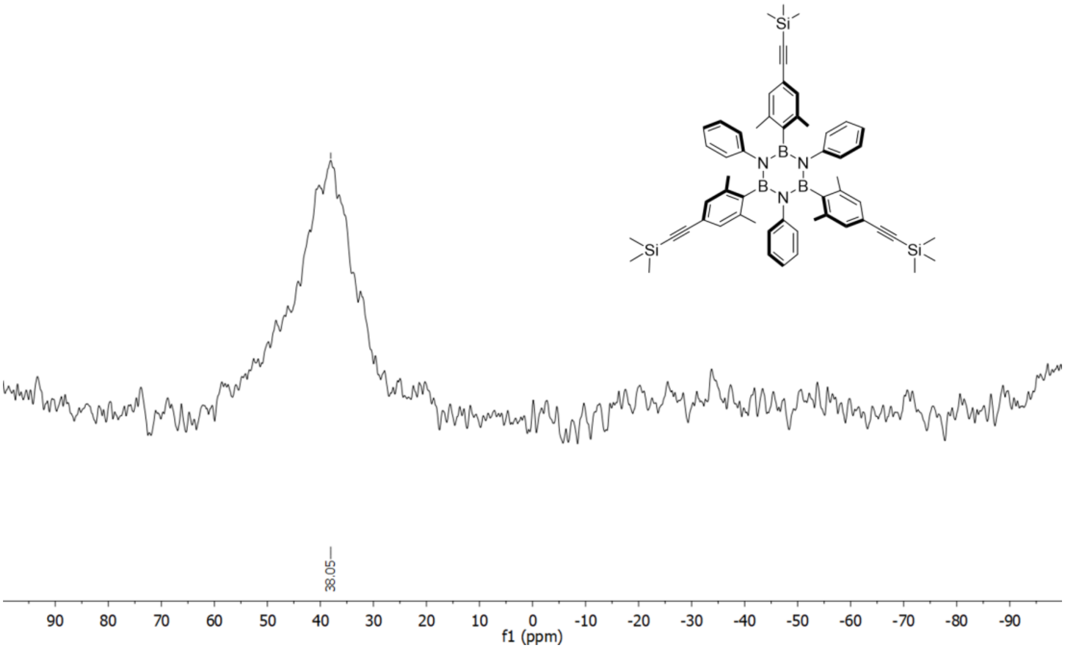

Figure S23. ^{11}B NMR (193 MHz, $CDCl_3$) spectrum.

S3.10 Characterization of 2^{yne}

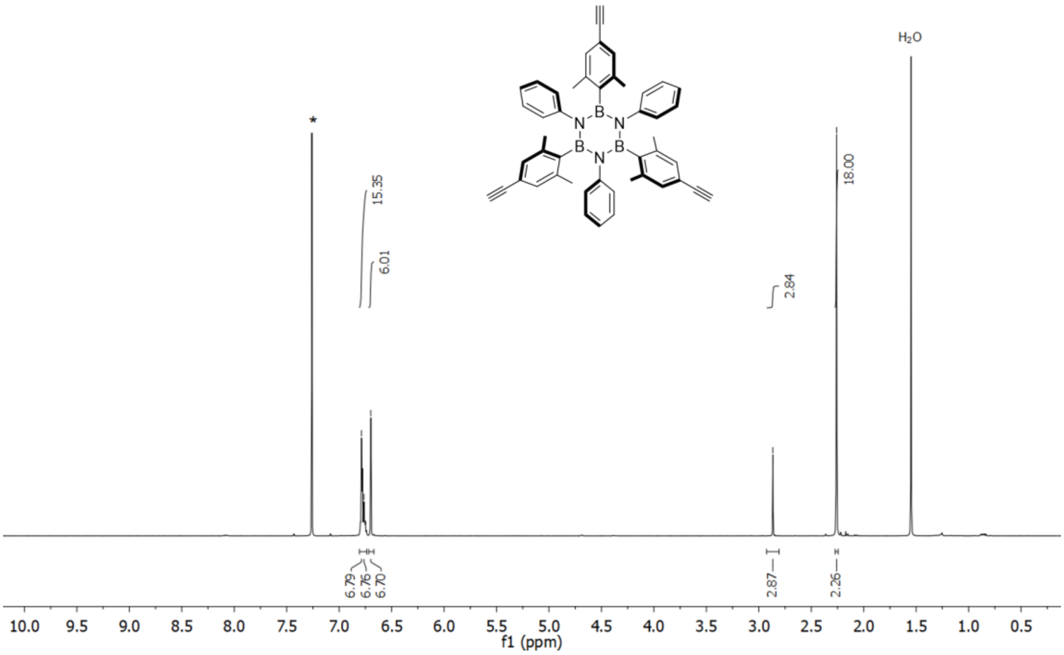

Figure S24. ^{1}H NMR (600 MHz, $CDCl_3$) spectrum.

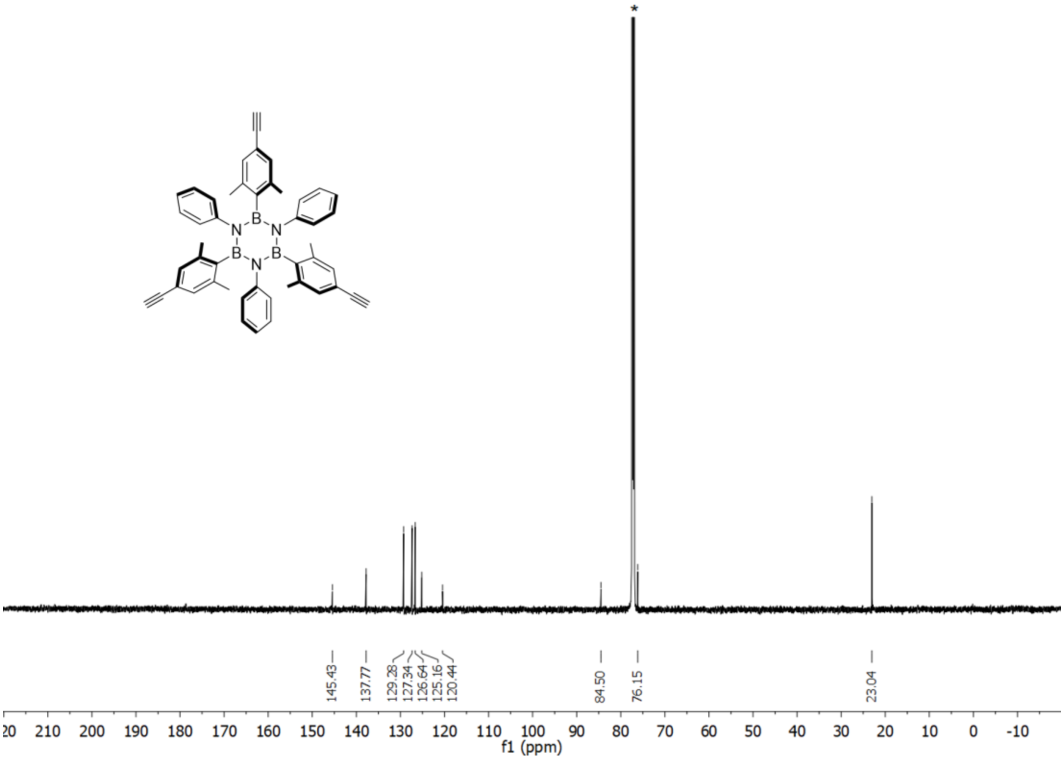

Figure S25. ^{13}C NMR (151 MHz, $CDCl_3$) spectrum.

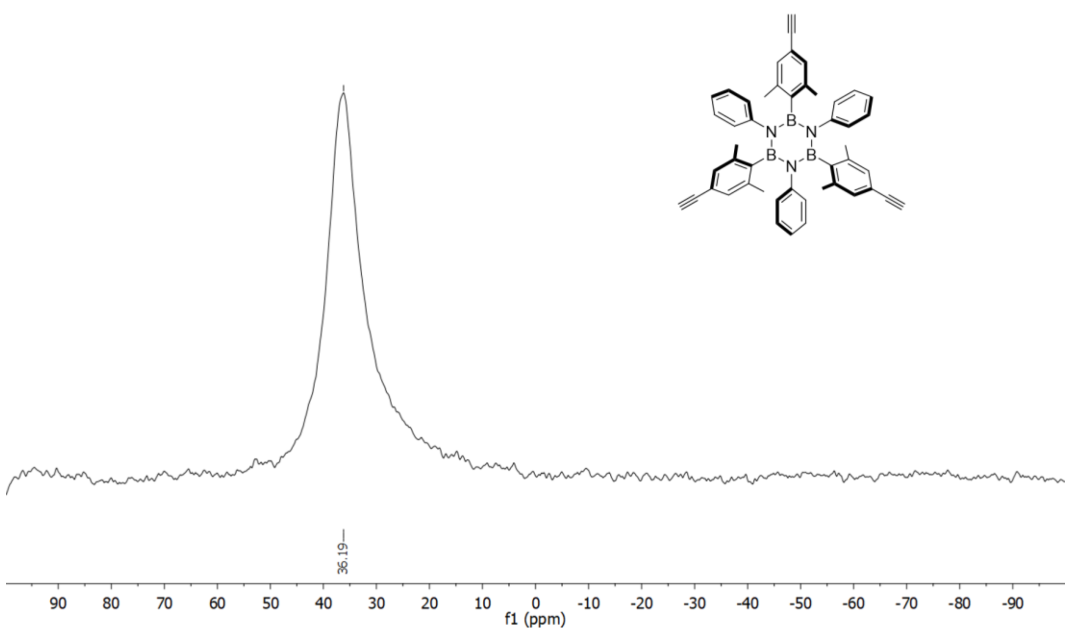

Figure S26. ^{11}B NMR (193 MHz, $CDCl_3$) spectrum.

S3.11 Characterization of 2^{STr}

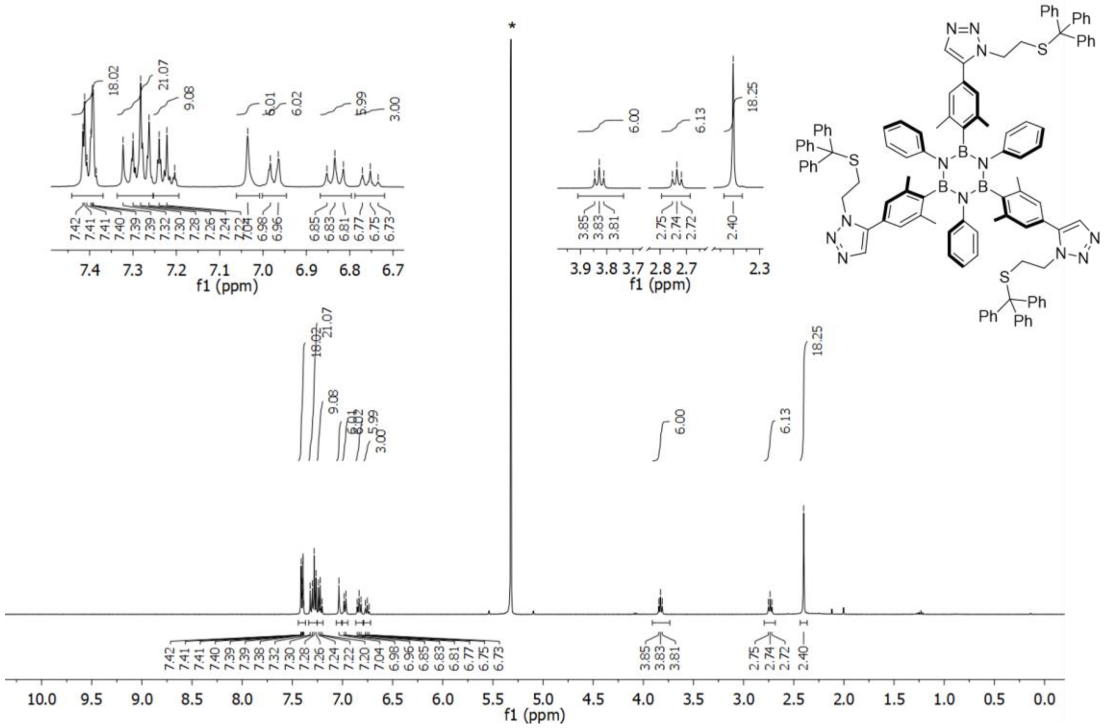

Figure S27. ^{1}H NMR (400 MHz, CD_2Cl_2) spectrum.

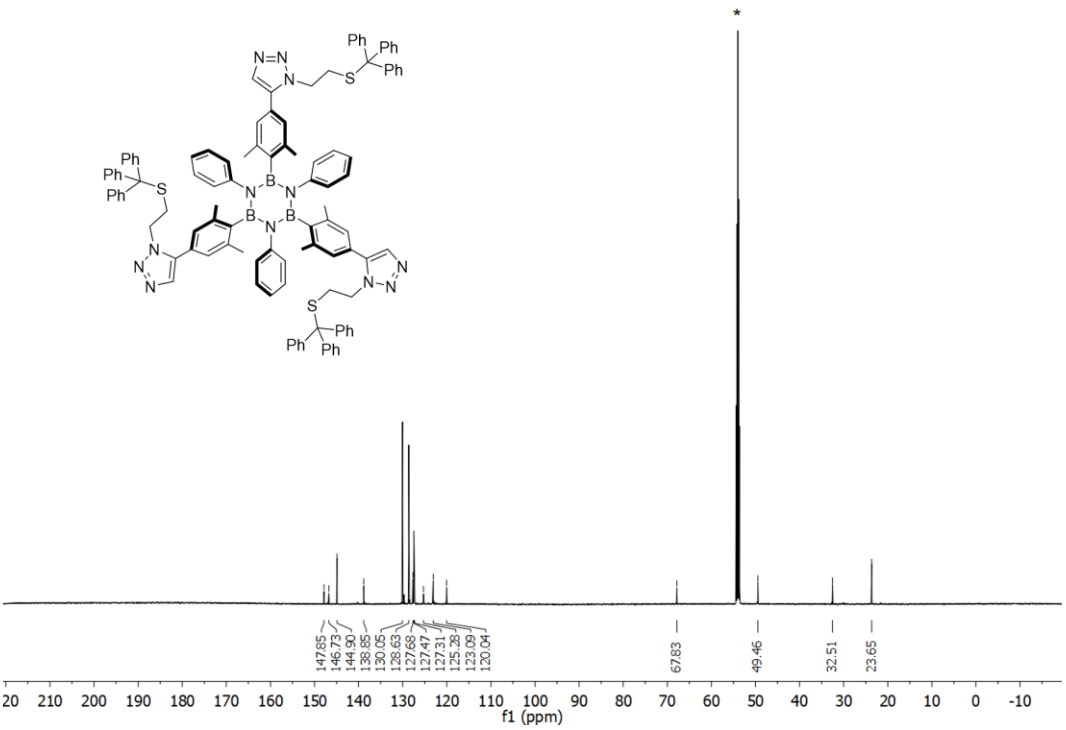

Figure S28. ^{13}C NMR (151 MHz, CD_2Cl_2) spectrum.

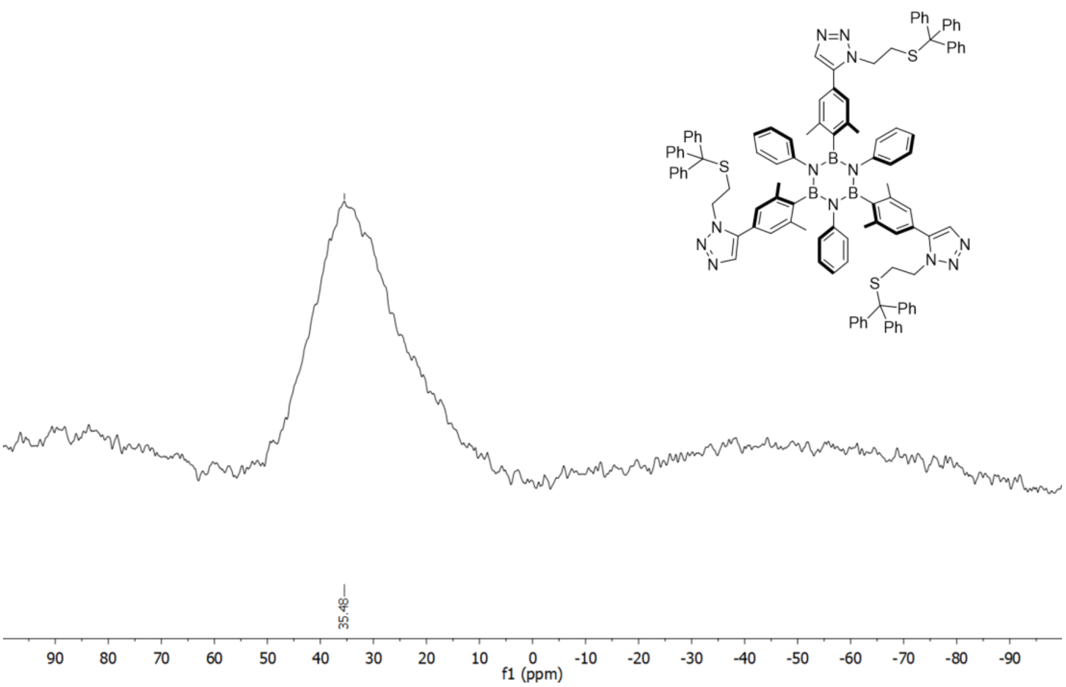

Figure S29. ^{11}B NMR (193 MHz, CD_2Cl_2) spectrum.

S3.12 Characterization of 2^{SH}

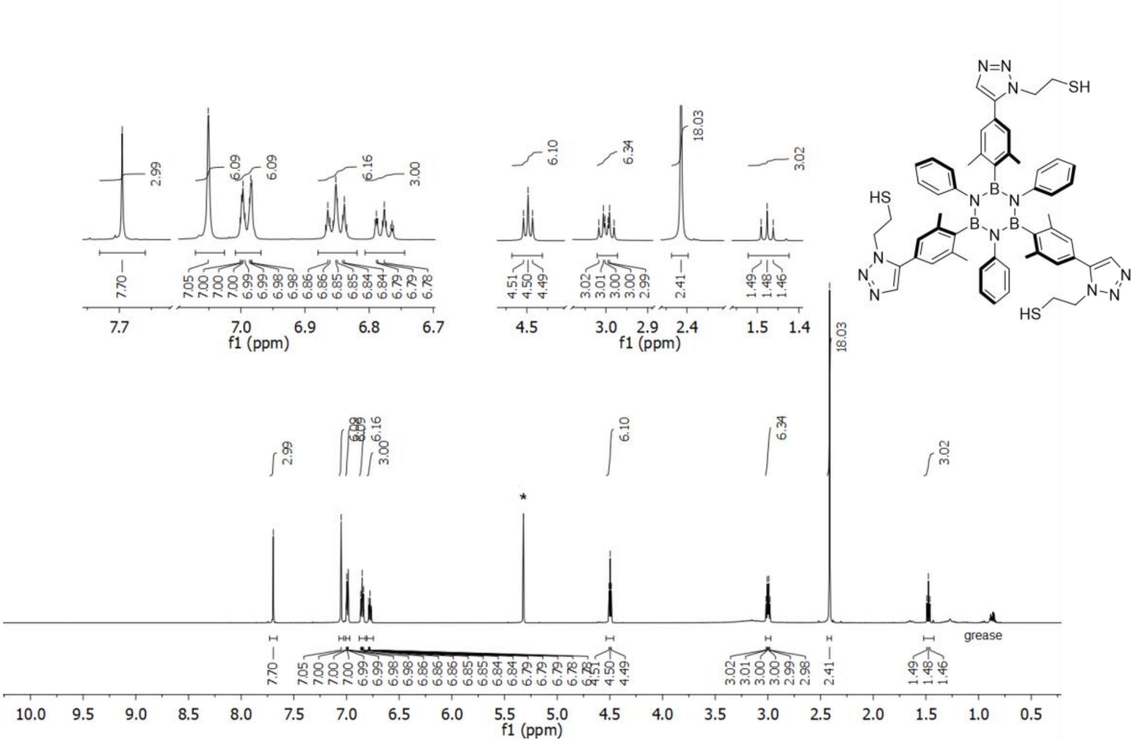

Figure S30. ^{1}H NMR (600 MHz, CD_2Cl_2) spectrum.

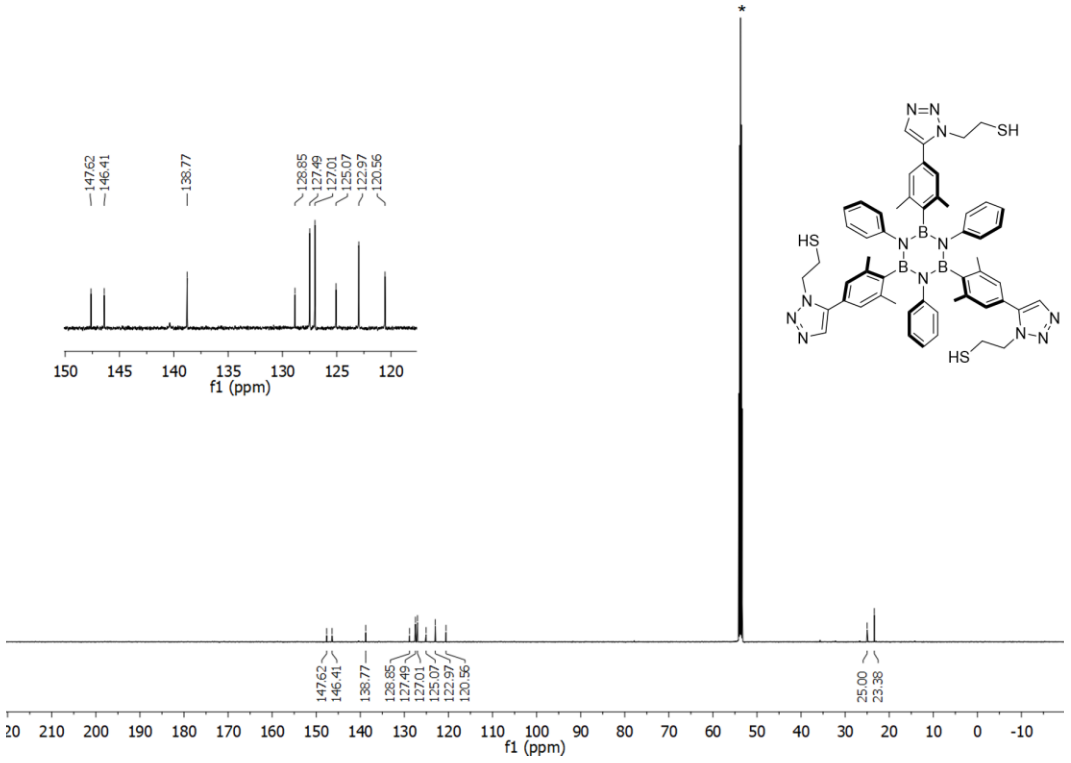

Figure S31. ^{13}C NMR (151 MHz, CD_2Cl_2) spectrum.

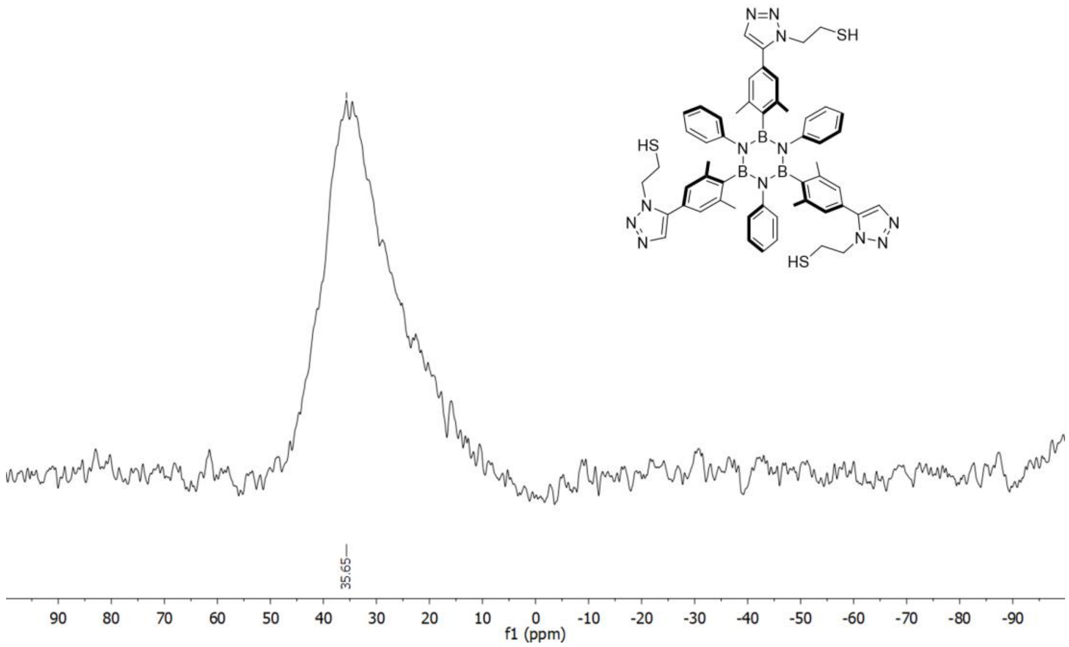

Figure S32. ^{11}B NMR (193 MHz, CD_2Cl_2) spectrum.

S4 Characterization of the BN-doped graphene films – extra material

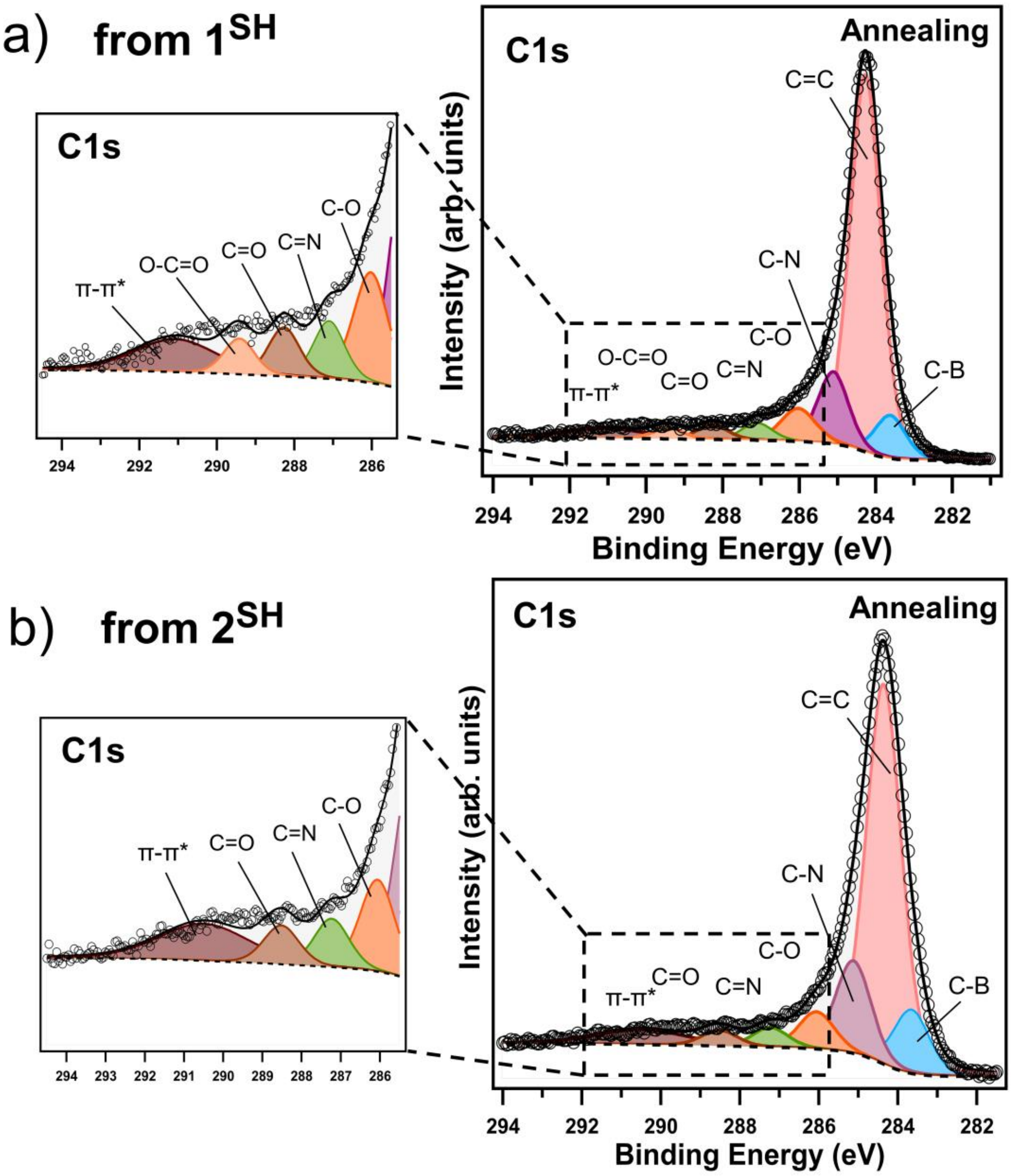

Figure S33. Enlargement of the XPS spectra of the C1*s* core level region of the BN-doped graphene films obtained by photopolymerization and annealing of SAMs of thiol borazine (a) $\mathbf{1}^{SH}$ and (b) $\mathbf{2}^{SH}$.

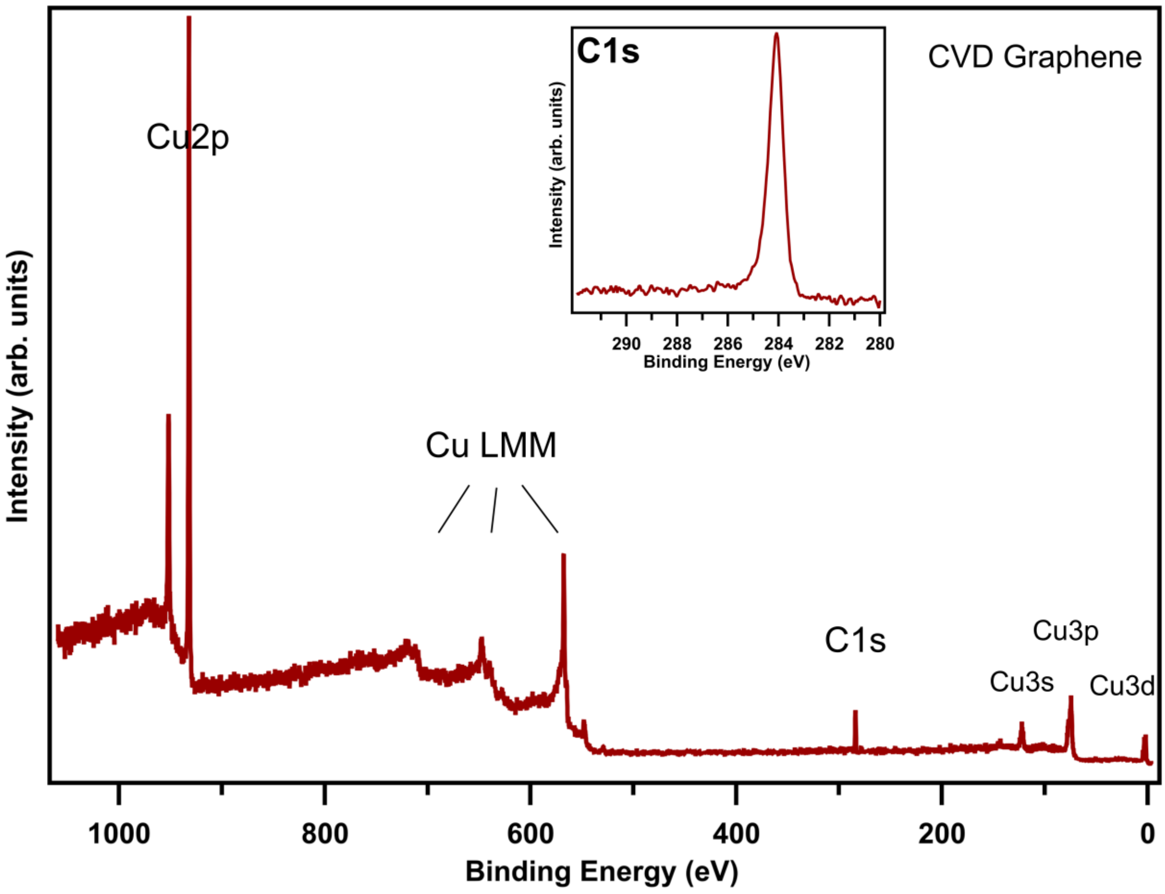

Figure S34. XPS of CVD-grown graphene on Cu foil: wide scan and in the inset the C1*s* core level region.

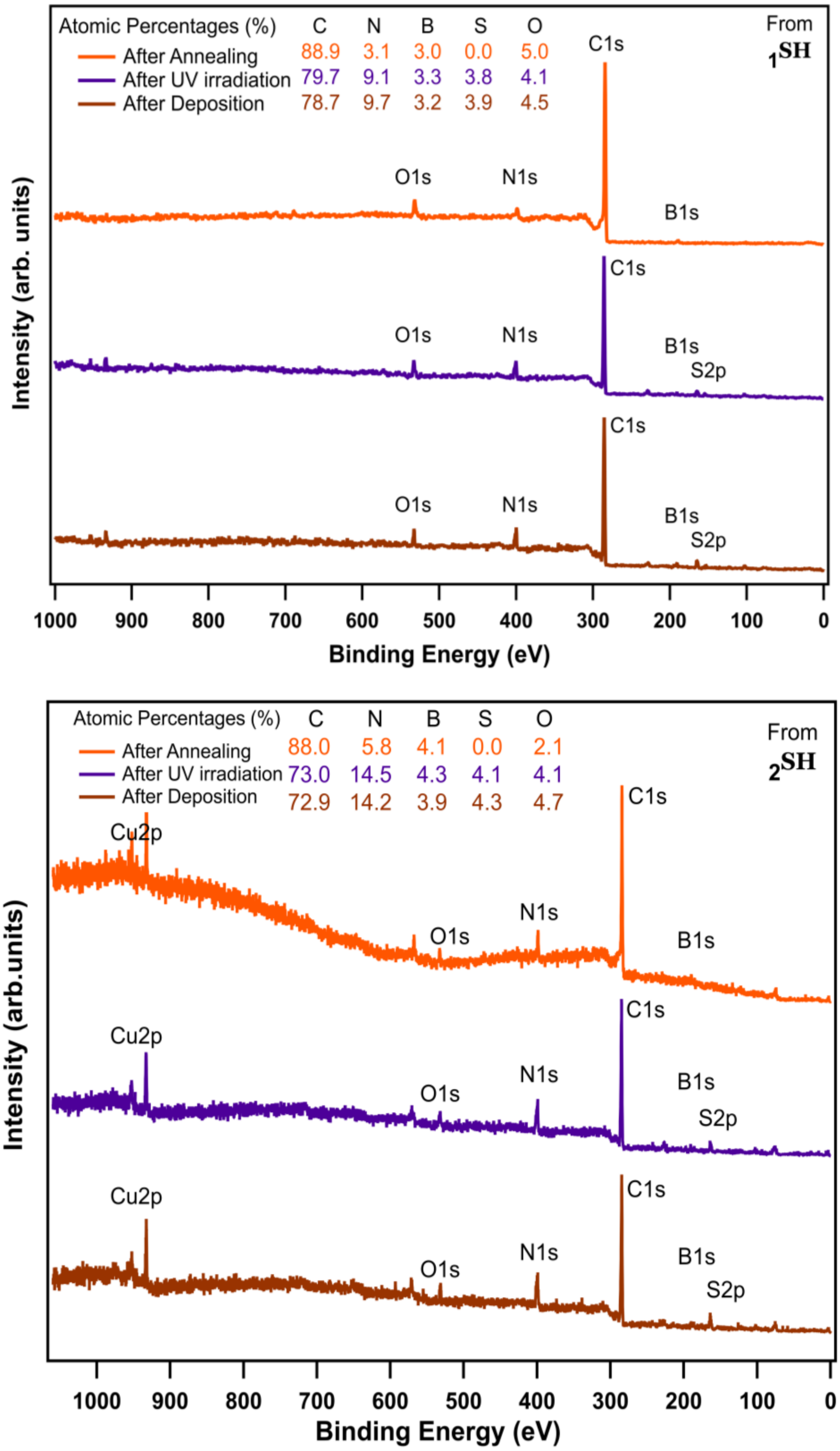

Figure S35. Wide scan XPS spectra after deposition, UV irradiation and annealing of **1**^{SH} and **2** ^{SH}. In the insets the composition in atomic percentages after each step is reported.

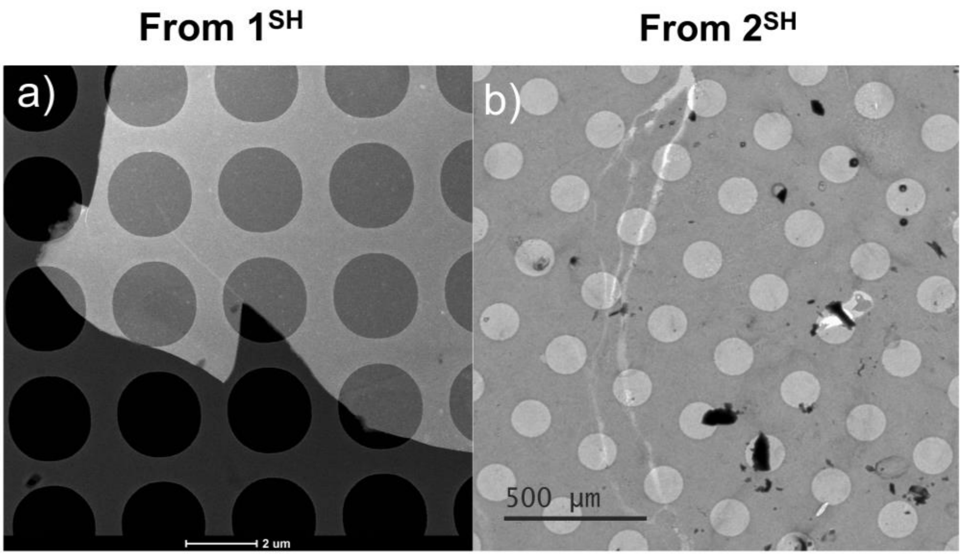

Figure S36. TEM micrographs of co-doped graphene samples resulting from annealing a polymerized SAM of **1**^{SH} (a) or **2**^{SH} (b) on electropolished copper foil; the images were collected after transfer to a TEM grid.

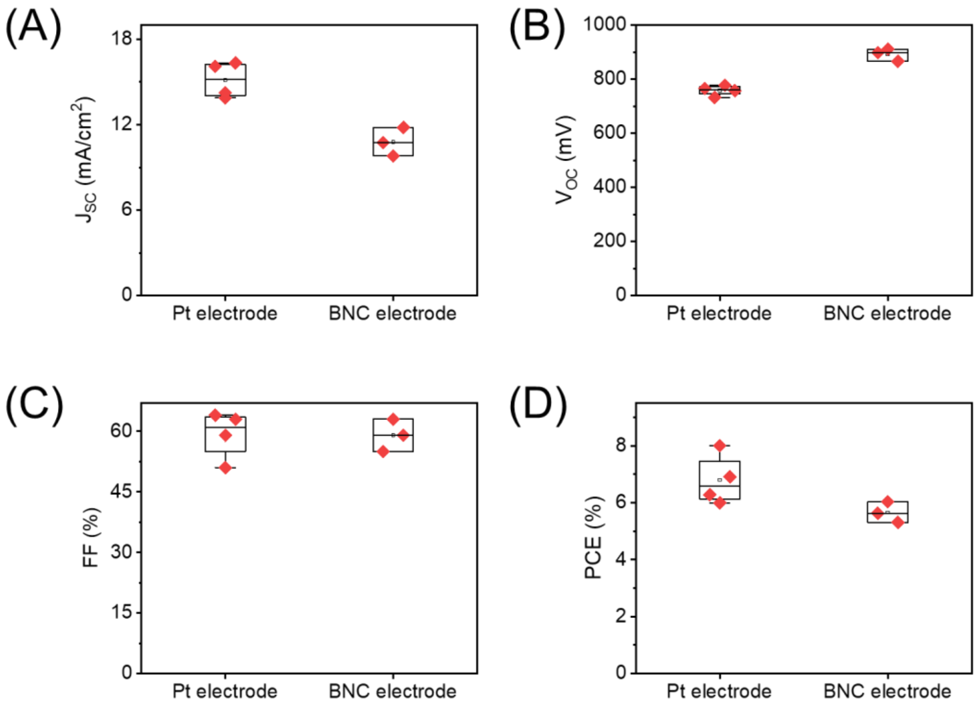

Figure S37. Device parameter distribution of DSSC devices fabricated with BNC electrode and Pt electrode

Table S1. Curve fitting parameters for the deconvoluted Raman spectra of the BNC films.

BNC film from 1^{SH}			
**Band**	**Position (cm^{-1})**	**FWHM (cm^{-1})**	**Area (%)**
**D***	1173	212	6.4
**D**	1331	202	58.2
**D”**	1466	162	14.6
**G**	1574	126	16.2
**D’**	1602	63	4.5
**BNC film from 2^{SH}**			
**Band**	**Position (cm^{-1})**	**FWHM (cm^{-1})**	**Area (%)**
**D***	1200	134	5.4
**D**	1338	190	61.5
**D”**	1459	138	8.1
**G**	1554	110	9.2
**D’**	1601	83	15.8

Table S2. Raman analysis of the BNC films: calculated intensity ratios between different bands

**Ratio**	**BNC film from 1^{SH}**	**BNC film from 2^{SH}**
I_D/I_G	3.60	6.67
$I_{D'}/I_G$	0.28	1.71

Table S3. The performances of the BN-doped graphene based materials in DSSCs in the representative literature.

Sample	J_{SC} (mA/cm^2)	V_{OC} (mV)	FF (%)	PCE (%)	Ref
BN-laser induced graphene as Counter Electrode	12.06	690	60	4.99	6
BN-Graphene Quantum dots/Carbon nanotubes as Counter Electrode	16.59	750	68	8.49	7
BN-doped Graphene layer as Counter Electrode	11.8	866	59	6.03	This Work

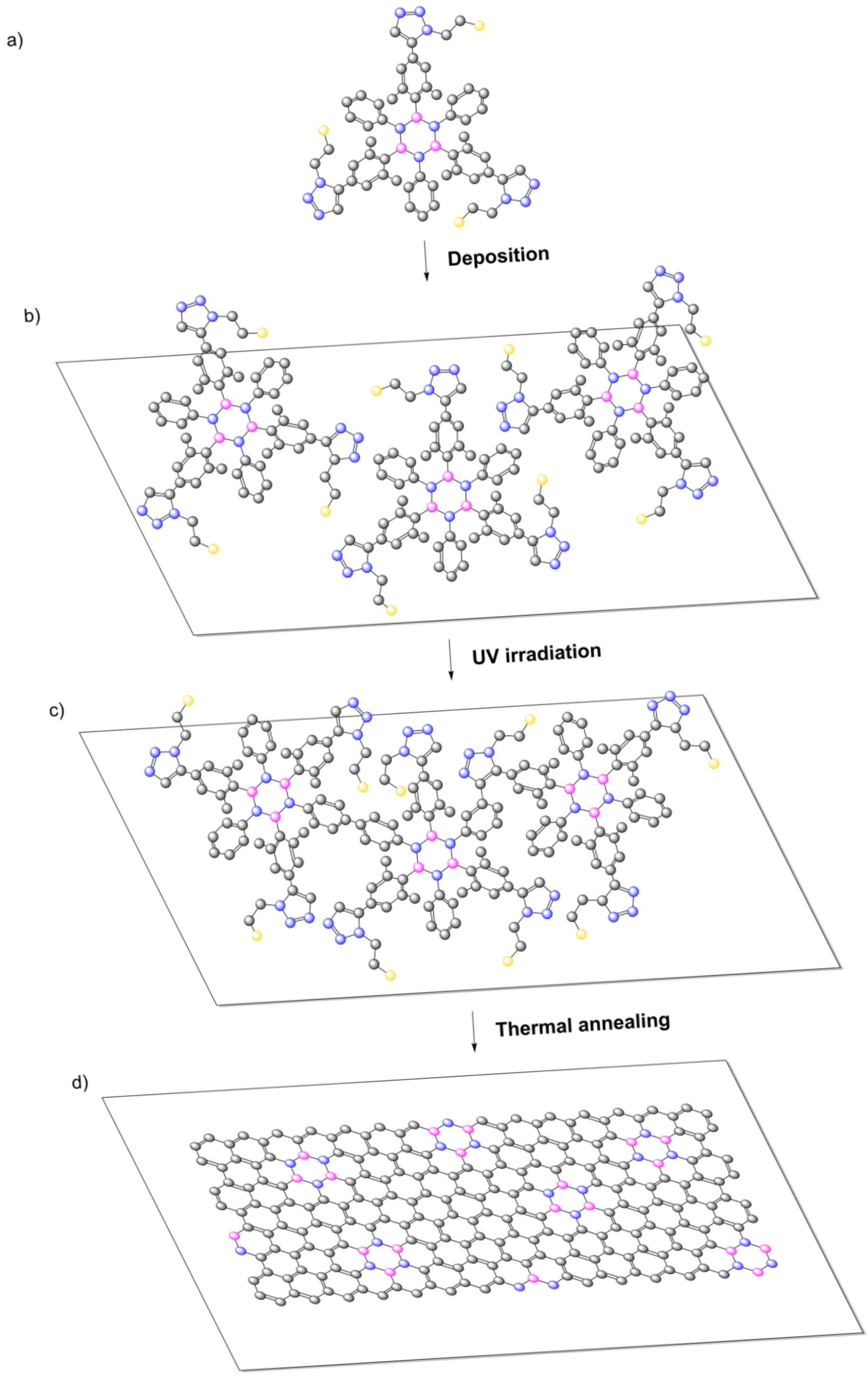

Scheme S1. Scheme of: the molecular precursor (2^{SH})(a), after deposition of self-assembled monolayer on the substrate (b), after UV light-induced polymerization (c) and after thermal conversion to transform it into BNC film (d).

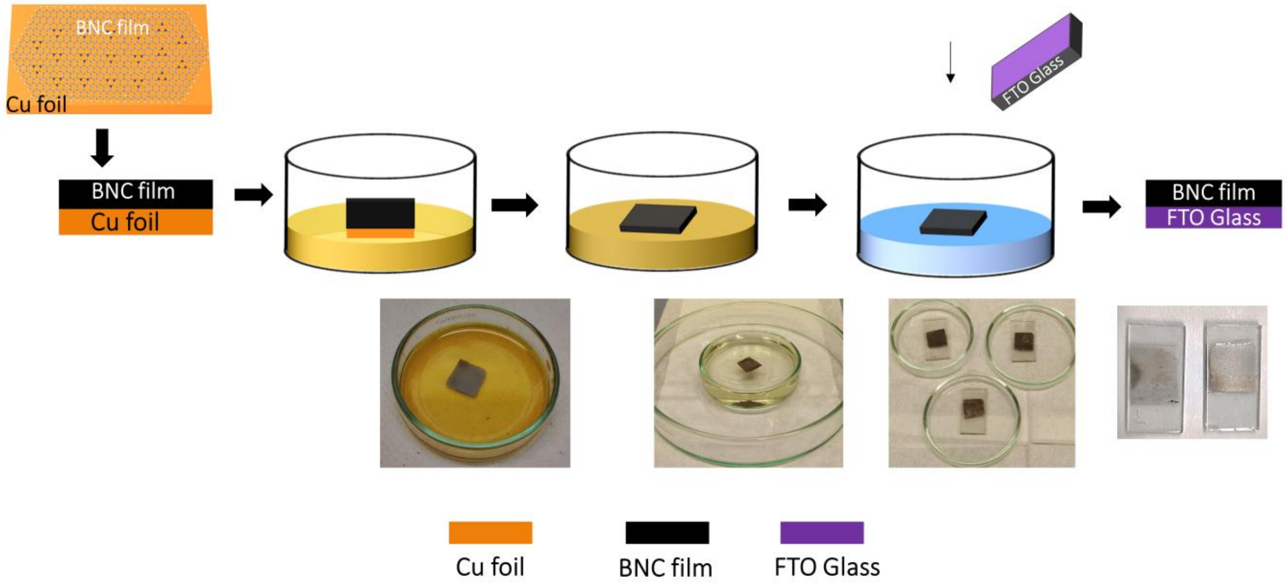

Scheme S2. Scheme of the BNC film transfer from Cu foils to FTO glass substrate.

S5 References

(1) Kalashnyk, N.; Ganesh Nagaswaran, P.; Kervyn, S.; Riello, M.; Moreton, B.; Jones, T. S.; De Vita, A.; Bonifazi, D.; Costantini, G. Self-Assembly of Decoupled Borazines on Metal Surfaces: The Role of the Peripheral Groups. *Chemistry – A European Journal* **2014**, *20* (37), 11856–11862. https://doi.org/10.1002/chem.201402839.

(2) Marinelli, D.; Fasano, F.; Najjari, B.; Demitri, N.; Bonifazi, D. Borazino-Doped Polyphenylenes. *J. Am. Chem. Soc.* **2017**, *139* (15), 5503–5519. https://doi.org/10.1021/jacs.7b01477.

(3) Yeo, W.; Min, D.; Hsieh, R. W.; Greene, G. L.; Mrksich, M. Label-Free Detection of Protein–Protein Interactions on Biochips. *Angew. Chem. Int. Ed.* **2005**, *44* (34), 5480–5483. https://doi.org/10.1002/anie.200501363.

(4) Slor, G.; Papo, N.; Hananel, U.; Amir, R. J. Tuning the Molecular Weight of Polymeric Amphiphiles as a Tool to Access Micelles with a Wide Range of Enzymatic Degradation Rates. *Chem. Commun.* **2018**, *54* (50), 6875–6878. https://doi.org/10.1039/C8CC02415D.

(5) Rocard, L.; Berezin, A.; De Leo, F.; Bonifazi, D. Templated Chromophore Assembly by Dynamic Covalent Bonds. *Angew. Chem. Int. Ed.* **2015**, *54* (52), 15739–15743. https://doi.org/10.1002/anie.201507186.

(6) Yang, B.; Guan, Y.; Lu, Y.; Chu, Z. Laser-Induced Nitrogen and Boron Co-Doped Graphene Film for Dye-Sensitized Solar Cell Applications. *Materials Letters* **2024**, *366*, 136505. https://doi.org/10.1016/j.matlet.2024.136505.

(7) Lin, K.-Y.; Cai, M.-Q.; Wu, Y.-T.; Yeh, M.-H.; Jiang, J.-C. Boron and Nitrogen Codoped Multilayer Graphene as a Counter Electrode: A Combined Theoretical and Experimental Study on Dye-Sensitized Solar Cells under Ambient Light Conditions. *J. Phys. Chem. C* **2021**, *125* (45), 24894–24901. https://doi.org/10.1021/acs.jpcc.1c06646.
